# Lipid metabolism of plasma-derived small extracellular vesicles in COVID-19 convalescent patients

**DOI:** 10.1038/s41598-023-43189-5

**Published:** 2023-10-03

**Authors:** Wenjing Xiao, Qi Huang, Ping Luo, Xueyun Tan, Hui Xia, Sufei Wang, Yice Sun, Zhihui Wang, Yanling Ma, Jianchu Zhang, Yang Jin

**Affiliations:** 1 Department of Respiratory and Critical Care Medicine, Hubei Province Clinical Research Center for Major Respiratory Diseases, NHC Key Laboratory of Pulmonary Diseases, Union Hospital, Tongji Medical College, Huazhong University of Science and Technology, Wuhan, 430022 Hubei China; 2 MOE Key Laboratory of Biological Targeted Therapy, Union Hospital, Tongji Medical College, Huazhong University of Science and Technology, Wuhan, 430022 Hubei China; 3 Department of Translational Medicine Center, Union Hospital, Tongji Medical College, Huazhong University of Science and Technology, Wuhan, 430022 Hubei China; 4 Department of Scientific Research, Union Hospital, Tongji Medical College, Huazhong University of Science and Technology, Wuhan, 430022 Hubei China; 5 Hubei Province Engineering Research Center for Tumor-Targeted Biochemotherapy, Wuhan, 430022 China

**Keywords:** Public health, Clinical trials

## Abstract

The coronavirus disease 2019 (COVID-19), which affects multiple organs, is causing an unprecedented global public health crisis. Most COVID-19 patients recover gradually upon appropriate interventions. Viruses were reported to utilize the small extracellular vesicles (sEVs), containing a cell-specific cargo of proteins, lipids, and nucleic acids, to escape the attack from the host’s immune system. This study aimed to examine the sEVs lipid profile of plasma of recovered COVID-19 patients (RCs). Plasma sEVs were separated from 83 RCs 3 months after discharge without underlying diseases, including 18 recovered asymptomatic patients (RAs), 32 recovered moderate patients (RMs), and 33 recovered severe and critical patients (RSs), and 19 healthy controls (HCs) by Total Exosome Isolation Kit. Lipids were extracted from sEVs and then subjected to targeted liquid chromatography-mass spectrometry. The size, concentration, and distribution of sEVs did not differ in RCs and HCs as validated by transmission electron microscopy, nanoparticle tracking analysis, and immunoblot analysis. Fifteen subclasses of 508 lipids were detected in plasma sEVs from HCs, RAs, RMs, and RSs, such as phosphatidylcholines (PCs) and diacylglycerols (DAGs), etc. Total lipid intensity displayed downregulation in RCs compared with HCs. The relative abundance of DAGs gradually dropped, whereas PCs, lysophosphatidylcholines, and sphingomyelins were higher in RCs relative to HCs, especially in RSs. 88 lipids out of 241 in sEVs of RCs were significantly different and a conspicuous increase was revealed with disease status. The sEVs lipids alternations were found to be significantly correlated with the clinical indices in RCs and HCs, suggesting that the impact of COVID-19 on lipid metabolism lingered for a long time. The lipid abnormalities bore an intimate link with glycerophospholipid metabolism and glycosylphosphatidylinositol anchor biosynthesis. Furthermore, the lipidomic analysis showed that RCs were at higher risk of developing diabetes and sustaining hepatic impairment. The abnormality of immunomodulation in RCs might still exist. The study may offer new insights into the mechanism of organ dysfunction and help identify novel therapeutic targets in the RCs.

## Introduction

With its staggering infection rate and high mortality, the pandemic of coronavirus disease 2019 (COVID-19) has reached a magnitude of catastrophe^1–3^. To date, most of the COVID victims globally have gradually recovered. However, some recovered COVID-19 patients (RCs) developed sequelae, including pulmonary fibrosis, cardiac injury, olfactory impairment, mental disorders, etc., at their early recovery stage^4–6^. Therefore, rehabilitation and protective intervention are of great importance for convalescent patients.

Small extracellular vesicles (sEVs), a vital subtype of extracellular vesicles (EVs) with a diameter of 50–200 nm, have acted as an intercellular communication bridge via carrying molecular cargos^7,8^. Due to these capabilities, sEVs can regulate cellular communication, growth, migration, angiogenesis, and immunity^7,9,10^. sEVs have been previously reported to promote viral spreading and enhance the detrimental effect of viruses ^7,11^. For instance, sEVs from HIV-1-infected cells could reactivate HIV-1 in latently infected cells^12^. COVID-19 patient-derived sEVs were reportedly found to contain several lipids that were complicated in the immune and inflammatory response and activation of the coagulation and complement, which were the vital mechanisms underlying the COVID-related multiple organ dysfunctions^13^. Thus, fully understanding the changes in sEVs from RCs is of great significance and can help develop rehabilitation strategies and management for sequelae.

Lipids are a set of essential biomolecules critical to membrane structure and energy storage^14,15^. Mounting evidence has indicated that lipids have a wide range of crucial biologic functions in cellular homeostasis, energy conversion, material transport, information recognition, and cell development and differentiation^16–18^. Previous studies have demonstrated dramatic alternation in lipid profiles after virus infectious diseases as found in SARS and Ebola^19, 20^. For example, Chen et al. detected lipids in recovered SARS patients 12 years after infection and revealed significant differences in the serum metabolomes of SARS survivors^19^. Horkai D et al. analyzed the lipids of rhinoviruses-infected cells and found multiple differential lipid metabolic pathways, which could help uncover potential antiviral drug targets^21^. It has been reportedly that lipid dysregulation still exists in COVID-19 after discharge. Di Wu revealed that fundamental lipids had not returned to normal by the time they were discharged^22^. Sin Man Lam conducted high-coverage lipidomic of sEVs from patients at different temporal stages of COVID-19 and suggested dysregulated raft lipid metabolism^23^. Furthermore, we have shown that plasma lipid profiling is also present in discharged COVID-19 patients with pulmonary sequelae^24^. Hence, the study aimed to reveal the differential changes to correlate lipid alternations with COVID-19 co-morbidities that can help us better monitor the condition of patients undergoing recovery.

In the present study, we examined the lipids of plasma sEVs isolated from RCs without underlying diseases 3 months after discharge and healthy controls (HCs). Our findings demonstrated that these discharged patients have not fully recovered from the physiological impact of COVID-19 infection. We revealed that the abnormality of the liver, glucose, energy metabolism, and immunomodulation in RCs might be present.

## Results

### Clinical characteristics

We enrolled 102 participants, including 18 recovered asymptomatic patients (RAs), 32 recovered moderate patients (RMs), 33 recovered severe and critical patients (RSs), and 19 HCs. The demographic characteristics are summarized in Table S1. Serological testing of the RCs sample showed positive for the IgG or IgM, indicating the solid humoral immune responses of COVID-19 survivors during recovery. Table S1 shows that most of the clinical indices of RCs had returned to normal. For example, C-reactive protein (CRP), a marker of systemic inflammation and indicator of poor prognosis, was significantly higher in moderate patients (Ms) and severe and critical patients (Ss) than HCs, Ms than RMs, and Ss than RSs and had returned to normal levels as compared to that of HCs^25,26^. Lymphocytes and monocytes, two critical players in antiviral immune response, decreased in M and patients and gradually rose to control levels. In addition, clinical indices, including hepatic, renal, and cardiac functions, restored to average relative to HCs. In contrast to HCs, apparent abnormalities in electrolytes (P and Mg) and coagulation indicators (PT and APTT) were observed in RCs.

### Characterization of small extracellular vesicles (sEVs)

**Figure 1:**
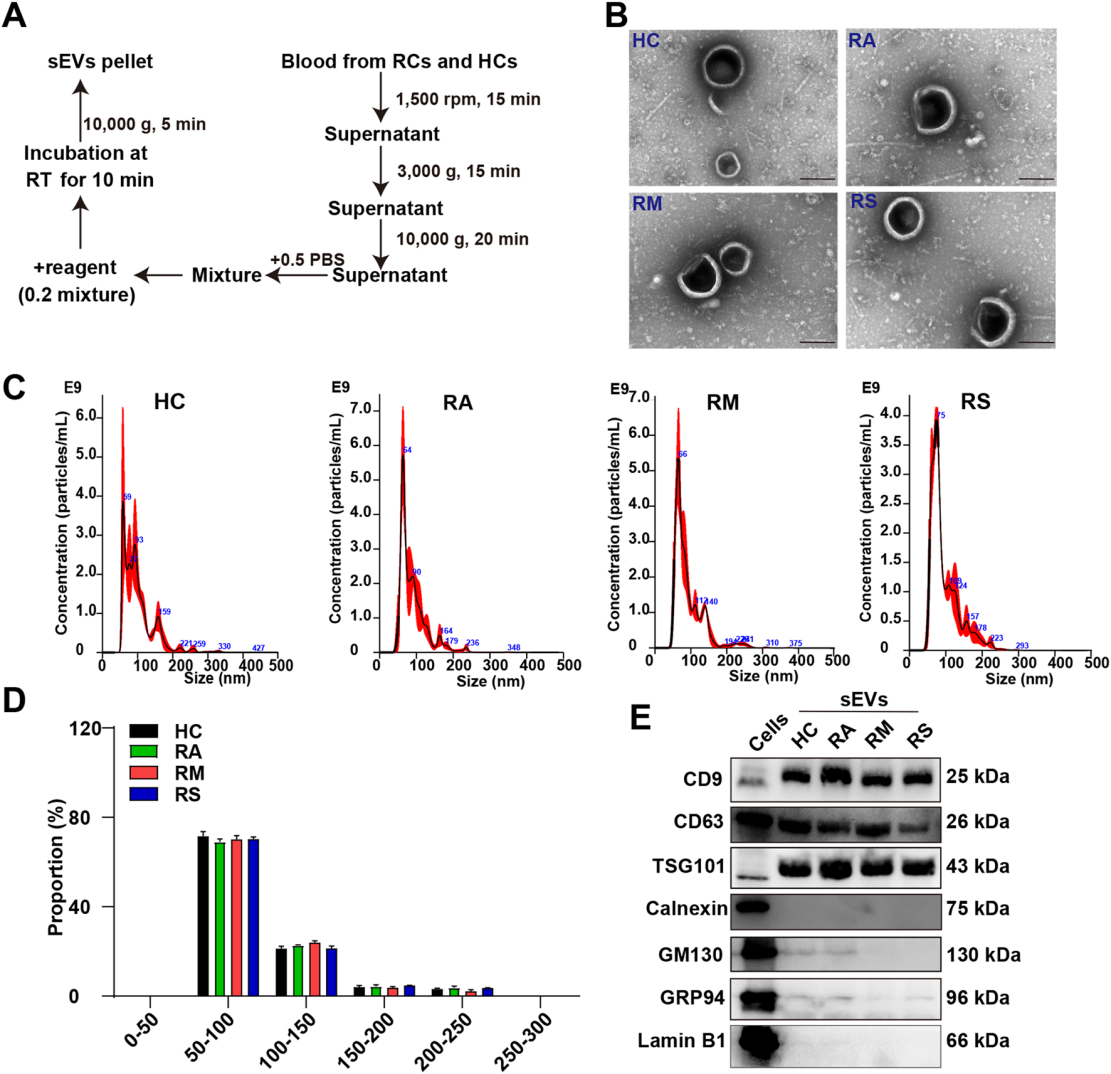
Validation of small extracellular vesicles. (**A**) The workflow of the isolation of small extracellular vesicles (sEVs) from the plasma of healthy controls (HCs) and recovered COVID-19 patients (RCs), including recovered asymptomatic patients (RAs), recovered moderate patients (RMs), and recovered severe and critical patients (RSs). (**B**) Representative transmission electron microscopy of the morphology of sEVs using commercial Exosome kit from RAs, RMs, RSs, and HCs. Scale bar: 200 nm. (**C**) Nanoparticle tracking analysis of sEVs from the plasma of RAs, RMs, RSs, and HCs. The detailed data were presented in Tables S2 and S3. (**D**) The size distribution of the purified sEVs isolated from plasma of HCs, RAs, RMs, and RSs. (**E**) Western blotting indicates the expression levels of CD9, CD63, TSG101, Calnexin, GM130, GRP94, and Lamin B1 in cells, RAs, RMs, RSs, and HCs. Among them, cells were selected as control. The gels were cropped from different parts and automatically exposed using BIORAD. Cropped blots were displayed alongside their corresponding original blots in Fig. S2.

sEVs isolation from RCs and HCs plasma was conducted using the commercial Exosome kit (Thermo Fish Scientific) per the manufacturer’s protocols (Fig. 1A). Characterization of sEVs was performed by transmission electron microscopy (TEM), nanoparticle tracking analysis (NTA), nanoflow analysis, and western blotting. Cup-shaped vesicles with diameters less than 200 nm were observed via TEM (Fig. 1B). Size distribution of sEVs ranged between 50 and 200 nm revealed by NTA (Fig. 1C,D). Our data did not show a significant difference in the concentration, mean size, and mode size of sEVs between the four groups (Fig. S1A and Tables S2, S3). Moreover, nanoflow analysis further elucidated the size distribution (Fig. S1B). Western blotting assay showed the expression of sEVs markers (CD9, CD63, and TSG101). Contrariwise, sEVs isolated from plasma did not contain GRP94, Lamin B1, calnexin, and GM130 (Fig. 1E and Fig. S2). Collectively, these results suggested that sEVs were successfully isolated from the plasma of RAs, RMs, RSs, and HCs.

### Distinct lipids of RCs compared with HCs

**Figure 2:**
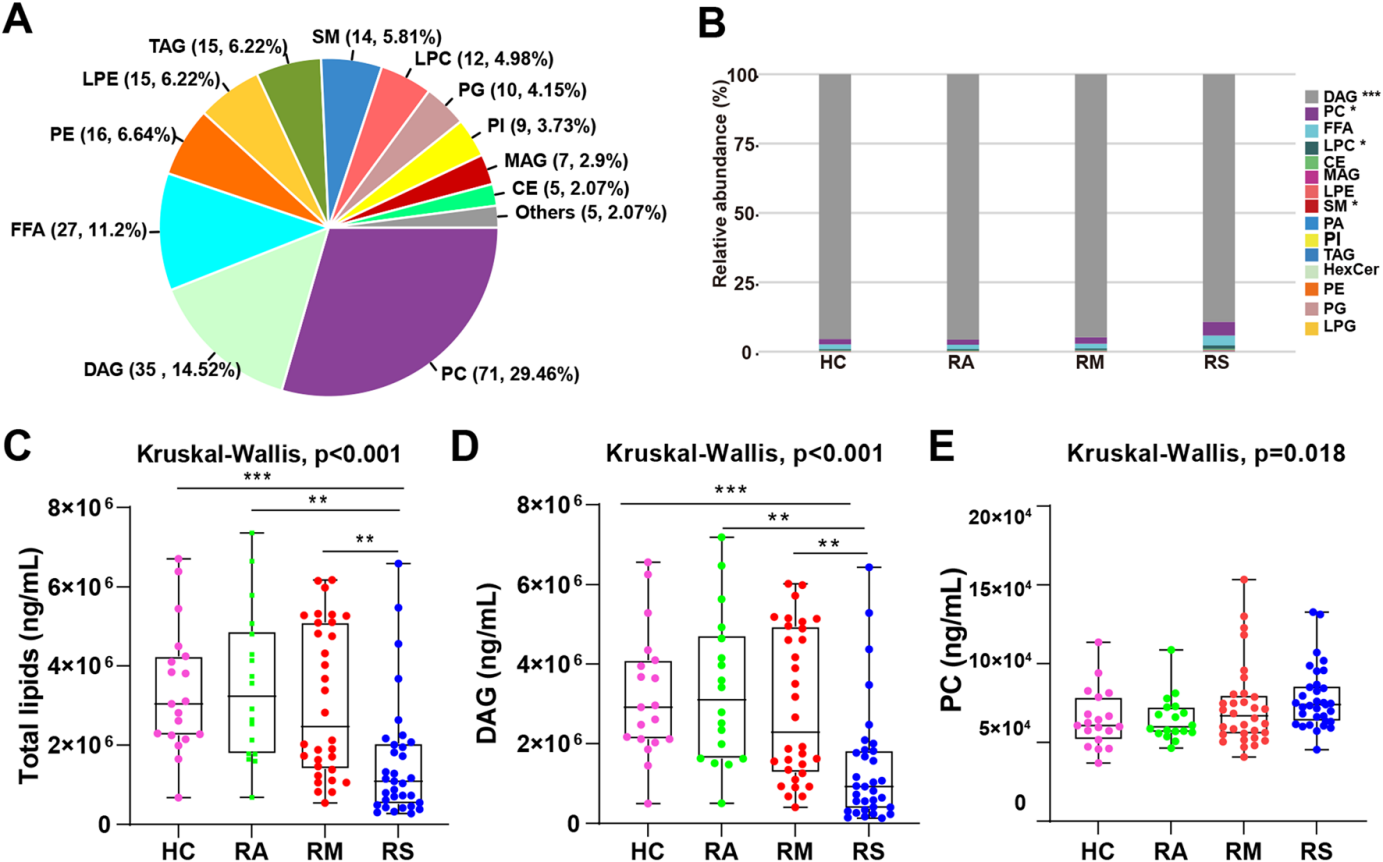
(**A**) Percentage of identified sEVs cargo lipid classes and subclasses. (**B**) Relative abundance (%) of sEVs lipids subclasses in recovered asymptomatic patients (RAs), recovered moderate patients (RMs), recovered severe/critical patients (RSs), and healthy controls (HCs). (**C**–**E**) The level of total lipids and lipids subclasses diacylglycerols (DAGs) and phosphatidylcholines (PCs) in RAs, RMs, RSs, and HCs. Data were displayed as boxplots with median and range and each dot represents an individual: HC (Pink), RA (Green), RM (Red), RS (Blue). p-value: *p < 0.05; **p < 0.01; ***p < 0.001.

Targeted lipidomics was conducted in the extracted sEVs. Totally, 508 lipids, involving 15 lipid subclasses, were quantitatively determined (Table S4 and Fig. 2A). These lipid subclasses included free fatty acids (FFAs), phosphatidylcholines (PCs), lysophosphatidylcholines (LPCs), diacylglycerols (DAGs), lysoPhosphatidylethanolamines (LPEs), triacylglycerols (TAGs), sphingomyelins (SMs), and monoacylglycerols (MAGs) (Fig. S3A). The quality control (QC) samples from each group were closely centralized around the center of the PCA score plot (Fig. S3B). Figure S3C showed that the correlation coefficient of QC samples was close to one, indicating repeatability and stability. We found that abundance of sEVs cargo lipid subclasses, such as DAGs and PCs, were significantly different in RCs relative to HCs (Fig. 2B). The sEVs total lipids displayed downregulation in RCs compared with HCs (Fig. 2C). Among the subclasses, DAGs of sEVs made up more than 90% of total lipids and the intensity of DAGs in the RSs was significantly reduced relative to HCs, which was consistent with changes in the abundance of DAGs (Fig. 2D and Fig. S3D). On the contrary, the content of PCs, SMs, and LPCs was increased in RCs compared with HCs using Kruskal–Wallis (Fig. 2E and Fig. S3E).

**Figure 3:**
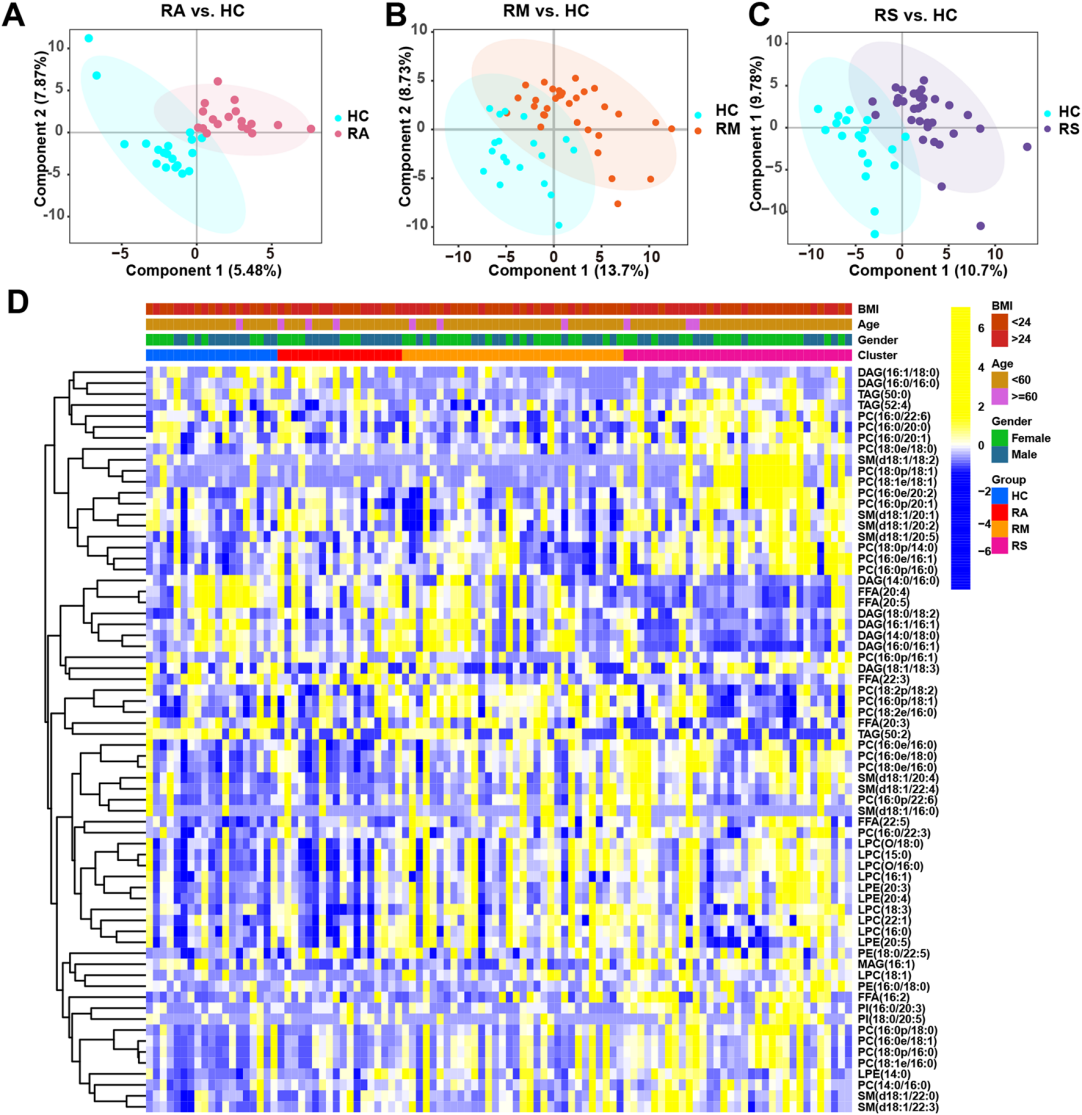
Lipid profiles of sEVs isolated from HCs, RAs, RMs, and RSs plasma. (**A**–**C**) Orthogonal partial least square discriminant analysis score plot in recovered asymptomatic patients (RAs), recovered moderate patients (RMs), and recovered severe/critical patients (RSs) compared to HCs. (**D**) The heatmap shows significantly altered lipids in the sEVs isolated from the plasma of RAs, RMs, RSs, and HCs. Only differential lipids with p < 0.05 were displayed, and the color shades indicated the levels of lipids. Yellow and blue are indicative of relatively higher and lower levels, respectively.

**Figure 4:**
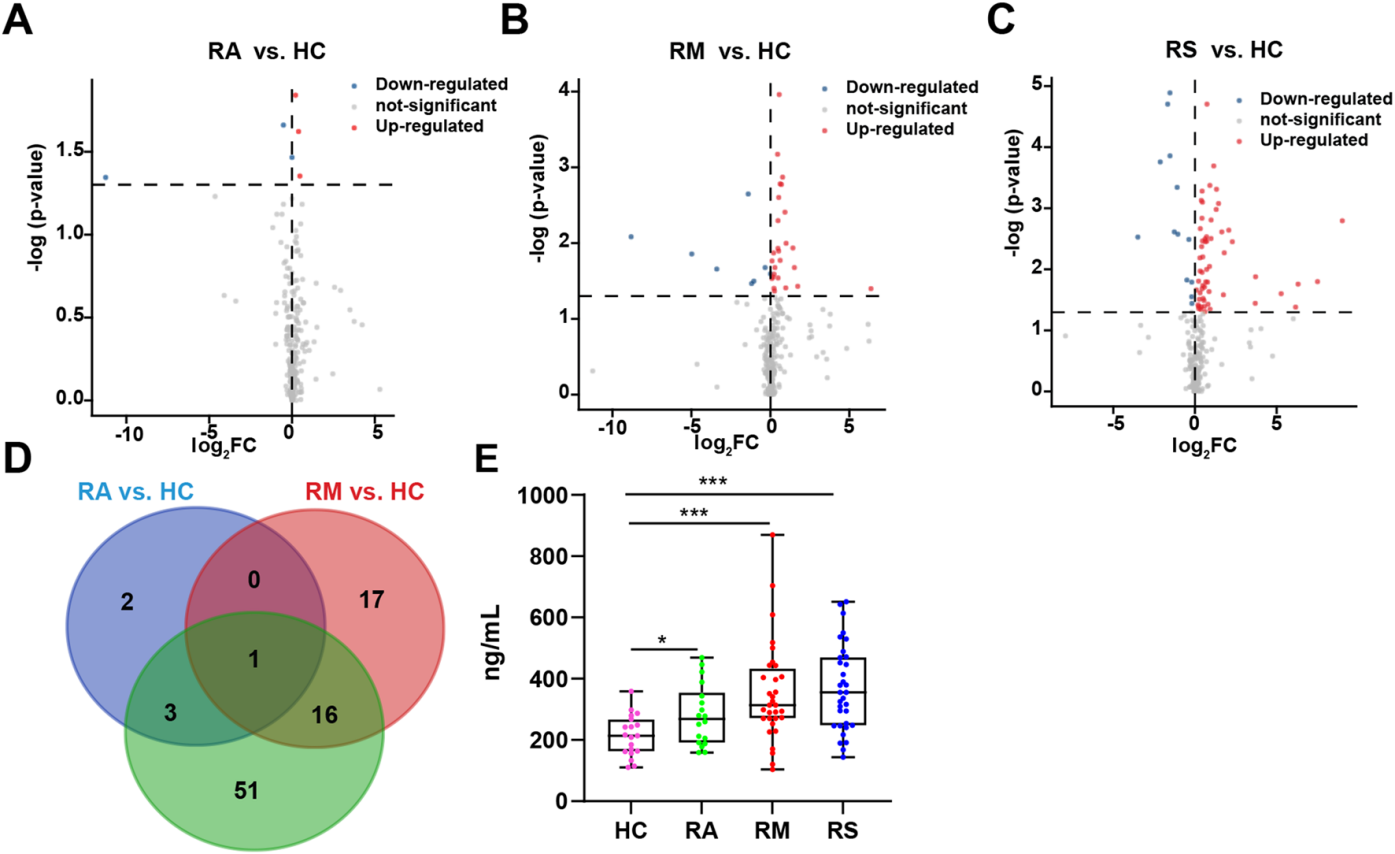
(**A**–**C**) Volcano plots show the sEVs differential lipids in RAs (**A**), RMs (**B**), or RSs (**C**) compared with HCs. The x-axis was the value of log2(FC), FC indicates the ratio of the mean level of the sEVs lipid in RAs, RMs, or RSs to the mean value of HCs, y-axis was − lg(p-value). Blue represented downregulation with p < 0.05, red represented upregulation, and grey denoted no change. (**D**) Venn diagram shows the number of significantly altered lipids in RAs, RMs, and RSs relative to HCs. (**E**) Statistical analysis of FFA(22:5) was displayed as bar graphs in sEVs of HCs, RAs, RMs, and RSs. The significance of comparisons was determined by the Kruskal–Wallis test. Data were displayed as boxplots with median and range and each dot represents an individual: HC (Pink), RA (Green), RM (Red), RS (Blue). Lipids quantitated using targeted lipidomics were presented in nanograms of lipids per milliliter (ng/mL) sEVs. p-value: *p < 0.05; **p < 0.01; ***p < 0.001. *RAs* recovered asymptomatic patients, *RMs* recovered moderate patients, *RSs* recovered severe/critical patients.

The PCA score plot failed to distinguish RAs, RMs, and RSs from HCs (Fig. S4A–C). Then, the PLS-DA score plot showed that the sEVs lipids from RCs (RAs, RMs, and RSs) were away from HCs (Fig. 3A–C). The lipid of RAs, RMs, and RSs varied substantially (Figs. 3D and 4A–C). In the RA vs. HC group, only 6 differential lipid profiles were found in sEVs (Fig. 4A). In RMs and RSs, 35 and 71 lipids were markedly altered as compared with HCs, indicating that the number of lipids increased progressively with increasing severity of COVID-19 (Fig. 4B,C and Table S5). We noticed that the common differential lipid FFA (22:5) was persistently upregulated with the exacerbation of COVID-19 as compared to HCs, possibly suggesting the augmented secretion of FFA (22:5) to circulation (Fig. 4D,E).

Most lipid subclasses, including LPCs, LPEs, PCs, PEs, and SMs, were elevated in RA, RM, and RS as compared with HCs, except for PC(16:0/20:3) and PC(16:0/20:3), PC(16:0p/20:0), and PC(18:0p/18:0) in RS vs. HC, which were reduced (Table S5 and Fig. 3D). Elevation in sEVs SMs and some FFAs was in accordance with the results observed in the West Nile virus and ARDS ^27, 28^. These results indicated that the lipidomic disturbance in sEVs induced by COVID-19 infection persisted during recovery. In addition, most of the DAGs were significantly decreased, except for DAG(18:0/18:2) in the RM vs. HC group and DAG(14:0/18:2) in the RS vs. HC group. Among the differential lipids, PC(18:1e/18:1) displayed the greatest log_2_FC(+ 9.02) in PCs, SM(d18:1/22:4) exhibited the substantial log_2_FC(+ 7.51) in SMs, and DAG(14:0/18:2) showed the highest log_2_FC(+ 6.31) in DAGs in RS vs. HC.

We then performed an overlapping analysis in RM vs*.* HC and RS vs*.* HC and identified 19 differential lipids (Fig. S5A). Interestingly, two out of these differential lipids, DAG(14:0/16:0) and FFA(22:3) were found to be conspicuously reduced (Fig. S5B). The remaining fifteen lipids, such as LPC(15:0), LPC(18:0), PC(16:0e/16:0), and PC(16:0e/18:0), displayed upregulation (Fig. S5C,D).

**Figure 5:**
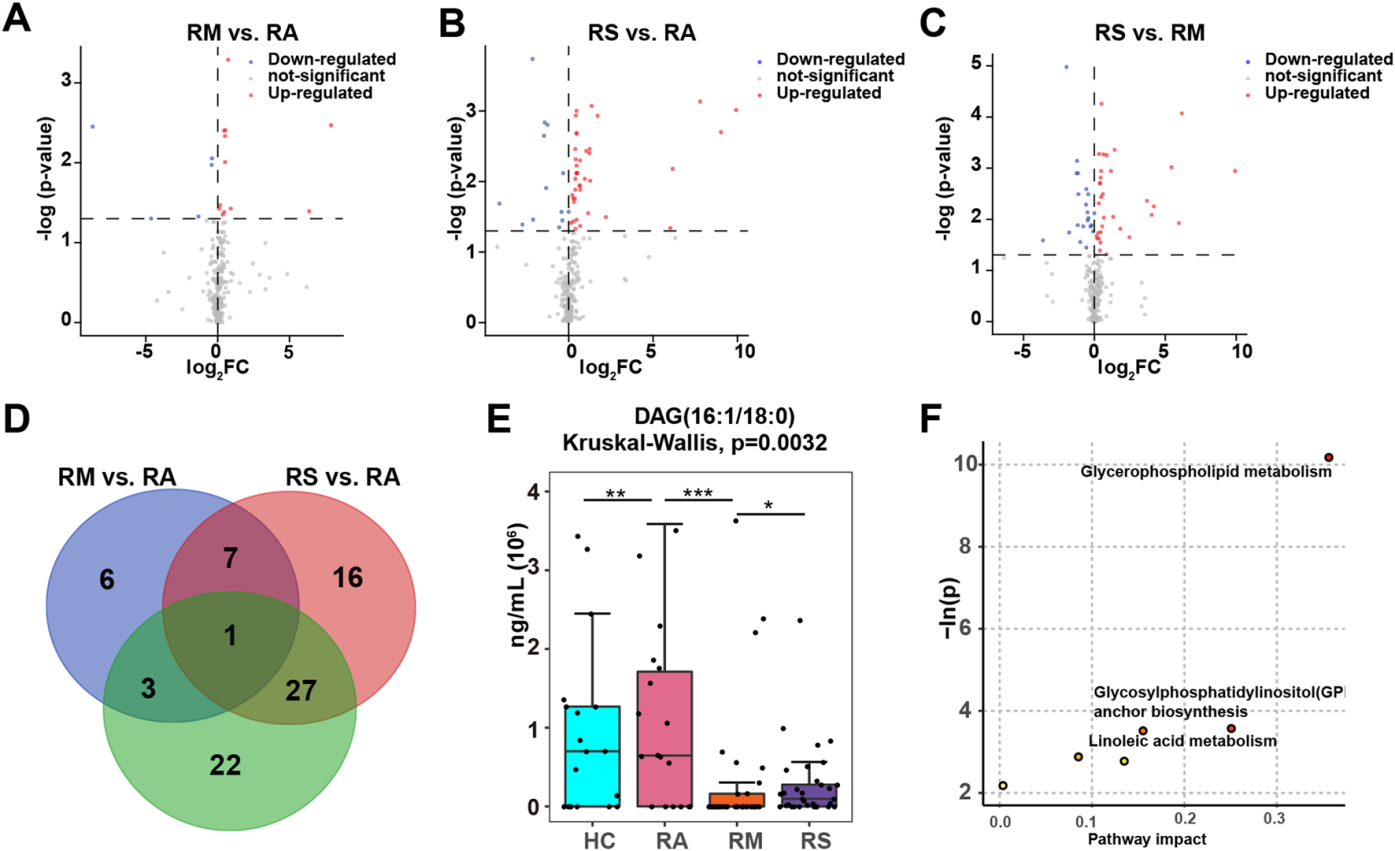
Distinct lipid profiles in RCs (RAs, RMs, and RSs) and KEGG enrichment analysis. (**A**–**C**) Volcano plots presenting differential lipids in RM vs. RA, RS vs. RA, and RS vs. RM. The x-axis is the log2(FC) value, and the y-axis is the − lg (p-value). Blue represented downregulation with p < 0.05, red represented upregulation, and grey denoted no change. (**D**) Venn plot showing the differential lipids among diverse RC groups (RAs, RMs, and RSs). The blue, red, and green circles represent RM vs. RA, RS vs. RA, and RS vs. RM groups. (**E**) Statistical analysis of DAG(16:1/18:0) is displayed as the box plot graph. The significance of comparisons was determined by the Kruskal–Wallis test. Lipids quantitated using targeted lipidomics were presented in nanomoles of lipids per milliliter (ng/mL) sEVs. p-value: *p < 0.05; **p < 0.01; ***p < 0.001. (**F**) Lipidomic KEGG enrichment analysis of differential lipids in RCs relative to HCs. The pathway enrichment analysis calculates the p-value and pathway impact, respectively. The node color is based on the p-value, and the node radius is determined based on pathway impact values.

In addition, 17, 51, and 53 significantly lipids were altered in RM vs. RA, RS vs. RA, and RS vs. RM, respectively (Fig. 5A–C). Overlapping analysis showed that DAG(16:1/18:0) was decreased in RM vs. HC and RS vs. HC (Fig. 5D). Nonetheless, DAG(16:1/18:0) was elevated in the RS vs. RM, whose FC was 62.29 (Table S5 and Fig. 5E).

### Disordered lipid pathways in RCs

We made an enrichment analysis to better understand the implication of significantly altered lipids. Compared to HCs, differential lipids were principally enriched in glycerophospholipid metabolism, glycosylphosphatidylinositol (GPI) anchor biosynthesis, and linoleic acid metabolism (Fig. 5F).

### Associations of altered lipids and clinical indices

**Figure 6:**
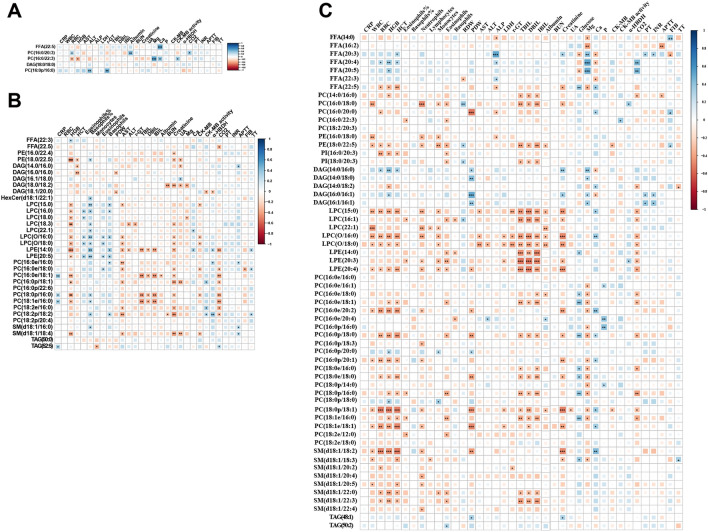
Correlation of plasma sEVs lipids with clinical indices. (**A**–**C**) Correlation plots illustrate Spearman correlations between clinical indices with differential lipids identified in RAs (**A**), RMs (**B**), and RSs (**C**) relative to HCs in 102 recruiters. Only correlation with p < 0.05 was indicated with colored squares and marked with stars (*). Positive and negative correlations were shown in blue and red, respectively, with sizes of squares representing the magnitude of the correlations. *p < 0.05; ** p < 0.01; ***p < 0.001. *RAs* recovered asymptomatic patients, *RMs* recovered moderate patients, *RSs* recovered severe/critical patients.

To evaluate whether altered lipids in RCs were significantly correlated with relevant clinical indices, we performed a Spearman correlation analysis of 102 samples (Fig. 6A–C, Fig. S6, and Tables S6, S7). We observed that the correlations were complicated and most displayed an intimately positive association between differential lipids and clinical indices (Fig. 6C). Of note, not all differential lipids bore a close correlation with clinical indicators in HCs and RCs, which might be ascribed to the fact that the lipid profiles in the RCs were significantly different from those in the HCs, and clinical indices in RCs had returned to normal. For instance, HGB and RBC were negatively associated with 42 and 28 lipids, respectively (Fig. 6A). LPCs, such as elevated LPC(15:0), LPC(O/16:0), and LPC(O/18:0), were dramatically and negatively correlated with r-GT, DBIL, TBIL, and IBIL (Fig. S6B). Creatinine, a renal function indicator, bore significantly and negatively associated with differential lipids, such as LPC(15:0) in the RM vs. HC group (Fig. S6C). PC(18:1e/16:0) was negatively linked with TBIL, DBIL, and CO2 in RM vs. HC group, however, was positively associated with TBIL, DBIL, and CO2 in RS vs. HC group (Fig. S6B,D). As to electrolyte indices, Mg was lowered in M and S patients, closely and negatively linked with PCs in the RS vs. HC group (Fig. S6E).

### Associations of lipids and clinical indices of RCs and HCs

**Figure 7:**
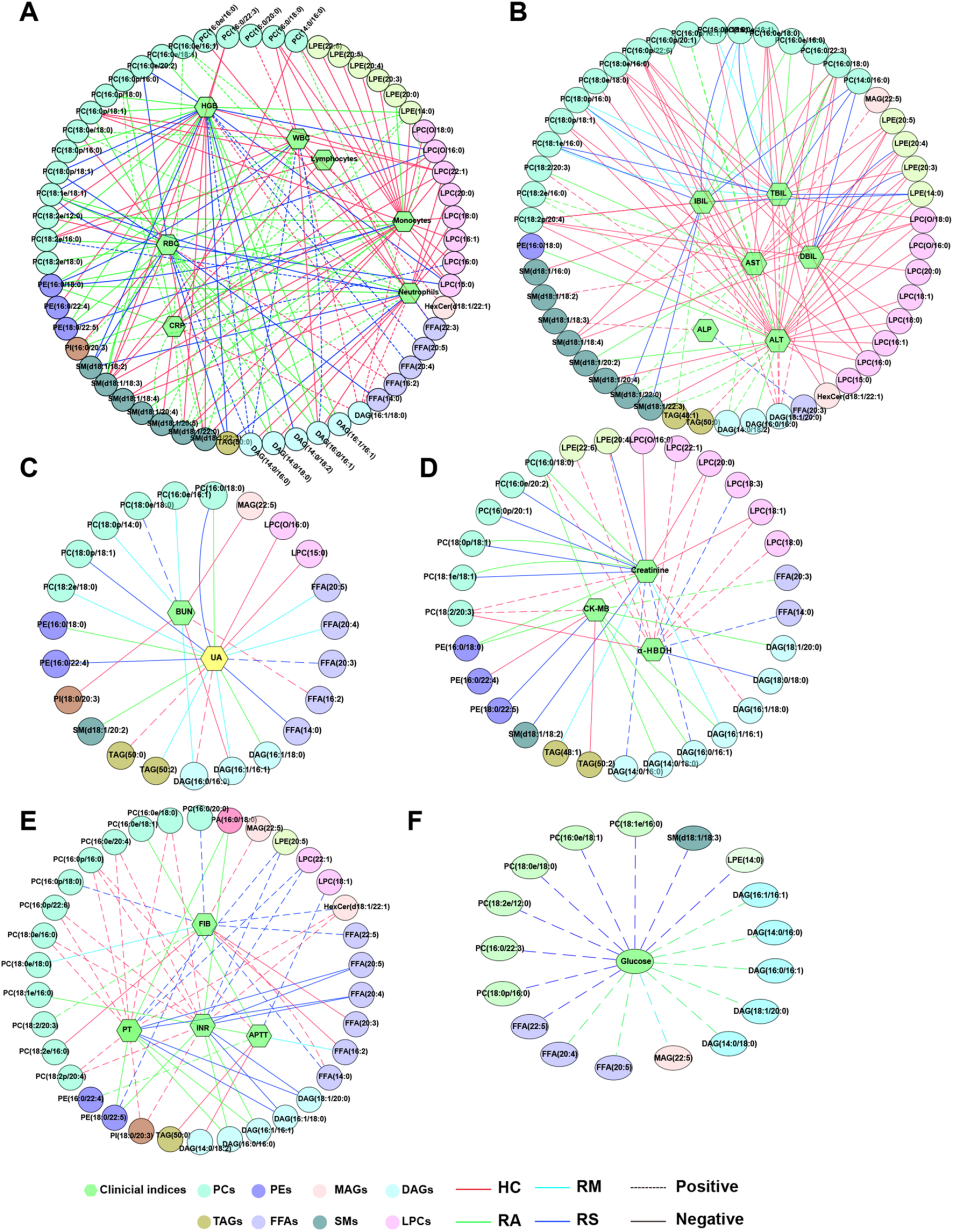
Network of interactions between clinical indices and differential lipids identified in RCs and HCs. Correlation analysis was performed based on the levels of differential lipids and clinical parameters in healthy controls (HCs), recovered asymptomatic patients (RAs), recovered moderate patients (RMs), and recovered severe/critical patients (RSs), respectively. Clinical parameters involved inflammation-related indicators (**A**), hepatic (**B**), renal (**C**), cardiac (**D**), coagulation (**E**) functions, and glucose (**F**). Only correlations with absolute values of correlation coefficients greater than 0.5 and p < 0.05 were left. Correlations between four groups (HCs, RAs, RMs, and RSs) with clinical indices were red, cyan, green, and blue. Continuous and dashed lines indicated negative and positive correlations, respectively.

The correlations between clinical indices and differential lipids in the sEVs of HCs, RAs, RMs, and RSs are presented in Table S8, respectively. Multiple characteristic or unique correlations were found among the four groups. We found that the strong association did not simultaneously exist in the HCs, RAs, RMs, or RSs with the |correlation coefficients|> 0.5 (Fig. 7A). Still, a significant association was detected in HCs and RCs, indicating that the lipids profiles did not revert to normal compared to HCs. For instance, in the RSs, DAG(14:0/16:0) was positively correlated with RBC, HGB, and WBC (Fig. 7A). SM(d18:1/22:0) was positively linked with the CRP, WBC, monocytes, and neutrophils of RAs. PC(16:0p/22:6), PC(18:2p/20:4), PC(18:0e/16:0), LPC(16:0), and LPE(20:5), etc. were strongly and negatively associated with hepatic indices (DBIL, ALT, IBIL, AST, and r-GT) in the HCs (Fig. 7B). Nevertheless, the correlation was not detected in the RCs. Moreover, a strong association was observed in ALP and SM(d18:1/18:3), APTT, and DAG(14:0/18:2), and so forth in the HCs, which was not found in the RCs (Fig. 7B). DAG(16:1/16:1) showed a strong relationship between creatinine and UA (Fig. 7C). In the RMs, DAG(18:1/20:0) displayed a close relationship with PCT, PLT, and α-HBDH. Meanwhile, PE (18:0/22:5) was negatively associated with CK-MB activity (Fig. 7D). Furthermore, a positive association between PC(16:0p/22:6) and coagulation (INR and PT), FFA(22:3), and Mg were found in the HCs but not in the RCs (Fig. 7E and Fig. S7A). Notably, glucose was normal in the RC patients compared to HC but was associated positively with sEVs lipids, especially DAGs, and FFAs (Fig. 7F). These results implied that lipid metabolism in RCs sEVs hasn’t fully recovered to normal.

## Discussion

It is a generally acknowledged fact that sEVs, containing a cell-specific cargo of proteins, lipids, and nucleic acids, could be used as a novel mechanism of intercellular communication and then execute critical biological roles, including immune responses, viral pathogenicity, etc..^13,29,30^. For example, Urbanelli et al. revealed that uninfected cells were infected by viruses using sEVs and Song et al. identified that GM3-enriched sEVs positively linked with disease severity^31,32^. Generally, sEVs are enriched lipids, such as PS, SM, and other FFAs, and bioactive lipids may be internalized into recipient cells, triggering biological responses^33^. Several studies have revealed lipid metabolic abnormalities at the acute phase and fatality patients of COVID-19^31,34^. However, there have been no studies investigating the lipid metabolism of convalescent COVID-19. In the present study, we recruited RCs without underlying diseases 3 months after their discharge and aimed to uncover the lipid alternation in the sEVs plasma of RCs. We extracted sEVs from the plasma of HCs and RCs and confirmed the success of sEVs separation. Then, we used targeted lipidomics to determine the lipid profiles of sEVs. We demonstrated a significant change in lipids of RMs and RSs relative to HCs. Meanwhile, we identified a strong relationship between differential lipids and clinical indices involving blood routine, liver, kidney, heart function, electrolyte, and coagulation.

PCA score plots showed that RA survivors were inseparable from HCs. The result was in keeping with that of Hao Y et al.^35^. Hao Y et al. studied lipidomic alteration in asymptomatic COVID-19 patients at the COVID-19 infection and found various significant lipids in asymptomatic COVID-19 patients compared to HCs^35^. In the present study, only 6 lipids were identified in RAs against HCs. Some differential lipids revealed by Hao Y’s study were not found in our RAs sEVs, such as LPC(22:1) and FFA(18:1). The results indicated that RAs did not differ significantly from HCs in lipid metabolism and displayed strong recovery capability of the human body after COVID-19 infection.

A more dramatic difference was detected in RMs and RSs of sEVs than HCs. The changed lipids subclasses were mainly implicated in PCs, DAGs, SMs, and LPCs. Severe COVID-19 patients were reportedly characterized by an increase in overall TGs and PEs^36^. No significance in TGs was detected in RCs. PCs are the most abundant phospholipid and the major membrane component of mammalian cells and subcellular organelles. It has been revealed that PCs are predominantly metabolized in the liver^37,38^. Previous studies have demonstrated a statistically significant elevation of the levels of PCs after viral infections, such as brome mosaic virus and hepatitis C virus^39,40^. However, we found that the majority of PCs of sEVs exhibited elevated relative to HCs. Furthermore, there has been a report that the macrophage polarization in reaction to viral requires increased absorption of choline for PC formation, thereby promoting inflammatory cytokine secretion^41^. Our previous research revealed a significantly higher level of TNF-α in RCs than HCs^42^. Consistently, we revealed these lipids bore a unique relationship with clinical indicators of organ function, coagulation, and inflammation in the RCs and HCs, suggesting pivotal roles of lipids during these physiological processes. For instance, PC(16:0p/22:6) was significantly and negatively associated with IBIL, DBIL, TBIL, ALT, and AST in the HCs. Hence, it is suspected that sEVs lipids metabolism live has not entirely recovered in RCs after recovery, even though the liver indices were in the normal range.

Plasma sEVs lipids alternations linked with COVID-19 pathogenesis reportedly displayed increased SMs and FFAs and diminished DAGs and TAGs^31^. Interestingly, sEVs’ relative abundance in RCs significantly differed from HCs. We observed a marked reduction of DAGs and an elevation of SMs in RCs, suggesting that sEVs cargo lipids of COVID-19 survivors during convalescence remained aberrant. DAGs and FFAs were observed to have an intimate positive correlation with glucose and are closely associated with insulin resistance^43,44^. Glucose was significantly higher in R and S at the acute stage and returned to normal in recovered COVID-19 survivors, which was consistent with previous studies^44^. And subjects with elevated FFAs are at a higher risk of developing diabetes due to impaired insulin sensitivity^45^. We excluded the potential influence of other clinically vital diseases and acute infection of COVID-19 on lipid metabolism indicating that the glucose was mainly due to COVID-19 infection. Although there has been no glucose disturbance ongoing in recovered COVID-19 patients, whether the organ regulation function of insulin resistance returned to normal remains unknown. Hence, it might be vital for monitoring and estimating the function of glucose regulation and the chance of glucose in the future.

The lung is one of the most vulnerable organs during SARS-CoV-2 infection. In our study, we found that some sEVs FFAs, and LPCs were elevated in RCs relative to HCs. FFAs and LPCs are reported to be associated with the markers of immune activation, such as monocytes and macrophages^46,47^. LPC, a potent proinflammatory mediator, was increased in several types of acute lung injuries^48^. FFAs could enter mitochondria for beta-oxidation to produce ATP for viral replication and transmission, resulting in an impaired antiviral response. In COVID-19 acute patients, a concomitant increase in FFAs, such as FFA(18:1) and FFA(18:2), was observed. In the present study, we found that FFAs (e.g., 22:5 and 16:2) and LPCs were significantly elevated in RSs. Especially, FFA(22:5) was gradually rising with the severity of COVID-19. The results suggested that energy metabolism and immunomodulation of RCs might be abnormal.

Lipids form viral membranes into bioactive substances and act as a critical energy reserve^49^. Guo et al. found that glycerophospholipids metabolism was decreased in COVID-19 patients at the acute stage^50^. Shui et al. revealed that plasma glycerophospholipids, such as PCs, were significantly downregulated during the acute phase of COVID-19^31^. Wu D et al. reported that fatality from COVID-19 could be related to cardiac impairment and showed that the taste transduction pathway is affected^22^. In the current study, we revealed that the differentiating lipids in sEVs of RCs were highly enriched in glycerophospholipid metabolism. Hence, These results suspected that metabolic abnormalities may persist in recovered COVID-19.

This study has several limitations. First, we assembled case information of HCs and RCs but did not collect clinical data on the symptoms of RCs due to the potential restraints during the early outbreak. Second, we determined the lipid profiles of sEVs separated from RCs with different severities and examined the correlation between differential lipids and clinical indicators. However, the exact mechanism underlying such an association was further investigated. Third, our targeted lipidomics detection and results were based on a single cohort of Chinese recovered from COVID-19. Hence, additional studies should be conducted in different racial populations and geographical regions.

## Conclusion

Our study systematically examined the lipidomic profiles of sEVs extracted from the plasma of RCs with different severities, ranging from asymptomatic carriers to severe or critical patients. We uncovered that convalescent COVID-19 3 months after discharge still experienced prominent lipid abnormality, especially in RS survivors. Pathway analysis showed that these changing lipids were involved in glycerophospholipid metabolism glycosylphosphatidylinositol anchor biosynthesis. Of particular importance, we revealed differential lipids linked with organ injuries in clinical practice.

## Materials and methods

### Reagents

HPLC-grade acetonitrile (ACN), chloroform, methanol, ammonium acetate, and isopropanol were ordered from Thermo Fisher Scientific (Rockford, USA). Ultrapure water was obtained by a Millipore Reference system (Millipore, MA, USA). Internal standards were purchased from Sigma (Sigma-Aldrich, Shanghai, USA), including 15:0–18:1_(d7)_PA (7 µg/mL); 15:0–18:1_(d7)_PE (5 µg/mL); 15:0–18:1_(d7)_PG (30 µg/mL); 15:0–18:1_(d7)_PI (10 µg/mL); 15:0–18:1_(d7)_PS (5 µg/mL); 15:0–18:1 (d7) PC(160 µg/mL); 15:0–18:1 (d7) DG (10 µg/mL); 15:0–18:1 (d7)–15:0 TG (55 µg/mL); 18:1 (d7) CE (350 µg/mL); 18:1 (d7) LPC(5 µg/mL); 18:1 (d7) LPE (5 µg/mL); 18:1 (d7) MG (2 µg/mL); 18:1 (d9) SM(30 µg/mL); Cholesterol (d7, 100 µg/mL).

### Study design and participants

We enrolled 102 candidates to conduct a prospective study from Wuhan Union Hospital, Tongji Medical College, Huazhong University of Science and Technology, Wuhan, China. These candidates consisted of 83 RCs without underlying diseases 3 months after discharge and 19 HCs. Age, sex, and body mass index (BMI) were matched between RCs and HCs. RCs were split into three groups by disease severity at the infection phase, including RAs (n = 18), RMs (n = 32), and RSs (n = 33) based on COVID-19 clinical guidelines of the New Coronavirus Pneumonia Prevention and Control Program (the 7th edition). Recruited RAs were from the health check-up department and HCs came from the health examination center of Wuhan Union Hospital.

This study was approved by the Ethics Committee of Wuhan Union Hospital (0271) and performed following the Declaration of Helsinki, which was registered on the trial registration websites (NCT04283396). Informed written consent was obtained from all subjects before participation. All recruiters underwent blood tests, including complete blood count, coagulation index, renal and hepatic function, and creatine kinase.

### Plasma collection

Blood samples from RCs and HCs, collected with K2EDTA anticoagulant tubes, were centrifuged at 1500 rpm for 20 min to remove cells and debris. Then, the supernatant was transferred to a new tube and centrifuged again at 3000×*g* for 15 min at 4 °C. Lastly, the supernatant was stored at − 80 °C for later use.

### Cell culture

Human lung adenocarcinoma cell line A549 was purchased from Shanghai Institutes for Biological Science, China, and maintained in RPMI 1640 medium containing 10% fetal bovine serum and 1% penicillin–streptomycin (Beyotime, C0222). And cells were cultured in a 37 °C humidified incubator with 5% CO2.

### Isolation of sEVs

Schematic diagram of the sEVs separation is shown in Fig. 1A. Briefly, the plasma samples were centrifugated at 10,000×*g* at room temperature (RT) to entirely pellet debris. PBS was added to the supernatant, and mixtures were vortexed thoroughly. Later, sEVs precipitation reagent was added to the mixtures, vortexed thoroughly, and put at RT for 10 min. Lastly, the samples were fractionated by centrifugation at 10,000×*g* for 5 min at RT to obtain the supernatant and precipitation. The precipitation was resuspended in 200 μL PBS and sEVs were stored at − 80 °C for lipidomic extraction.

### TEM

TEM characterized the morphology of sEVs. Briefly, 10 μL purified sEVs were deposited onto 200 carbon grids for 1 min. After that, the excess liquid was removed with filter papers. The grids were negatively dyed with uranyl acetate for 1 min at RT and photographed under an HT-7700 transmission electron microscope (Hitachi Ltd., Tokyo, Japan) at 100 kV accelerating voltage.

### Nanoparticle tracking analysis

Nanoparticle tracking analysis (NTA) was performed to determine the size and particle concentration of sEVs using a Nanosight NS300 (Malvern Instruments, Malvern, UK), equipped with a laser blue 488. Particle numbers were analyzed with the Nanosight software (NTA 3.4 Build 3.4.003) with the following capture parameters: camera level: 15, slider shutter: 1206, shutter/ms: 30.15, slider gain: 366, FPS: 25.0, number of frames: 749, and syringe pump speed: 30.

### Nanoflow analysis

According to the manufacturer’s instructions, the High-Sensitivity Flow Cytometry (NanoFCM Inc. China) was employed to determine the particle size distribution of the sEVs ^51^. The capture settings were set: sample pressure: 1.0 Kpa, SS decay: 10%, and min-width: 0.3 ms. Data analysis was performed using the NanoFCM Professional Suite V1.08 software.

### Western blotting

The purified sEVs and A549 cells were lysed with 1 × RIPA buffer and cocktails and centrifuged at 13,000×g for 15 min to obtain the supernatant. Protein concentrations were detected with BCA Protein Assay according to the manufacturer’s protocols. sEVs markers CD9 (Abcam, ab92726), CD63 (Abcam, ab193349), TSG101 (Abcam, ab83), and sEVs non-markers calnexin (Abcam, ab22595), GM130 (Abcam, ab52649), Lamin B1 (Abcam, ab16048), and GRP94 (Proteintech, 14700-1-AP) were measured to validate the expressions of sEVs as described in prior studies^52,53^.

### Lipid extraction and ultra-performance liquid chromatography triple quadrupole mass spectrometry (UPLC-TQMS) analysis

The lipidomics of sEVs samples was performed by Metabo-Profile (Shanghai, China). The sample preparation procedures were according to the previously published methods with minor modifications^54,55^. Briefly, 95 μL of sEVs was extracted with 500 μL chloroform: methanol (1:2) (v/v) with 5 μL internal standards. Then, to utter precipitation and lipids extraction, the mixture was vortexed for 10 min, incubated for 10 min at − 20 °C, stand at room temperature for 10 min and 4 °C for 2 h, and centrifuged at 18,000×g for 10 min at 4 °C. Then, the lower fraction was transferred to a new tube and lyophilized to dryness in a vacuum. The dried lipids were dissolved in 100 μL isopropanol, vortexed for 10 min, and centrifuged at 10,300×g for 10 min at 4 °C. The supernatant was transferred to UPLC-TQMS (ACQUITY UPLC-Xevo TQ-S, Waters Corp., Milford, MA, USA) equipped with BEH Amide 1.7 µM analytical column (2.1 × 100 mm, Waters Corp., Milford, MA, USA) for lipids detection. The gradient was started at a flow rate of 0.6 mL/min, with the injected volume being 1 µL and the column temperature at 10 °C. Both mobile phases A and B contained 5 mM ammonium acetate in a solution of A: ACN/H2O (95:5, v/v) and B: ACN/IPA (50:50, v/v), respectively. The lipids were eluted using the following gradients: 0–2 min, 0.1–20% B; 2–5 min, 20–80% B; 5–5.1 min, 80–0.1% B; and 5.1–8 min, 0.1% B for both modes. The Capillary (Kv) of the ESI+ and ESI- modes was set at 2.8 and 1.9, respectively. Desolvation gas flow (L/H) and collision gas flow (L/H) were set at 1000 and 0.13, respectively. And the electrospray ion source temperature and desolvation temperature were maintained at 120 °C and 500 °C, respectively. In addition, to estimate the repeatability and stability of the LC–MS system, the quality control (QC) samples were prepared by mixing 10 μL aliquots from each sample. One QC sample was inserted into every ten samples and was injected intermittently during the entire sample injection process to eliminate bias induced by the sequence order of samples^56^.

### Data processing and statistical analysis

Peak integration, calibration, and quantitation of raw data of lipids generated by UPLC-TQMS were performed using the MassLynx software (v4.1, Waters, Milford, MA, USA). After removing missing values through the rule of 80% and background ions, the signal drifts of the rest matched peak ions were calibrated via total intensity signal calibration. The signal intensities and peak areas for different lipid species were extracted based on their retention time, lipid intensity was normalized to the representative spiked internal standards. Principal component analysis (PCA) was used to conduct a preliminary investigation of the pre-processed data. Then, The orthogonal partial least square discriminant analysis (OPLS-DA) was undertaken to distinguish RCs from HCs using SIMCA-P software (version 14.1, Umetrics). Univariate analysis was conducted using Multi Experiment Viewer software (MeV, version 4.7.4). The Metaboanalyst tool (https://www.metaboanalyst.ca/) was used to identify high-enriched pathways of differential lipids. The correlation analysis between differential lipids and clinical indices was conducted using SPSS software (25.0.0). The network was displayed with Cytoscape software (v3.4.0).

Comparisons were made regarding continuous variables using the one-way ANOVA for variables with a normal distribution, the Kruskal–Wallis test for variables with a non-normal distribution, and the chi-square test or Fisher’s exact test for category variables. Data were considered statistically significant with p < 0.05. Statistical analyses were performed using SPSS software (25.0.0) and data were graphed using GraphPad Prism (8.02).

### Ethics declarations

This study was approved by the Ethics Committee of Wuhan Union Hospital (0271) and performed following the Declaration of Helsinki, which was registered on the trial registration websites (NCT04283396).

### Supplementary Information

Supplementary Information 1.

Supplementary Information 2.

## Data Availability

All data that support the finding of this study generated and analyzed for the current study are available from the corresponding author upon request.
