# Supplementary material for: Lipid metabolism of plasma-derived small extracellular vesicles in COVID-19 convalescent patients
Source: Sci Rep. 2023 Oct 3;13:16642. doi: 10.1038/s41598-023-43189-5 (PMC10547714; doi:10.1038/s41598-023-43189-5)
Supplement: Supplementary file 1 — Supplementary Information 1. [file 41598_2023_43189_MOESM1_ESM.pdf]

Lipid metabolism of plasma-derived small extracellular vesicles in COVID-19 convalescent patients

Wenjing Xiao[a,b]†, Qi Huang[a,b]†, Ping Luo[c]†, Xueyun Tan[a], Hui Xia[a], Sufei Wang[a], Yice Sun[a], Zhihui Wang[d], Yanling Ma[a], Jianchu Zhang[a,b]*, Yang Jin[a,b,e]*

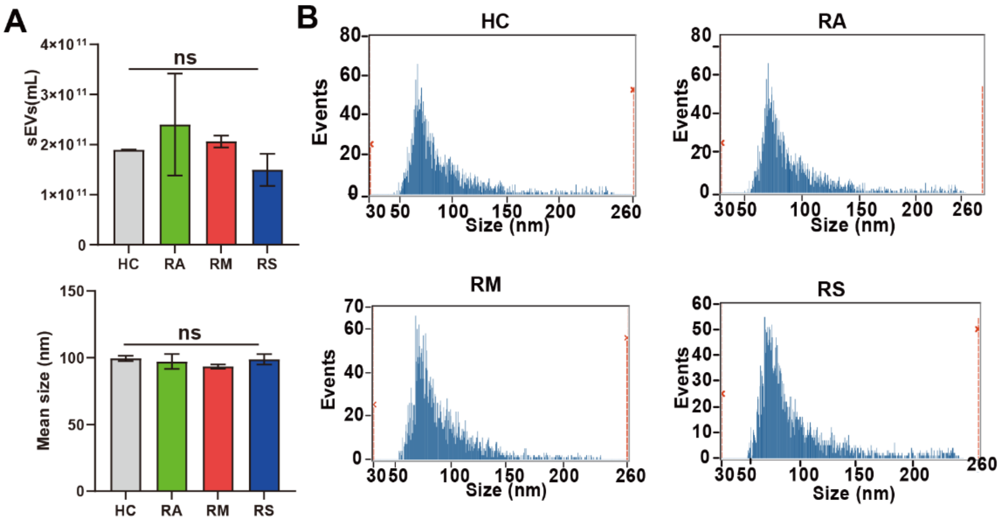

Figure S1. (A) Average size comparison and concentration of small extracellular vesicles (sEVs) from the plasma of recovered asymptomatic patients (RAs, n=18), recovered moderate patients (RMs, n=32), and recovered severe and critical patients (RSs, n=33), and healthy controls (HCs, n=19). **(B)** The size distribution of the purified sEVs isolated from plasma of HCs, RAs, RMs, and RSs as revealed by Flow Nano Analyzer.

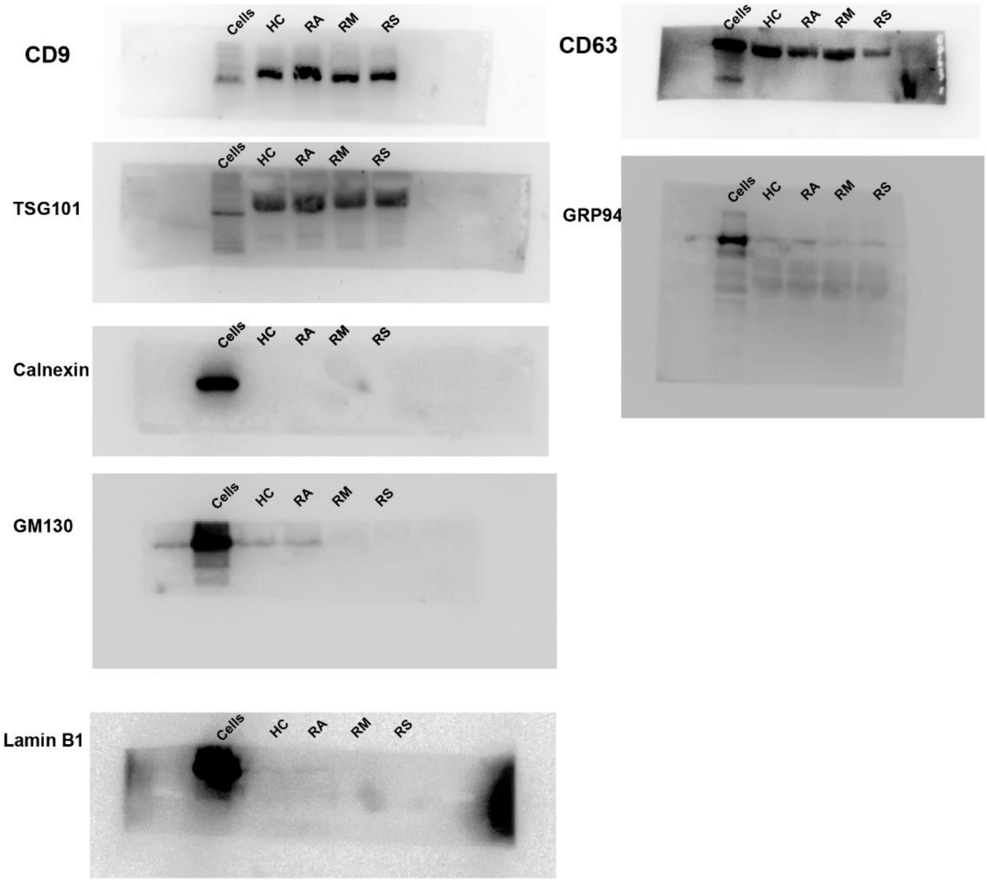

Figure S2. Original blots with membrane edges visible of Figure 1E.

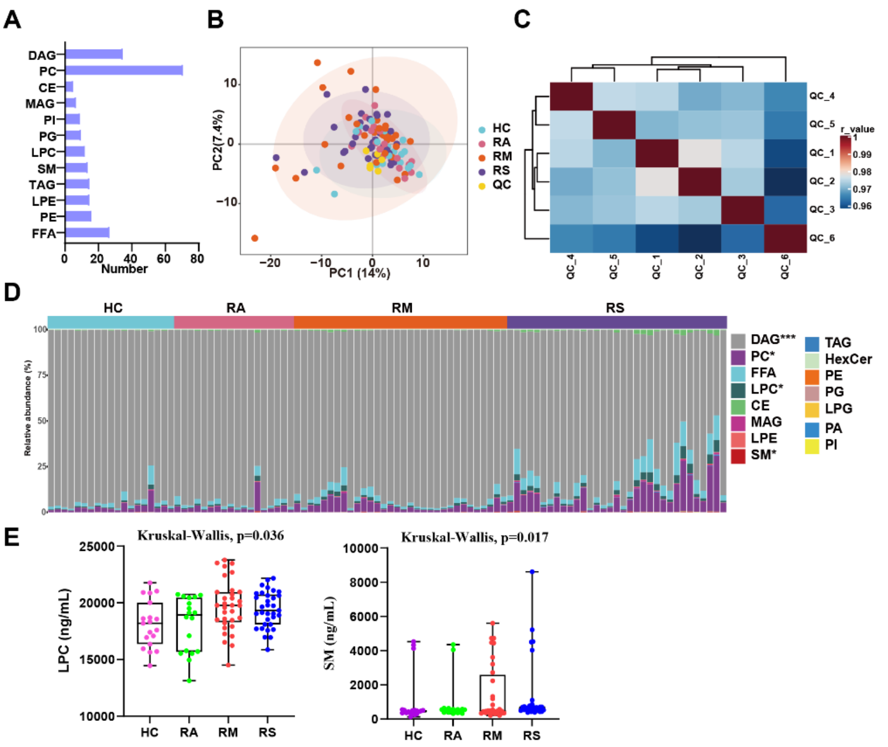

Figure S3. (**A**) The number of identified lipids subclasses. **(B)** The PCA score plot shows the clustering of quality control (QC) samples from recovered asymptomatic patients (RAs), recovered moderate patients (RMs), and recovered severe and critical patients (RSs), and healthy controls (HCs). (**C**) The correlation coefficient of QC samples was indicated with Pearson correlation. (**D**) This graph represents the average percentage of lipids subclasses of each sample from HCs, RAs, RMs, and RSs. (**E**) lipids subclasses lysophosphatidylcholines and sphingomyelins (SMs) in RAs, RMs, RSs, and HCs. Data were displayed as boxplots with median and range and each dot represents an individual: HC (Pink), RA (Green), RM (Red), RS (Blue).

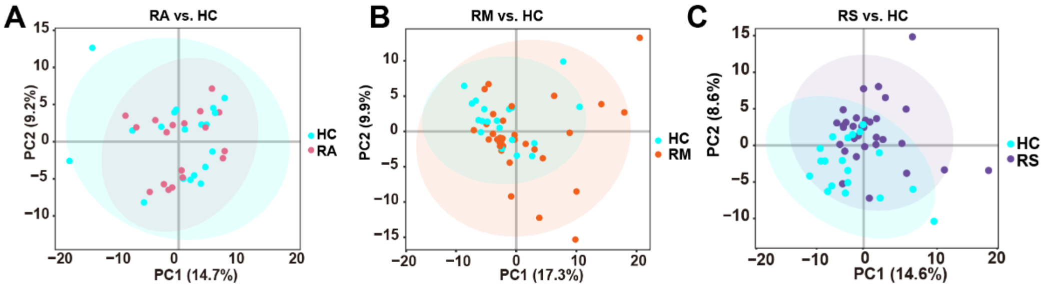

Figure S4. (A–C) PCA score plots of lipid profiles detected in plasma sEVs in RAs (**A**), RMs (**B**), and RCs (**C**) compared with HCs, respectively. p-value: *p<0.05; **p<0.01; ***p<0.001.

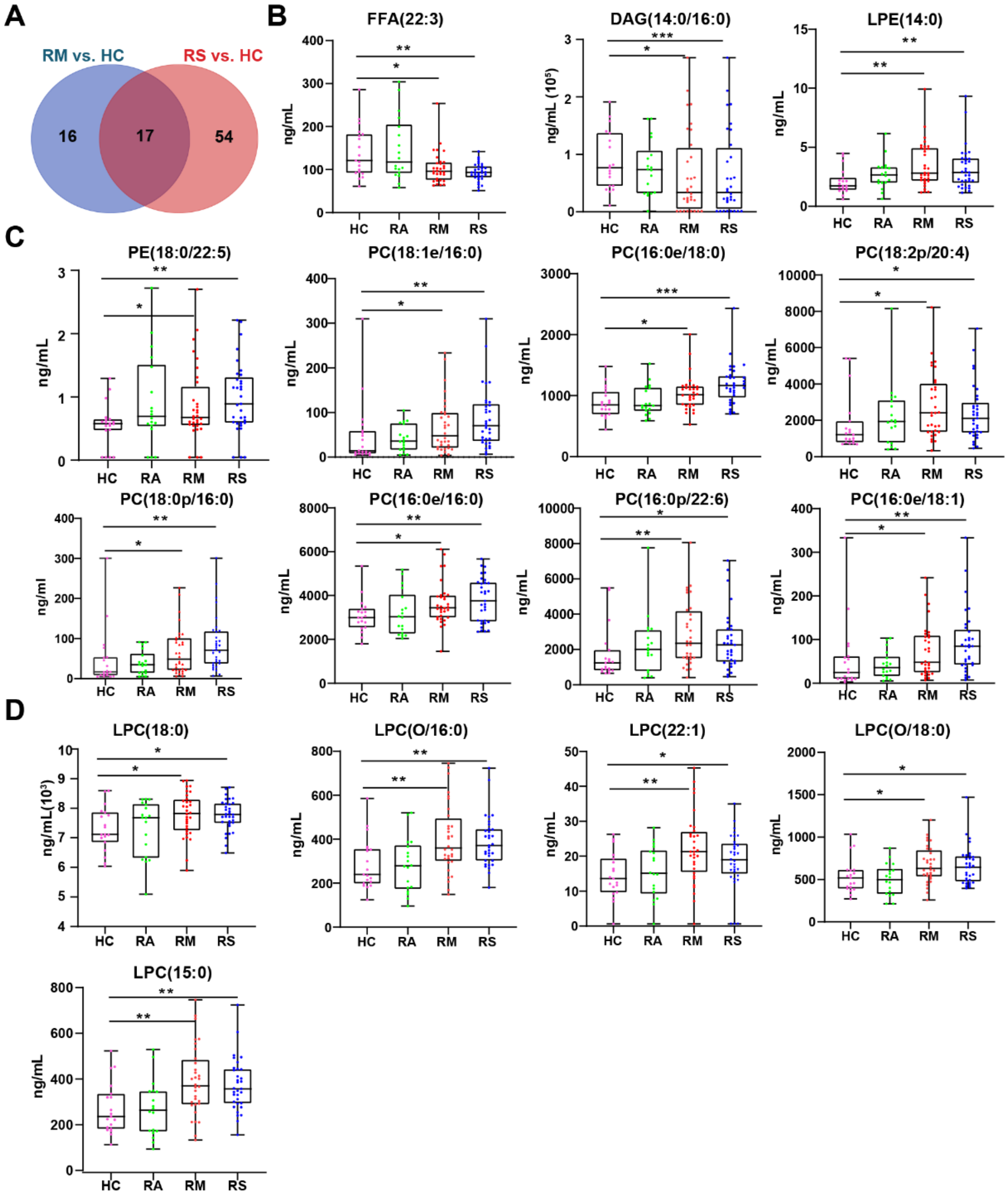

Figure S5. Significantly altered lipids in RM and RS compared to HCs. **(A)** Venn diagram showing the number of significantly changed lipids in recovered moderate patients (RMs) and recovered severe and critical patients (RSs) relative to healthy controls (HCs). **(B–D)** The significance of comparisons of the four groups was determined by the Kruskal-Wallis test (data with non-normal distribution) or one-way ANOVA (data with normal distribution) and indicated as an absolute overall p-value. *$p<0.05$; **$p<0.01$; ***$p<0.001$.

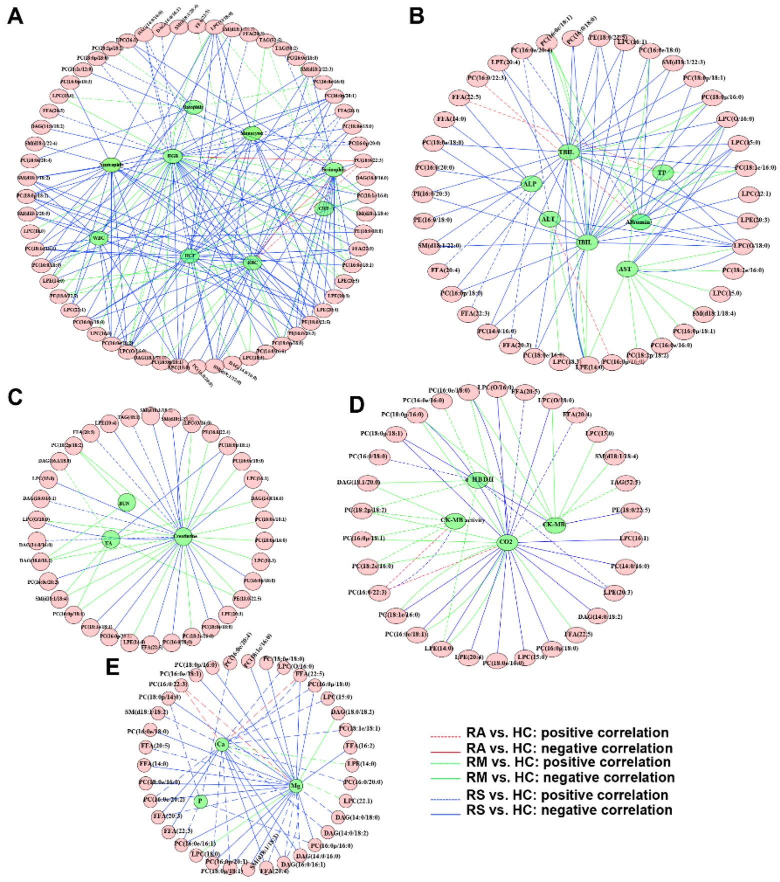

Figure S6. Network interactions between clinical indices and differential lipids identified in RAs, RMs, and RSs relative to HCs. Correlation analysis was performed based on the levels of differential lipids identified from recovered asymptomatic patients (RAs), recovered moderate patients (RMs), and recovered severe and critical patients (RSs) and clinical parameters, including inflammation-related indicators (**A**), hepatic function (**B**), renal function (**C**), cardiac function (**D**), and electrolyte (**E**). Only correlations with correlation coefficients |R| greater than 0.4 and $p<0.05$ were left. Red, green, and blue represent RA vs. HC, RM vs. HC, and RS vs. HC groups. Continuous and dashed lines indicated negative or positive correlation, respectively.

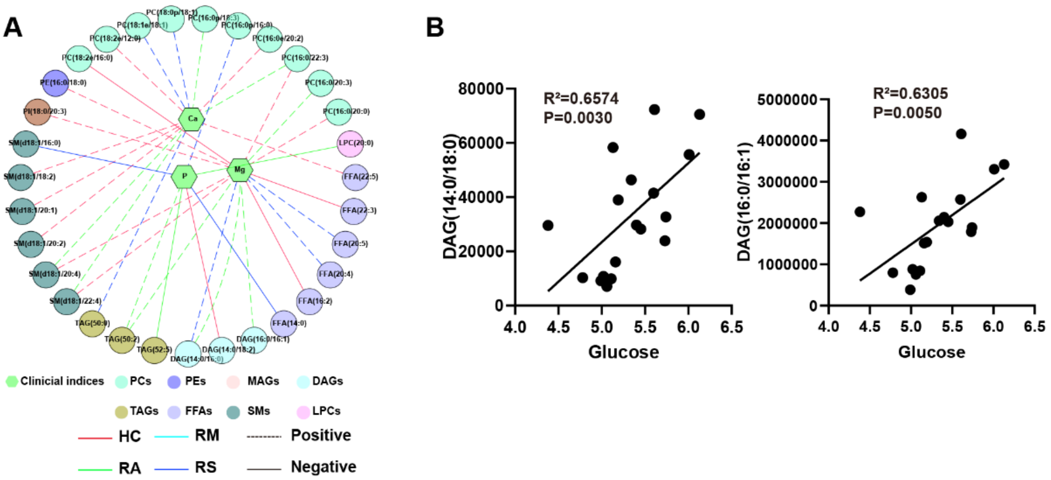

Figure S7. Network of interactions between electrolyte equilibrium and differential lipids identified in RCs and HCs (A). Only correlations with absolute values of correlation coefficients greater than 0.5 and $p<0.05$ were left. Correlations between four groups (HCs, RAs, RMs, and RSs) with clinical indices were red, cyan, green, and blue. Continuous and dashed lines indicated negative and positive correlations, respectively. (B) The correlation of DAG (14:0/18:0) and DAG(16:0/16:1) with glucose in RA sEVs. Abbreviation: and. CE, Cholesteryl ester; Cer, Ceramide; DAG, Diacylglycerol; FFA, free fatty acid; HexCer, Hexosylceramide; LPA, lysoPhosphatidic acid; LPC, lysoPhosphatidylcholine; LPE, lysoPhosphatidylethanolamine; MAG, Monoacylglycerol; PA, phosphatidic acid; PC, phosphatidylcholine; PE, phosphatidylethanolamine; PG, Phosphatidylglycerol; recovered asymptomatic patients, RAs; RMs, recovered moderate patients; RSs, recovered severe and critical patients; SM, sphingomyelin; TAG, triacylglycerol.

Table S1. Clinical characteristics of RCs without underlying diseases 3 months after discharge

Variables	HC (n=19)	COVID-19 patients on admission		COVID-19 patients 3 months after discharge		
		M (n=32)	S (n=33)	RA (n=18)	RM (n=32)	RS (n=33)
**Characteristics**						
**Age, years**	45 (38–54)	45 (38.25–52)	46 (36–54.5)	46.5 (38–55.25)	45 (38.25–52)	46 (36–54.5)
**Sex**						
**Female**	8 (42.10%)	19 (59.40%)	19 (57.60%)	7 (38.90%)	19 (59.40%)	19 (57.60%)
**Male**	11 (57.90%)	13 (40.60%)	14 (42.40%)	11 (61.10%)	13 (40.60%)	14 (42.40%)
**BMI, kg/m^2**	23.6 (21.67–25.6)	22.77 (20.99–25.06)	23.6 (22.17–26.83)	24.55 (23.33–25.73)	22.77 (20.99–25.06)	23.6 (22.17–26.83)
**Laboratory findings**						
**SARS-CoV-2 nucleic acid test (+)**	0 (0.00)	0 (0.00)	0 (0.00)	0 (0.00)	0 (0.00)	0 (0.00)
**IgG (+)**	0 (0.00)	–	–	16 (88.90%)	29 (96.7%)	30 (96.8%)
**IgM (+)**	0 (0.00)	–	–	2 (11.1%)	5 (15.6%)	5 (16.1%)
**CRP, mg/L**	0.76 (0.44–2.68)	3.8 (0.1–7.85) [a]	7.71 (2.42–27.97) [a]	0.69 (0.08–1.39)	0.41 (0.13–1.25) [b]	0.74 (0.38–1.42) [c]
**WBC, × 10^9 per L**	5.55 (4.74–6.3)	5.57 (4.25–6.86)	5.07 (3.96–6.48)	5.25 (4.89–5.81)	4.89 (4.32–6.1)	5.12 (4–5.6)
**RBC, × 10^{12} per L**	4.87 (4.54–5.3)	4.15 (3.9–4.5) [a]	4.35 (3.73–4.79) [a]	4.9 (4.42–5.36)	4.65 (4.34–5.06) [b]	4.72 (4.36–5.26) [c]
**Haemoglobin, g/L**	155 (143.5–164)	128 (120–137) [a]	130 (119–147) [a]	151 (137.25–162)	142 (140–157) [b]	144 (136–160.5) [c]
**Hematocrit, %**	45.3 (42.2–48.5)	38.1 (36.23–41.05) [a]	38.8 (34.85–43.6) [a]	44.95 (42–47.83)	43.95 (40.53–48.53) [b]	43.8 (41.2–47.75) [c]
**MCV, fl**	93.1 (89.9–96.35)	91.55 (88.93–96.63)	92.1 (89.9–93.8)	93.05 (89.35–96.2)	92.45 (89.18–95.38)	92.8 (90.3–94.45)
**MCH, pg**	31.3 (30.2–32.75)	30.75 (29.8–32.23)	30.9 (30–31.65)	31.4 (29.8–32.78)	30.85 (30–32)	30.9 (30.25–31.75)
**MCHC, g/L**	338 (334.5–343)	333 (326.75–336.25) [a]	334 (331–338) [a]	339.5 (332.75–345.25)	336.5 (330.5–339.75)	335 (330.5–339.5)
**Platelets, ×10^9 per L**	219 (182.5–262.5)	205 (175–257)	228 (164.5–266.5)	194 (184–221)	212 (187.5–236.75)	216 (182.5–234)
**Percentage of**	34.3 (27.8–39.2)	29.65 (22.33–37.93)	26.6 (18.25–33.65) [a]	27.95 (25.48–37.9)	35.05 (29.55–40.98)	34 (28.4–40.5) [c]

**lymphocytes, %**						
**Percentage of monocytes, %**	4.8 (4.45–5.2)	6.75 (6.1–8.73) [a]	7.7 (4.75–9.7) [a]	5.3 (4.43–6.1)	5.3 (4.83–6.5) [ab]	5.2 (4.45–5.9) [c]
**Percentage of eosinophils, %**	2.5 (1.15–4.3)	1.85 (1.18–3.68)	0.7 (0.1–1.85) [a]	1.4 (0.98–1.95)	1.85 (1.3–2.73)	1.8 (1.25–3.3) [c]
**Percentage of basophils, %**	0.1 (0.1–0.25)	0.35 (0.2–0.5) [a]	0.3 (0.2–0.5) [a]	0.1 (0.1–0.23)	0.2 (0.1–0.3)	0.2 (0.05–0.3)
**Neutrophils, $\times 10^9$ per L**	3.15 (2.78–3.6)	3.35 (2.33–4.6)	3.17 (2.17–4.48) [a]	3.29 (2.74–3.59)	2.7 (2.31–3.55)	2.92 (2.3–3.15)
**Lymphocytes, $\times 10^9$ per L**	1.83 (1.43–2.45)	1.61 (1.33–2.04)	1.35 (0.89–1.77) [a]	1.56 (1.25–2.04)	1.77 (1.48–2.09)	1.6 (1.35–2.05)
**Monocytes, $\times 10^9$ per L**	0.26 (0.22–0.3)	0.37 (0.29–0.46) [a]	0.37 (0.28–0.43) [a]	0.3 (0.23–0.31)	0.29 (0.23–0.35) [b]	0.26 (0.19–0.32) [c]
**Eosinophils, $\times 10^9$ per L**	0.13 (0.08–0.21)	0.09 (0.04–0.2)	0.04 (0.01–0.1) [a]	0.07 (0.05–0.1)	0.1 (0.06–0.15)	0.1 (0.06–0.15) [c]
**Basophils, $\times 10^9$ per L**	0.01 (0–0.01)	0.02 (0.01–0.03) [a]	0.01 (0.01–0.03) [a]	0.01 (0–0.01)	0.01 (0–0.02)	0.01 (0–0.01) [c]
**PCT, %**	0.21 (0.19–0.24)	0.21 (0.18–0.24)	0.22 (0.17–0.26)	0.2 (0.18–0.22)	0.21 (0.19–0.23)	0.2 (0.18–0.23)
**AST, U/L**	23 (20.5–26)	26 (18–34) [a]	26 (20–45.5) [a]	25 (22–31.25)	20.5 (18–24)	23 (20–28)
**ALT, U/L**	22 (16.5–31.5)	28 (22–39) [a]	30 (17–48.5) [a]	26 (16.75–59.5)	19.5 (14–28.5)	22 (18–43)
**AST/ALT**	1 (0.76–1.28)	0.85 (0.56–1.12)	1.07 (0.7–1.25)	1 (0.55–1.4)	1.07 (0.73–1.19)	0.95 (0.69–1.2)
**ALP, U/L**	68 (56–86.5)	50 (42–63) [a]	52 (43–70.5) [a]	54.5 (48.75–76.75)	57.5 (47.5–68.75) [a]	63 (49.5–72)
**γ-GT, U/L**	20 (16–26)	23 (14–36)	25 (16.5–42)	24 (16.75–66.5)	19 (12–24)	19 (13.5–29.5)
**TBIL, µmol /L**	13.2 (10.75–18.9)	9.5 (7.4–10.3) [a]	8.8 (7.5–11.45) [a]	14.95 (13.1–18.25)	12.1 (9.6–15) [b]	13 (10.85–15.15) [c]
**DBIL, µmol/L**	3 (2.3–4.8)	2.5 (1.8–2.9)	3 (2.1–3.5)	3.55 (2.8–4.68)	3.2 (2.63–4.28) [b]	3.1 (2.4–4.25)
**IBIL, µmol/L**	10 (8.3–14.05)	6.9 (5.9–8.2) [a]	6.1 (5.2–7.6) [a]	11.7 (10.05–13.98)	8.8 (6.93–11.33)	9.7 (8.1–12.1) [c]

**Total protein, g/L**	77 (73.45–79.85)	66.8 (63.8–70.8)[a]	64.6 (59.1–69.3)[a]	78.35 (76.78–81.8)	76.45 (73.33–79.08)[b]	76.8 (73.8–79.45)[c]
**Albumin, g/L**	47.7 (46.35–48.3)	40.1 (35.7–42.4)[a]	33.9 (30.3–39.05)[a]	47.9 (45.88–48.98)	46.8 (44.45–49.38)[b]	46.9 (45.6–50)[c]
**Globin, g/L**	29.4 (26.7–31.65)	29.1 (26.3–32.8)	29.15 (25.6–31.53)	30.9 (29.58–33.78)[a]	29.35 (26.85–31.78)[b]	29.6 (27.9–31.55)
**Albumin/globin**	1.6 (1.5–1.75)	1.3 (1–1.5)[a]	1.35 (1.13–1.48)[a]	1.5 (1.4–1.6)	1.6 (1.43–1.78)[b]	1.6 (1.5–1.7)[c]
**BUN, mmol/L**	5.39 (5.01–5.81)	3.69 (3.15–4.9)[a]	4.03 (3.11–5.08)[a]	4.68 (4.19–5.42)[a]	4.34 (3.9–5.29)[a]	4.77 (4.05–5.73)
**Creatinine, μmol/L**	71.8 (63.8–78.85)	60.2 (52.18–71.55)[a]	62.65 (50.58–81.6)	74.8 (59.1–82.13)	61.95 (55.9–77.33)	61.4 (56–74.8)
**UA, μmol/L**	349.4 (283.75–405.3)	264.3 (202.95–312.23)[a]	283.35 (205.8–341.58)[a]	378.55 (286.23–457.68)	298.35 (247.28–400.18)	368.1 (306.75–429.9)[c]
**Glucose, mmol/L**	5.15 (5–5.28)	5.6 (5.05–6.65)[a]	5.3 (5.03–5.9)	5.27 (5.05–5.64)	5.11 (4.91–5.49)	5.36 (5–5.61)[c]
**Mg, mmol/L**	0.89 (0.88–0.94)	0.85 (0.78–0.89)[a]	0.84 (0.79–0.9)[a]	0.92 (0.89–0.95)	0.87 (0.83–0.89)[a]	0.86 (0.83–0.88)[a]
**Ca, mmol/L**	0.92 (0.75–1.05)	1.04 (0.94–1.29)[a]	1.2 (1.04–1.37)[a]	0.99 (0.88–1.08)	0.99 (0.93–1.09)	0.98 (0.86–1.11)[c]
**P, mmol/L**	2.18 (2.15–2.27)	2.04 (1.92–2.15)[a]	2.06 (1.98–2.18)[a]	2.2 (2.16–2.3)	2.23 (2.16–2.31)[b]	2.2 (2.16–2.26)[ac]
**Creatine kinase, U/L**	98 (82.5–151.5)	73 (62–106)[a]	63 (44–117.5)[a]	105 (82.25–145.75)	92 (71–118)	108 (74–121.5)
**LDH, U/L**	188 (156–218)	179 (146–211)	230 (184.5–322.5)[ad]	185.5 (167.75–205.75)	176.5 (155.5–189.25)	177 (165–201.5)[c]
**CK-MB activity, U/L**	10 (8–13)	11 (8–16)	10 (8–12)	9.5 (8–12)	12 (9–15)	13 (11–14.5)
**α-HBDH, U/L**	141 (128–163)	171 (124–283)[a]	155 (122–175)[a]	135 (102.75–140.25)[a]	136 (122.5–167.5)	145 (126.5–157)
**CO_2, mmol/L**	27.6 (26.3–28.6)	27.2 (23.15–30.93)	26.8 (22.4–28.8)	24.35 (21.4–25.05)[a]	24.1 (22.73–25.9)[ab]	25.2 (22.9–27.3)[a]
**PT, s**	12.2 (11.6–12.8)	12.6 (12.3–13.5)[a]	12.9 (12.5–13.2)[a]	12.1 (11.55–12.38)	12.3 (11.8–12.95)	11.7 (11.5–12.15)[ac]
**INR**	0.92 (0.86–0.98)	0.96 (0.93–1.05)[a]	0.99 (0.95–1.03)[a]	0.91 (0.86–0.94)	0.93 (0.88–1)	0.87 (0.85–0.92)[ac]
**APTT, s**	38 (36.3–40.6)	35.7 (33.7–40.5)	35.3 (32.15–38.25)[a]	36.95 (34.78–39.23)	36.55 (33.68–39.35)	35.4 (33.55–37.45)[a]
**FIB, g/L**	3.08 (2.82–3.22)	3.68 (3.08–4.4)[a]	3.48 (3.14–4.34)[a]	3.02 (2.72–3.3)	2.99 (2.73–3.33)[b]	2.98 (2.82–3.22)[c]
**TT, s**	16.2 (15.9–16.6)	15.1 (14.6–16.5)[a]	15.3 (14.95–15.65)[a]	15.9 (15.35–16.8)	16.15 (15.53–16.9)[b]	16.1 (15.65–16.45)

Abbreviation:

ALP, alkaline phosphatase; ALT, alanine aminotransferase; APTT, activated partial thromboplastin time; AST, aspartate aminotransferase; BUN, blood urea nitrogen; Ca, calcium; CK-MB, creatine kinase-myocardial band; CRP, C-reactive protein; DBIL, direct bilirubin; FIB, fibrinogen; HCs, healthy controls; IBIL, indirect bilirubin; INR, international normalized ratio; LDH, lactate dehydrogenase; M, moderate; MCH, mean corpuscular hemoglobin; MCHC, mean corpuscular hemoglobin concentration; MCV, mean corpuscular volume; Mg, magnesium; P, phosphorus; PCT, plateletocrit; PDW, platelet distribution width; PT, prothrombin time; RAs, recovered asymptomatic patients; RBC, red blood cell; RDW, red blood cell volume distribution width; RMs, recovered moderate patients; RSs, recovered critical and severe patients; S, critical and severe; TBIL, total bilirubin; TT, thrombin time; UA, urine acid; WBC, white blood cell; α-HBDH, α-hydroxybutyrate dehydrogenase; γ-GT, γ-glutamyl transpeptidase;

Data were shown as median (interquartile range, IQR) or n (%).

Comparisons were made regarding continuous variables between two groups using the *t*-test for variables with a normal distribution and the Wilcoxon rank-sum test for variables with a non-normal distribution.

[a]$p<0.05$, HC vs. M, S, RA, RM, and RS; [b]$p<0.05$, comparison between M and RM, [c]$p<0.05$; comparison between S and RS.

Table S2. Particle concentration and size (mode and mean) of plasma-derived sEVs

Groups	Particle concerntration (10^{10})	Mean size（nm）	Mode size（nm）	Size range D10 and D90（nm）
HC sEVs	18.97±0.03	99.53±1.12	65.30±3.42	60.33–152.07
RA sEVs	25.00±5.30	97.20±3.22	63.03±0.37	58.80–142.60
RM sEVs	20.63±0.68	93.50±0.91	64.07±1.14	58.30–138.57
RS sEVs	14.97±1.86	98.80±2.23	72.40±2.16	60.33–168.73
**p-value**	**0.171**	**0.115**	**0.138**	

Abbreviation: HCs, healthy controls; RAs, recovered asymptomatic patients; RMs, recovered moderate patients; RSs, recovered severe and critical patients; sEVs, small extracellular vesicles.

Table S3. The size distribution of sEVs in RAs, RMs, RSs, and HCs

Proportion (%)	0-50 nm	50-100 nm	100-150 nm	150-200 nm	200-250 nm	250-300 nm
HC sEVs	0	0.7153	0.2126	0.0408	0.0312	0
RA sEVs	0	0.6910	0.2279	0.0432	0.0380	0
RM sEVs	0	0.7010	0.2397	0.0379	0.0213	0
RS sEVs	0	0.7028	0.2133	0.0473	0.0366	0
p-value	**1**	**0.319**	**0.053**	**0.282**	**0.069**	**1**

Abbreviation: HCs, healthy controls; RAs, recovered asymptomatic patients; RMs, recovered moderate patients; RSs, recovered severe and critical patients; sEVs, small extracellular vesicles.

Table S4. The detected 508 lipids

No.	Lipid Name	Ionization Mode	Class
1	FFA_12_0	Negative	FFA
2	FFA_12_1	Negative	FFA
3	FFA_14_0	Negative	FFA
4	FFA_14_1	Negative	FFA
5	FFA_16_0	Negative	FFA
6	FFA_16_1	Negative	FFA
7	FFA_16_2	Negative	FFA
8	FFA_17_0	Negative	FFA
9	FFA_17_1	Negative	FFA
10	FFA_18_0	Negative	FFA
11	FFA_18_1	Negative	FFA
12	FFA_18_2	Negative	FFA
13	FFA_18_3	Negative	FFA
14	FFA_18_4	Negative	FFA
15	FFA_20_0	Negative	FFA
16	FFA_20_1	Negative	FFA
17	FFA_20_2	Negative	FFA
18	FFA_20_3	Negative	FFA
19	FFA_20_4	Negative	FFA
20	FFA_20_5	Negative	FFA
21	FFA_22_0	Negative	FFA
22	FFA_22_1	Negative	FFA
23	FFA_22_2	Negative	FFA
24	FFA_22_3	Negative	FFA
25	FFA_22_4	Negative	FFA
26	FFA_22_5	Negative	FFA
27	FFA_22_6	Negative	FFA
28	LPA16_0	Negative	LPA
29	LPA16_1	Negative	LPA
30	LPA18_0	Negative	LPA
31	LPA18_1	Negative	LPA
32	LPA18_2	Negative	LPA
33	LPI16_0	Negative	LPI
34	LPI16_1	Negative	LPI
35	LPI18_0	Negative	LPI
36	LPI18_1	Negative	LPI
37	LPI18_2	Negative	LPI
38	LPI20_3	Negative	LPI
39	PA_14_0-16_1	Negative	PA
40	PA_16_0-16_0	Negative	PA
41	PA_16_0-16_1	Negative	PA
42	PA_16_0-18_0	Negative	PA

43	PA_16_0-18_1	Negative	PA
44	PA_16_0-18_2	Negative	PA
45	PA_16_0-20_3	Negative	PA
46	PA_16_1-16_1	Negative	PA
47	PA_16_1-18_0	Negative	PA
48	PA_16_1-18_1	Negative	PA
49	PA_16_1-18_2	Negative	PA
50	PA_18_0-18_0	Negative	PA
51	PA_18_0-18_1	Negative	PA
52	PA_18_0-18_2	Negative	PA
53	PA_18_0-18_3	Negative	PA
54	PA_18_0-20_3	Negative	PA
55	PA_18_1-18_1	Negative	PA
56	PA_18_1-18_2	Negative	PA
57	PA_18_1-20_3	Negative	PA
58	PA22_0-22_6	Negative	PA
59	PC12_0-16_0	Negative	PC
60	PC14_0-16_0	Negative	PC
61	PC14_0-16_1	Negative	PC
62	PC14_0-22_4	Negative	PC
63	PC14_1-16_0	Negative	PC
64	PC14_1-16_1	Negative	PC
65	PC16_0-16_0	Negative	PC
66	PC16_0-16_1	Negative	PC
67	PC16_0-18_0	Negative	PC
68	PC16_0-18_1	Negative	PC
69	PC16_0-18_2	Negative	PC
70	PC16_0-18_3	Negative	PC
71	PC16_0-20_0	Negative	PC
72	PC16_0-20_1	Negative	PC
73	PC16_0-20_2	Negative	PC
74	PC16_0-20_3	Negative	PC
75	PC16_0-20_4	Negative	PC
76	PC16_0-20_5	Negative	PC
77	PC16_0-22_2	Negative	PC
78	PC16_0-22_3	Negative	PC
79	PC16_0-22_4	Negative	PC
80	PC16_0-22_5	Negative	PC
81	PC16_0-22_6	Negative	PC
82	PC16_1-16_1	Negative	PC
83	PC16_1-18_0	Negative	PC
84	PC16_1-18_1	Negative	PC
85	PC16_1-18_2	Negative	PC
86	PC16_1-18_3	Negative	PC

87	PC16_1-20_0	Negative	PC
88	PC16_1-20_1	Negative	PC
89	PC16_1-20_2	Negative	PC
90	PC16_1-20_3	Negative	PC
91	PC16_1-20_4	Negative	PC
92	PC16_1-20_5	Negative	PC
93	PC18_0-18_0	Negative	PC
94	PC18_0-18_1	Negative	PC
95	PC18_0-18_2	Negative	PC
96	PC18_0-18_3	Negative	PC
97	PC18_0-20_2	Negative	PC
98	PC18_0-20_3	Negative	PC
99	PC18_0-20_5	Negative	PC
100	PC18_0-22_4	Negative	PC
101	PC18_0-22_5	Negative	PC
102	PC18_1-18_1	Negative	PC
103	PC18_1-18_2	Negative	PC
104	PC18_1-18_3	Negative	PC
105	PC18_1-20_0	Negative	PC
106	PC18_1-20_1	Negative	PC
107	PC18_1-20_2	Negative	PC
108	PC18_1-20_3	Negative	PC
109	PC18_1-22_0	Negative	PC
110	PC18_2-18_2	Negative	PC
111	PC18_2-20_0	Negative	PC
112	PC18_2-20_2	Negative	PC
113	PC18_2-20_3	Negative	PC
114	PC18_2-20_4	Negative	PC
115	PC20_0-22_0	Negative	PC
116	PC20_0-22_1	Negative	PC
117	PC20_1-22_1	Negative	PC
118	PC20_1-22_2	Negative	PC
119	PC20_2-22_0	Negative	PC
120	PC20_2-22_1	Negative	PC
121	PC20_3-22_1	Negative	PC
122	PC22_3-22_6	Negative	PC
123	PE14_0-22_5	Negative	PE
124	PE_14_1-18_0	Negative	PE
125	PE_14_1-18_1	Negative	PE
126	PE_16_0-16_0	Negative	PE
127	PE_16_0-16_1	Negative	PE
128	PE_16_0-18_0	Negative	PE
129	PE_16_0-18_1	Negative	PE
130	PE_16_0-18_2	Negative	PE

131	PE16_0-20_2	Negative	PE
132	PE_16_0-20_3	Negative	PE
133	PE16_0-20_4	Negative	PE
134	PE16_0-20_5	Negative	PE
135	PE16_0-22_4	Negative	PE
136	PE16_0-22_5	Negative	PE
137	PE16_0-22_6	Negative	PE
138	PE_16_1-16_1	Negative	PE
139	PE_16_1-18_0	Negative	PE
140	PE_16_1-18_1	Negative	PE
141	PE_16_1-18_2	Negative	PE
142	PE_18_0-18_0	Negative	PE
143	PE_18_0-18_1	Negative	PE
144	PE_18_0-18_2	Negative	PE
145	PE_18_0-18_3	Negative	PE
146	PE18_0-20_2	Negative	PE
147	PE18_0-20_3	Negative	PE
148	PE18_0-20_4	Negative	PE
149	PE18_0-20_5	Negative	PE
150	PE18_0-22_1	Negative	PE
151	PE18_0-22_4	Negative	PE
152	PE18_0-22_5	Negative	PE
153	PE18_0-22_6	Negative	PE
154	PE_18_1-18_1	Negative	PE
155	PE_18_1-18_2	Negative	PE
156	PE_18_1-18_3	Negative	PE
157	PE_18_1-20_3	Negative	PE
158	PE_18_1-20_4	Negative	PE
159	PE_18_1-20_5	Negative	PE
160	PE_18_2-18_2	Negative	PE
161	PE18_2-18_3	Negative	PE
162	PE18_2-20_2	Negative	PE
163	PE_18_2-20_3	Negative	PE
164	PE18_2-20_4	Negative	PE
165	PE_18_3-22_5	Negative	PE
166	PE20_0-22_6	Negative	PE
167	PE20_2-20_4	Negative	PE
168	PE20_4-20_4	Negative	PE
169	PE_22_0-22_4	Negative	PE
170	PE_22_1-22_3	Negative	PE
171	PE_22_3-22_6	Negative	PE
172	PG_14_0-16_0	Negative	PG
173	PG_16_0-16_0	Negative	PG
174	PG_16_0-16_1	Negative	PG

175	PG_16_0-18_0	Negative	PG
176	PG_16_0-18_1	Negative	PG
177	PG_16_0-18_2	Negative	PG
178	PG 16_0-20_2	Negative	PG
179	PG 16_0-20_3	Negative	PG
180	PG_16_1-16_1	Negative	PG
181	PG_16_1-18_0	Negative	PG
182	PG_16_1-18_1	Negative	PG
183	PG_16_1-18_2	Negative	PG
184	PG_18_0-18_0	Negative	PG
185	PG_18_0-18_1	Negative	PG
186	PG_18_0-18_2	Negative	PG
187	PG 18_0-20_4	Negative	PG
188	PG_18_1-18_1	Negative	PG
189	PG_18_1-18_2	Negative	PG
190	PG_18_1-18_3	Negative	PG
191	PG_18_2-18_2	Negative	PG
192	PI_14_0-18_0	Negative	PI
193	PI_14_0-18_1	Negative	PI
194	PI_16_0-16_0	Negative	PI
195	PI_16_0-16_1	Negative	PI
196	PI_16_0-18_0	Negative	PI
197	PI_16_0-18_1	Negative	PI
198	PI_16_0-18_2	Negative	PI
199	PI16_0-20_0	Negative	PI
200	PI16_0-20_1	Negative	PI
201	PI_16_0-20_3	Negative	PI
202	PI16_0-20_4	Negative	PI
203	PI_16_1-16_1	Negative	PI
204	PI_16_1-18_0	Negative	PI
205	PI_16_1-18_1	Negative	PI
206	PI_16_1-18_2	Negative	PI
207	PI_18_0-18_0	Negative	PI
208	PI_18_0-18_1	Negative	PI
209	PI_18_0-18_2	Negative	PI
210	PI18_0-18_3	Negative	PI
211	PI18_0-20_2	Negative	PI
212	PI18_0-20_3	Negative	PI
213	PI18_0-20_4	Negative	PI
214	PI18_0-20_5	Negative	PI
215	PI18_0-22_4	Negative	PI
216	PI_18_1-18_1	Negative	PI
217	PI_18_1-18_2	Negative	PI
218	PI_18_1-18_3	Negative	PI

219	PI_18_1-20_3	Negative	PI
220	PI18_2-18_2	Negative	PI
221	PI18_2-20_1	Negative	PI
222	PI20_0-20_3	Negative	PI
223	PI20_0-20_4	Negative	PI
224	PS_16_0-16_0	Negative	PS
225	PS_16_0-16_1	Negative	PS
226	PS_16_0-18_0	Negative	PS
227	PS_16_0-18_1	Negative	PS
228	PS_16_0-18_2	Negative	PS
229	PS_16_0-20_3	Negative	PS
230	PS_16_1-16_1	Negative	PS
231	PS_16_1-18_0	Negative	PS
232	PS_16_1-18_1	Negative	PS
233	PS_16_1-18_2	Negative	PS
234	PS_18_0-18_0	Negative	PS
235	PS_18_0-18_1	Negative	PS
236	PS_18_0-18_2	Negative	PS
237	PS_18_1-18_1	Negative	PS
238	PS_18_1-18_2	Negative	PS
239	PS_18_3-18_3	Negative	PS
240	PS_20_3-20_3	Negative	PS
241	PS_22_4-22_5	Negative	PS
242	15:0-18:1_(d7)_PA	Negative	SIL
243	15:0-18:1 (d7) PC	Negative	SIL
244	15:0-18:1_(d7)_PE	Negative	SIL
245	15:0-18:1_(d7)_PG	Negative	SIL
246	15:0-18:1_(d7)_PI	Negative	SIL
247	15:0-18:1_(d7)_PS	Negative	SIL
248	15:0-18:1 (d7) PCpos	Negative	SIL
249	15:0-18:1 (d7) DG	Positive	SIL
250	15:0-18:1 (d7)-15:0 TG	Positive	SIL
251	18:1 (d7) Chol Ester	Positive	SIL
252	18:1 (d7) LPC	Positive	SIL
253	18:1 (d7) LPE	Positive	SIL
254	18:1 (d7) MG	Positive	SIL
255	18:1 (d9) SM	Positive	SIL
256	Cholesterol (d7)	Positive	SIL
257	CE 14_0	Positive	CE
258	CE 14_1	Positive	CE
259	CE 16_0	Positive	CE
260	CE 16_1	Positive	CE
261	CE 17_0	Positive	CE
262	CE 18_0	Positive	CE

263	CE 18_2	Positive	CE
264	CE 20_0	Positive	CE
265	CE 20_2	Positive	CE
266	CE 22_0	Positive	CE
267	Cer d18_1-12_0	Positive	Cer
268	Cer d18_1-14_0	Positive	Cer
269	Cer d18_1-14_1	Positive	Cer
270	Cer d18_1-16_0	Positive	Cer
271	Cer d18_1-16_1	Positive	Cer
272	Cer d18_1-17_0	Positive	Cer
273	Cer d18_1-18_0	Positive	Cer
274	Cer d18_1-18_1	Positive	Cer
275	Cer d18_1-18_2	Positive	Cer
276	Cer d18_1-18_3	Positive	Cer
277	Cer d18_1-18_4	Positive	Cer
278	Cer d18_1-20_0	Positive	Cer
279	Cer d18_1-20_1	Positive	Cer
280	Cer d18_1-20_2	Positive	Cer
281	Cer d18_1-20_3	Positive	Cer
282	Cer d18_1-20_4	Positive	Cer
283	Cer d18_1-20_5	Positive	Cer
284	Cer d18_1-22_0	Positive	Cer
285	Cer d18_1-22_1	Positive	Cer
286	Cer d18_1-22_2	Positive	Cer
287	Cer d18_1-22_3	Positive	Cer
288	Cer d18_1-22_4	Positive	Cer
289	Cer d18_1-22_5	Positive	Cer
290	Cer d18_1-22_6	Positive	Cer
291	DAG12_0-16_0	Positive	DG
292	DAG12_0-18_2	Positive	DG
293	DAG14_0-16_0	Positive	DG
294	DAG14_0-18_0	Positive	DG
295	DAG14_0-18_1	Positive	DG
296	DAG14_0-18_2	Positive	DG
297	DAG14_1-18_1	Positive	DG
298	DAG14_1-22_4	Positive	DG
299	DAG14_1-22_5	Positive	DG
300	DAG14_1-22_6	Positive	DG
301	DAG16_0-16_0	Positive	DG
302	DAG16_0-16_1	Positive	DG
303	DAG16_0-18_0	Positive	DG
304	DAG16_0-18_1	Positive	DG
305	DAG16_0-18_2	Positive	DG
306	DAG16_0-20_1	Positive	DG

307	DAG16_0-20_4	Positive	DG
308	DAG16_1-16_1	Positive	DG
309	DAG16_1-18_0	Positive	DG
310	DAG16_1-18_1	Positive	DG
311	DAG16_1-18_2	Positive	DG
312	DAG18_0-18_0	Positive	DG
313	DAG18_0-18_1	Positive	DG
314	DAG18_0-18_2	Positive	DG
315	DAG18_0-20_0	Positive	DG
316	DAG18_0-20_1	Positive	DG
317	DAG18_1-18_1	Positive	DG
318	DAG18_1-18_2	Positive	DG
319	DAG18_1-18_3	Positive	DG
320	DAG18_1-20_0	Positive	DG
321	DAG18_1-20_1	Positive	DG
322	DAG18_2-18_2	Positive	DG
323	DAG18_2-20_0	Positive	DG
324	DAG18_2-20_1	Positive	DG
325	DAG18_2-20_2	Positive	DG
326	DAG20_0-22_1	Positive	DG
327	DAG20_0-22_6	Positive	DG
328	DAG22_0-22_1	Positive	DG
329	DAG22_0-22_2	Positive	DG
330	DAG22_2-22_3	Positive	DG
331	HexCerd18_1-12_0	Positive	HexCer
332	HexCerd18_1-14_0	Positive	HexCer
333	HexCerd18_1-14_1	Positive	HexCer
334	HexCerd18_1-16_0	Positive	HexCer
335	HexCerd18_1-16_1	Positive	HexCer
336	HexCerd18_1-18_0	Positive	HexCer
337	HexCerd18_1-18_1	Positive	HexCer
338	HexCerd18_1-18_2	Positive	HexCer
339	HexCerd18_1-18_3	Positive	HexCer
340	HexCerd18_1-18_4	Positive	HexCer
341	HexCerd18_1-20_0	Positive	HexCer
342	HexCerd18_1-20_1	Positive	HexCer
343	HexCerd18_1-20_2	Positive	HexCer
344	HexCerd18_1-20_3	Positive	HexCer
345	HexCerd18_1-20_4	Positive	HexCer
346	HexCerd18_1-20_5	Positive	HexCer
347	HexCerd18_1-22_0	Positive	HexCer
348	HexCerd18_1-22_1	Positive	HexCer
349	HexCerd18_1-22_2	Positive	HexCer
350	HexCerd18_1-22_3	Positive	HexCer

351	HexCerd18_1-22_4	Positive	HexCer
352	HexCerd18_1-22_5	Positive	HexCer
353	HexCerd18_1-22_6	Positive	HexCer
354	LPC12_0	Positive	LPC
355	LPC14_1	Positive	LPC
356	LPC15_0	Positive	LPC
357	LPC16_0	Positive	LPC
358	LPC16_1	Positive	LPC
359	LPC18_0	Positive	LPC
360	LPC18_1	Positive	LPC
361	LPC18_2	Positive	LPC
362	LPC18_3	Positive	LPC
363	LPC18_4	Positive	LPC
364	LPC20_0	Positive	LPC
365	LPC20_3	Positive	LPC
366	LPC20_4	Positive	LPC
367	LPC20_5	Positive	LPC
368	LPC22_1	Positive	LPC
369	LPC22_4	Positive	LPC
370	LPC22_6	Positive	LPC
371	LPC24_0	Positive	LPC
372	LPCO- 16_0	Positive	LPC
373	LPCO- 18_0	Positive	LPC
374	LPCP-18_1	Positive	LPC
375	LPCP-20_0	Positive	LPC
376	LPE12_0	Positive	LPE
377	LPE14_0	Positive	LPE
378	LPE14_1	Positive	LPE
379	LPE16_0	Positive	LPE
380	LPE16_1	Positive	LPE
381	LPE17_1	Positive	LPE
382	LPE18_0	Positive	LPE
383	LPE18_1	Positive	LPE
384	LPE18_2	Positive	LPE
385	LPE18_3	Positive	LPE
386	LPE18_4	Positive	LPE
387	LPE20_0	Positive	LPE
388	LPE20_1	Positive	LPE
389	LPE20_2	Positive	LPE
390	LPE20_3	Positive	LPE
391	LPE20_4	Positive	LPE
392	LPE20_5	Positive	LPE
393	LPE22_0	Positive	LPE
394	LPE22_1	Positive	LPE

395	LPE22_2	Positive	LPE
396	LPE22_3	Positive	LPE
397	LPE22_4	Positive	LPE
398	LPE22_5	Positive	LPE
399	LPE22_6	Positive	LPE
400	MAG16_0	Positive	MAG
401	MAG16_1	Positive	MAG
402	MAG18_0	Positive	MAG
403	MAG18_1	Positive	MAG
404	MAG18_2	Positive	MAG
405	MAG20_4	Positive	MAG
406	MAG22_5	Positive	MAG
407	PC16_0e-16_0	Positive	PC
408	PC16_0e-16_1	Positive	PC
409	PC16_0e-18_0	Positive	PC
410	PC16_0e-18_1	Positive	PC
411	PC16_0e-18_2	Positive	PC
412	PC16_0e-18_3	Positive	PC
413	PC16_0e-20_1	Positive	PC
414	PC16_0e-20_2	Positive	PC
415	PC16_0e-20_4	Positive	PC
416	PC16_0e-22_5	Positive	PC
417	PC16_0e-22_6	Positive	PC
418	PC16_0p-16_0	Positive	PC
419	PC16_0p-16_1	Positive	PC
420	PC16_0p-18_0	Positive	PC
421	PC16_0p-18_1	Positive	PC
422	PC16_0p-18_2	Positive	PC
423	PC16_0p-18_3	Positive	PC
424	PC16_0p-20_0	Positive	PC
425	PC16_0p-20_1	Positive	PC
426	PC16_0p-20_2	Positive	PC
427	PC16_0p-20_4	Positive	PC
428	PC16_0p-22_6	Positive	PC
429	PC18_0e-16_0	Positive	PC
430	PC18_0e-16_1	Positive	PC
431	PC18_0e-18_0	Positive	PC
432	PC18_0e-18_1	Positive	PC
433	PC18_0e-18_2	Positive	PC
434	PC18_0e-18_3	Positive	PC
435	PC18_0e-20_4	Positive	PC
436	PC18_0p-14_0	Positive	PC
437	PC18_0p-14_1	Positive	PC
438	PC18_0p-16_0	Positive	PC

439	PC18_0p-16_1	Positive	PC
440	PC18_0p-18_0	Positive	PC
441	PC18_0p-18_1	Positive	PC
442	PC18_0p-18_2	Positive	PC
443	PC18_0p-18_3	Positive	PC
444	PC18_1e-16_0	Positive	PC
445	PC18_1e-16_1	Positive	PC
446	PC18_1e-18_0	Positive	PC
447	PC18_1e-18_1	Positive	PC
448	PC18_1e-18_2	Positive	PC
449	PC18_1e-18_3	Positive	PC
450	PC18_1e-20_4	Positive	PC
451	PC18_2e-12_0	Positive	PC
452	PC18_2e-16_0	Positive	PC
453	PC18_2e-16_1	Positive	PC
454	PC18_2e-18_0	Positive	PC
455	PC18_2e-18_1	Positive	PC
456	PC18_2e-18_2	Positive	PC
457	PC18_2e-18_3	Positive	PC
458	PC18_2p-16_1	Positive	PC
459	PC18_2p-18_0	Positive	PC
460	PC18_2p-18_1	Positive	PC
461	PC18_2p-18_2	Positive	PC
462	PC18_2p-20_3	Positive	PC
463	PC18_2p-20_4	Positive	PC
464	PC18_2p-20_5	Positive	PC
465	SMd18_1-12_0	Positive	SM
466	SMd18_1-14_0	Positive	SM
467	SMd18_1-14_1	Positive	SM
468	SMd18_1-16_0	Positive	SM
469	SMd18_1-16_1	Positive	SM
470	SMd18_1-17_0	Positive	SM
471	SMd18_1-18_0	Positive	SM
472	SMd18_1-18_1	Positive	SM
473	SMd18_1-18_2	Positive	SM
474	SMd18_1-18_3	Positive	SM
475	SMd18_1-18_4	Positive	SM
476	SMd18_1-20_0	Positive	SM
477	SMd18_1-20_1	Positive	SM
478	SMd18_1-20_2	Positive	SM
479	SMd18_1-20_3	Positive	SM
480	SMd18_1-20_4	Positive	SM
481	SMd18_1-20_5	Positive	SM
482	SMd18_1-22_0	Positive	SM

483	SMd18_1-22_1	Positive	SM
484	SMd18_1-22_2	Positive	SM
485	SMd18_1-22_3	Positive	SM
486	SMd18_1-22_4	Positive	SM
487	SMd18_1-22_5	Positive	SM
488	SMd18_1-22_6	Positive	SM
489	TAG46_2	Positive	TG
490	TAG48_0	Positive	TG
491	TAG48_1	Positive	TG
492	TAG48_2	Positive	TG
493	TAG50_0	Positiv	TG
494	TAG50_1	Positive	TG
495	TAG50_2	Positive	TG
496	TAG50_3	Positive	TG
497	TAG50_4	Positive	TG
498	TAG52_2	Positive	TG
499	TAG52_3	Positive	TG
500	TAG52_4	Positive	TG
501	TAG52_5	Positive	TG
502	TAG52_6	Positive	TG
503	TAG54_1	Positive	TG
504	TAG54_2	Positive	TG
505	TAG54_3	Positive	TG
506	TAG54_4	Positive	TG
507	TAG54_5	Positive	TG
508	TAG54_6	Positiv	TG

Abbreviation:

CE, Cholesteryl ester; Cer, Ceramide; DAG, Diacylglycerol; FFA, free fatty acid; HexCer, Hexosylceramide; LPA, lysoPhosphatidic acid; LPC, lysoPhosphatidylcholine; LPE, lysoPhosphatidylethanolamine; MAG, Monoacylglycerol; PA, phosphatidic acid; PC, phosphatidylcholine; PE, phosphatidylethanolamine; PG, Phosphatidylglycerol; SM, sphingomyelin; TAG, triacylglycerol.

Table S6. Differential lipids

Lipids	RA vs. HC		RM vs. HC		RS vs. HC		RS vs. RM	
	p-value	FC	p-value	FC	p-value	FC	p-value	FC
DAG(18:0/18:0)	0.0452*	0.0004	NS	NS	NS	NS	NS	NS
DAG(16:0/16:0)	NS	NS	0.0022**	0.3776	NS	NS	NS	NS
DAG(16:1/18:0)	NS	NS	0.0083**	0.0022	NS	NS	0.0118*	62.2906
DAG(18:0/18:2)	NS	NS	0.0289*	1.3998	NS	NS	0.0073**	0.7144
DAG(18:1/20:0)	NS	NS	0.0318*	0.4852	NS	NS	NS	NS
DAG(14:0/16:0)	NS	NS	0.0342*	0.4391	0.0001***	0.3468	NS	NS
DAG(16:0/16:1)	NS	NS	NS	NS	0.0002***	0.2289	0.0000***	0.2556
DAG(16:1/16:1)	NS	NS	NS	NS	0.0005***	0.4692	0.0013**	0.4486
DAG(14:0/18:0)	NS	NS	NS	NS	0.0024**	0.4163	0.0032**	0.4570
DAG(14:0/18:2)	NS	NS	NS	NS	0.0175*	79.5900	NS	NS
DAG(18:1/18:3)	NS	NS	NS	NS	NS	NS	0.0153*	3.5526
DAG(16:1/18:2)	NS	NS	NS	NS	NS	NS	0.0180*	0.2912
DAG(16:1/18:1)	NS	NS	NS	NS	NS	NS	0.0357*	0.6762
FFA(22:5)	0.0239*	1.3138	0.0001***	1.4707	0.0000***	1.6635	NS	NS
FFA(22:3)	NS	NS	0.0211*	0.7927	0.0032**	0.7727	NS	NS
FFA(20:5)	NS	NS	NS	NS	0.0000***	0.3460	0.0007***	0.4288
FFA(20:4)	NS	NS	NS	NS	0.0000***	0.3142	0.0013**	0.4178
FFA(16:2)	NS	NS	NS	NS	0.0008***	2.7215	0.0089**	2.5165
FFA(20:3)	NS	NS	NS	NS	0.0027**	0.4839	0.0137*	0.5805
FFA(14:0)	NS	NS	NS	NS	0.0157*	1.1680	NS	NS
HexCer(d18:1/22:1)	NS	NS	0.0258*	1.1728	NS	NS	NS	NS

LPC(22:1)	NS	NS	0.0017**	1.5361	0.0184*	1.3952	NS	NS
LPC(15:0)	NS	NS	0.0025**	1.4402	0.0034**	1.3768	NS	NS
LPC(O/16:0)	NS	NS	0.0051**	1.3959	0.0035**	1.5499	NS	NS
LPC(18:3)	NS	NS	0.0127*	1.4367	NS	NS	NS	NS
LPC(16:0)	NS	NS	0.0172*	1.0746	NS	NS	NS	NS
LPC(O/18:0)	NS	NS	0.0211*	1.2171	0.0243*	1.2419	NS	NS
LPC(18:0)	NS	NS	0.0294*	1.0667	0.0218*	1.0644	NS	NS
LPC(16:1)	NS	NS	NS	NS	0.0161*	1.5825	NS	NS
LPC(18:1)	NS	NS	NS	NS	0.0360*	12.9130	NS	NS
LPC(20:0)	NS	NS	NS	NS	0.0411*	1.2983	NS	NS
LPE(14:0)	NS	NS	0.0017**	1.6180	0.0100*	1.6455	NS	NS
LPE(20:5)	NS	NS	0.0169*	1.5349	NS	NS	0.0052**	0.6646
LPE(20:4)	NS	NS	NS	NS	0.0016**	1.9855	NS	NS
LPE(20:3)	NS	NS	NS	NS	0.0090**	1.8558	NS	NS
LPE(22:6)	NS	NS	NS	NS	0.0162*	1.7027	NS	NS
LPE(20:0)	NS	NS	NS	NS	0.0492*	1.5272	NS	NS
LPE(18:0)	NS	NS	NS	NS	NS	NS	0.0104*	0.7220
LPE(20:2)	NS	NS	NS	NS	NS	NS	0.0130*	0.4428
MAG(22:5)	NS	NS	NS	NS	0.0325*	1.4666	0.0284*	1.3445
MAG(16:1)	NS	NS	NS	NS	NS	NS	0.0004***	2.6846
PA(16:0/18:0)	NS	NS	NS	NS	0.0262*	3.3436	0.0483*	1.8069
PC(16:0/22:3)	0.0144*	1.1583	NS	NS	0.0022**	1.2545	NS	NS
PC(16:0/20:3)	0.0218*	0.6984	NS	NS	0.0150*	0.7121	NS	NS
PC(16:0p/16:0)	0.0443*	1.3863	NS	NS	0.0031**	1.9793	0.0093**	1.5866
PC(18:2p/18:2)	NS	NS	0.0007***	1.3690	NS	NS	0.0026**	0.6753
PC(18:2e/16:0)	NS	NS	0.0013**	1.7085	NS	NS	0.0032**	0.7405

PC(16:0p/22:6)	NS	NS	0.0039**	1.8914	0.0232*	1.8199	NS	NS
PC(18:2p/20:4)	NS	NS	0.0101*	1.9928	0.0374*	1.7423	NS	NS
PC(16:0p/18:1)	NS	NS	0.0118*	1.3916	NS	NS	0.0094**	0.7512
PC(16:0e/16:0)	NS	NS	0.0135*	1.1504	0.0065**	1.2329	NS	NS
PC(18:0p/16:0)	NS	NS	0.0211*	2.8662	0.0023**	4.2032	NS	NS
PC(18:1e/16:0)	NS	NS	0.0371*	3.3172	0.0035**	4.8857	NS	NS
PC(16:0e/18:1)	NS	NS	0.0390*	1.9566	0.0054**	3.4696	NS	NS
PC(16:0e/18:0)	NS	NS	0.0430*	1.1957	0.0008***	1.3743	0.0237*	1.1494
PC(16:0e/16:1)	NS	NS	NS	NS	0.0002***	2.2169	0.0006***	1.8331
PC(18:0p/14:0)	NS	NS	NS	NS	0.0004***	1.8828	0.0005***	1.5671
PC(18:0e/16:0)	NS	NS	NS	NS	0.0005***	1.3554	0.0213*	1.1086
PC(18:0e/18:0)	NS	NS	NS	NS	0.0007***	1.3099	0.0005***	1.2586
PC(16:0e/20:2)	NS	NS	NS	NS	0.0015**	1.3457	0.0001***	1.4145
PC(18:1e/18:1)	NS	NS	NS	NS	0.0016**	519.8399	0.0056**	18.2632
PC(16:0p/18:0)	NS	NS	NS	NS	0.0024**	3.1187	0.0178*	1.3593
PC(14:0/16:0)	NS	NS	NS	NS	0.0035**	1.6500	NS	NS
PC(16:0e/20:4)	NS	NS	NS	NS	0.0063**	1.3920	NS	NS
PC(16:0p/20:1)	NS	NS	NS	NS	0.0107*	1.2465	0.0020**	1.3002
PC(18:0p/18:0)	NS	NS	NS	NS	0.0162*	0.8728	NS	NS
PC(16:0/18:0)	NS	NS	NS	NS	0.0194*	1.5033	NS	NS
PC(18:2e/12:0)	NS	NS	NS	NS	0.0209*	1.3505	NS	NS
PC(18:0p/18:1)	NS	NS	NS	NS	0.0250*	38.7653	0.0011**	969.6255
PC(16:0p/20:0)	NS	NS	NS	NS	0.0282*	0.8703	NS	NS
PC(16:0/20:0)	NS	NS	NS	NS	0.0300*	1.4133	0.0011**	2.2353
PC(16:0p/18:3)	NS	NS	NS	NS	0.0310*	1.2043	NS	NS
PC(18:2e/18:0)	NS	NS	NS	NS	0.0382*	1.1427	NS	NS

PC(18:2/20:3)	NS	NS	NS	NS	0.0453*	1.2829	NS	NS
PC(16:0/20:1)	NS	NS	NS	NS	NS	NS	0.0015**	1.3627
PC(16:0p/16:1)	NS	NS	NS	NS	NS	NS	0.0082**	16.4773
PC(18:2/20:2)	NS	NS	NS	NS	NS	NS	0.0185*	1.1956
PC(16:0/22:5)	NS	NS	NS	NS	NS	NS	0.0224*	5.5341
PC(16:0/22:6)	NS	NS	NS	NS	NS	NS	0.0237*	1.2426
PC(16:0e/18:2)	NS	NS	NS	NS	NS	NS	0.0258*	0.0808
PE(16:0/22:4)	NS	NS	0.0281*	1.1227	NS	NS	NS	NS
PE(18:0/22:5)	NS	NS	0.0396*	1.1662	0.0032**	1.5394	NS	NS
PE(16:0/18:0)	NS	NS	NS	NS	0.0112*	1.3558	0.0011**	1.3897
PI(16:0/20:3)	NS	NS	NS	NS	0.0132*	13.0577	0.0043**	13.0577
PI(18:0/20:3)	NS	NS	NS	NS	0.0450*	1.8966	NS	NS
PI(16:0/16:0)	NS	NS	NS	NS	NS	NS	0.0137*	1.4627
SM(d18:1/18:4)	NS	NS	0.0116*	2.6862	NS	NS	NS	NS
SM(d18:1/16:0)	NS	NS	0.0400*	82.5216	NS	NS	NS	NS
SM(d18:1/20:5)	NS	NS	NS	NS	0.0005***	2.4936	0.0032**	1.5085
SM(d18:1/20:4)	NS	NS	NS	NS	0.0010**	2.4655	NS	NS
SM(d18:1/22:3)	NS	NS	NS	NS	0.0029**	1.6366	NS	NS
SM(d18:1/22:0)	NS	NS	NS	NS	0.0042**	1.3313	0.0407*	1.3135
SM(d18:1/22:4)	NS	NS	NS	NS	0.0159*	181.9263	NS	NS
SM(d18:1/20:2)	NS	NS	NS	NS	0.0406*	1.1671	0.0049**	1.2470
SM(d18:1/18:3)	NS	NS	NS	NS	0.0407*	1.4917	NS	NS
SM(d18:1/18:2)	NS	NS	NS	NS	0.0416*	71.6994	0.0001***	71.6994
SM(d18:1/20:1)	NS	NS	NS	NS	0.0440*	1.1761	0.0019**	1.3214
SM(d18:1/22:6)	NS	NS	NS	NS	NS	NS	0.0278*	0.5009
TAG(50:0)	NS	NS	0.0139*	0.0317	NS	NS	0.0010***	43.3016

TAG(52:5)	NS	NS	0.0220*	0.0951	NS	NS	NS	NS
TAG(50:2)	NS	NS	NS	NS	0.0030**	0.0885	NS	NS
TAG(48:1)	NS	NS	NS	NS	0.0362*	0.8801	0.0173*	0.9376
TAG(52:4)	NS	NS	NS	NS	NS	NS	0.0037**	1.3947
TAG(54:5)	NS	NS	NS	NS	NS	NS	0.0130*	0.8682
TAG(50:1)	NS	NS	NS	NS	NS	NS	0.0138*	0.8277

Abbreviation: CE, Cholesteryl ester; Cer, Ceramide; DAG, Diacylglycerol; FC, fold change; FFA, free fatty acid; HCs, healthy controls; HexCer, Hexosylceramide; LPA, lysoPhosphatidic acid; LPC, lysoPhosphatidylcholine; LPE, lysoPhosphatidylethanolamine; MAG, Monoacylglycerol; NS, non-significantly changed; PA, phosphatidic acid; PC, phosphatidylcholine; PE, phosphatidylethanolamine; PG, Phosphatidylglycerol; RAs, recovered asymptomatic patients; RMs, recovered moderate patients; RSs, recovered severe and critical patients; SM, sphingomyelin; TAG, triacylglycerol.

*p<0.05, **p<0.01, ***p<0.001.

Table S7. Relationship between the differential lipids of RM vs. HC and clinical indices

Lipids	Clinical indices	Correlation coefficient	p-value
DAG(14.0/16.0)	APTT	0.3275	0.0216*
DAG(14.0/16.0)	HCT	-0.3002	0.0361*
DAG(14.0/16.0)	UA	-0.3044	0.0335*
DAG(16.0/16.0)	PDW	-0.3082	0.0312*
DAG(16.0/16.0)	HCT	-0.3692	0.0090**
DAG(16.1/18.0)	UA	0.3296	0.0207*
DAG(18.0/18.2)	UA	-0.3524	0.0130*
DAG(18.0/18.2)	Mg	-0.3791	0.0102*
DAG(18.0/18.2)	Creatinine	-0.3861	0.0061**
DAG(18.0/18.2)	BUN	-0.4173	0.0029**
DAG(18.1/20.0)	MCH	0.3451	0.0152*
DAG(18.1/20.0)	MCV	0.2868	0.0457*
DAG(18.1/20.0)	α-HBDH	-0.2894	0.0437*
DAG(18.1/20.0)	PDW	-0.3100	0.0302*
DAG(18.1/20.0)	PLT	-0.3129	0.0286*
DAG(18.1/20.0)	CK-MB activity	-0.3199	0.0250*
DAG(18.1/20.0)	PCT	-0.4133	0.0032**
FFA(22.5)	CO2	-0.3431	0.0158*
FFA(22:3)	RBC	0.3032	0.0342*
HexCer(d18:1/22:1)	Basophils%	0.3432	0.0158*
LPC(15.0)	Basophils%	0.3659	0.0097**
LPC(15.0)	Basophils	0.3265	0.0220*
LPC(15.0)	MCH	-0.2874	0.0453*
LPC(15.0)	AST	-0.3009	0.0356*
LPC(15.0)	CK-MB	-0.3089	0.0326*
LPC(15.0)	HGB	-0.3427	0.0159*
LPC(16.0)	Basophils%	0.3504	0.0136*
LPC(16.0)	Eosinophils%	0.3206	0.0247*
LPC(16.0)	Basophils	0.2834	0.0484*
LPC(16.0)	HGB	-0.3109	0.0297*
LPC(18.0)	Eosinophils%	0.2857	0.0466*
LPC(18.0)	Mg	-0.3074	0.0399*
LPC(18.0)	HGB	-0.3233	0.0235*
LPC(18.3)	A/G	0.3187	0.0256*
LPC(18.3)	Basophils%	0.2942	0.0402*
LPC(18.3)	RBC	-0.2967	0.0384*
LPC(18.3)	Creatinine	-0.3160	0.0270*
LPC(18.3)	ALT	-0.3502	0.0136*
LPC(18.3)	r-GT	-0.3520	0.0131*
LPC(18.3)	HGB	-0.4010	0.0043**

LPC(22.1)	Basophils%	0.3114	0.0294*
LPC(22.1)	Ca	0.3093	0.0387*
LPC(O/16:0)	Basophils%	0.3851	0.0063**
LPC(O/16:0)	Basophils	0.3322	0.0197*
LPC(O/16:0)	Eosinophils	0.2843	0.0477*
LPC(O/16:0)	Eosinophils%	0.2822	0.0495*
LPC(O/16:0)	Creatinine	-0.2830	0.0488*
LPC(O/16:0)	CK-MB	-0.2938	0.0427*
LPC(O/16:0)	AST	-0.3125	0.0288*
LPC(O/16:0)	HGB	-0.3381	0.0175*
LPC(O/18:0)	Eosinophils	0.3171	0.0264*
LPC(O/18:0)	Eosinophils%	0.3105	0.0299*
LPC(O/18:0)	Basophils%	0.3044	0.0334*
LPC(O/18:0)	Creatinine	-0.2966	0.0385*
LPC(O/18:0)	CK-MB	-0.2981	0.0396*
LPC(O/18:0)	AST	-0.3304	0.0204*
LPC(O/18:0)	HGB	-0.3607	0.0109*
LPE(14:0)	Basophils%	0.4247	0.0024**
LPE(14:0)	Basophils	0.3604	0.0110*
LPE(14:0)	Ca	0.3337	0.0251*
LPE(14:0)	TT	0.3070	0.0319*
LPE(14:0)	α-HBDH	0.3045	0.0334*
LPE(14:0)	ALT	-0.2928	0.0412*
LPE(14:0)	DBIL	-0.3096	0.0304*
LPE(14:0)	RBC	-0.3293	0.0209*
LPE(14:0)	Creatinine	-0.3547	0.0124*
LPE(14:0)	AST	-0.3583	0.0115*
LPE(14:0)	CO2	-0.3972	0.0047**
LPE(14:0)	IBIL	-0.4033	0.0041**
LPE(14:0)	TBIL	-0.4045	0.0039**
LPE(14:0)	HGB	-0.4732	0.0006***
LPE(20:5)	Basophils%	0.3740	0.0081**
LPE(20:5)	Basophils	0.3423	0.0161*
LPE(20:5)	Eosinophils%	0.3404	0.0167*
LPE(20:5)	Eosinophils	0.2991	0.0368*
LPE(20:5)	HGB	-0.2880	0.0447*
LPE(20:5)	MCV	-0.2935	0.0407*
PC(16:0e/16:0)	α-HBDH	0.3155	0.0272*
PC(16:0e/16:0)	Basophils	0.2897	0.0435*
PC(16:0e/16:0)	APTT	0.2875	0.0451*
PC(16:0e/16:0)	AST	-0.3655	0.0098**
PC(16:0e/18:0)	Eosinophils	0.2915	0.0422*
PC(16:0e/18:0)	TT	0.2869	0.0456*
PC(16:0e/18:0)	Creatinine	-0.2893	0.0438*

PC(16:0e/18:0)	CO2	-0.2903	0.0430*
PC(16:0e/18:0)	CK-MB	-0.2936	0.0428*
PC(16:0e/18:0)	AST	-0.3292	0.0209*
PC(16:0e/18:0)	MCH	-0.3336	0.0191*
PC(16:0e/18:0)	FIB	-0.3503	0.0136*
PC(16:0e/18:1)	CRP	0.3650	0.0099**
PC(16:0e/18:1)	Albumin	-0.2839	0.0481*
PC(16:0e/18:1)	Creatinine	-0.3050	0.0331*
PC(16:0e/18:1)	DBIL	-0.3390	0.0172*
PC(16:0e/18:1)	HGB	-0.3748	0.0080**
PC(16:0e/18:1)	CO2	-0.3973	0.0047**
PC(16:0e/18:1)	IBIL	-0.4462	0.0013**
PC(16:0e/18:1)	TBIL	-0.4510	0.0011**
PC(16:0p/18:1)	CK-MB activity	0.3433	0.0157*
PC(16:0p/18:1)	RBC	-0.2897	0.0435*
PC(16:0p/18:1)	HGB	-0.3027	0.0345*
PC(16:0p/18:1)	CO2	-0.3119	0.0291*
PC(16:0p/18:1)	AST	-0.3305	0.0204*
PC(16:0p/18:1)	UA	-0.3866	0.0061**
PC(16:0p/18:1)	Creatinine	-0.3970	0.0047**
PC(18:0p/16:0)	CRP	0.3163	0.0268*
PC(18:0p/16:0)	Basophils%	0.3112	0.0295*
PC(18:0p/16:0)	CK-MB	-0.2912	0.0446*
PC(18:0p/16:0)	RBC	-0.2951	0.0396*
PC(18:0p/16:0)	Creatinine	-0.3081	0.0312*
PC(18:0p/16:0)	DBIL	-0.3117	0.0292*
PC(18:0p/16:0)	CO2	-0.3816	0.0068**
PC(18:0p/16:0)	HGB	-0.3930	0.0052**
PC(18:0p/16:0)	TBIL	-0.4349	0.0018**
PC(18:0p/16:0)	IBIL	-0.4362	0.0017**
PC(18:1e/16:0)	CRP	0.3542	0.0125*
PC(18:1e/16:0)	Basophils%	0.2858	0.0465*
PC(18:1e/16:0)	HGB	-0.3303	0.0205*
PC(18:1e/16:0)	DBIL	-0.3453	0.0151*
PC(18:1e/16:0)	CO2	-0.4210	0.0026**
PC(18:1e/16:0)	IBIL	-0.4468	0.0013**
PC(18:1e/16:0)	TBIL	-0.4514	0.0011**
PC(18:2e/16:0)	CK-MB activity	0.3492	0.0139*
PC(18:2e/16:0)	AST	-0.2925	0.0414*
PC(18:2e/16:0)	Creatinine	-0.3451	0.0152*
PC(18:2e/16:0)	CO2	-0.3642	0.0101*
PC(18:2p/18:2)	α-HBDH	0.4014	0.0043**
PC(18:2p/18:2)	Basophils%	0.3359	0.0183*
PC(18:2p/18:2)	Basophils	0.3176	0.0262*

PC(18:2p/18:2)	TT	0.2958	0.0390*
PC(18:2p/18:2)	CK-MB activity	0.2925	0.0414*
PC(18:2p/18:2)	BUN	-0.2860	0.0464*
PC(18:2p/18:2)	CO2	-0.3038	0.0338*
PC(18:2p/18:2)	AST	-0.4041	0.0040**
PC(18:2p/18:2)	Creatinine	-0.4532	0.0011**
PE(16.0/22.4)	Creatinine	-0.2841	0.0479*
PE(18.0/22.5)	Basophils%	0.2867	0.0458*
PE(18.0/22.5)	HCT	-0.2843	0.0477*
PE(18.0/22.5)	Creatinine	-0.3113	0.0295*
PE(18.0/22.5)	UA	-0.3298	0.0207*
PE(18.0/22.5)	Glucose	-0.3325	0.0256*
PE(18.0/22.5)	RBC	-0.3997	0.0044*
PE(18.0/22.5)	HGB	-0.4598	0.0009***
SM(d18:1/16:0)	Basophils%	0.3069	0.0320*
SM(d18:1/16:1)	APTT	0.2900	0.0433*
SM(d18:1/18:4)	APTT	0.2837	0.0482*
SM(d18:1/18:4)	MCHC	-0.2875	0.0452*
SM(d18:1/18:4)	CK-MB	-0.3112	0.0313*
SM(d18:1/18:4)	AST	-0.3285	0.0212*
SM(d18:1/18:4)	HGB	-0.3706	0.0088**
SM(d18:1/18:4)	UA	-0.3763	0.0077**
SM(d18:1/18:4)	Creatinine	-0.4048	0.0039**
TAG(52:5)	CRP	0.3286	0.0211*
TAG(52:5)	CO2	0.2869	0.0456*
TAG(52:5)	Monocytes	-0.3621	0.0106*
TAG(52:5)	Monocytes%	-0.3723	0.0084**

Abbreviation: ALP, alkaline phosphatase; ALT, alanine aminotransferase; APTT, activated partial thromboplastin time; AST, aspartate aminotransferase; BUN, blood urea nitrogen; Ca, calcium; CK-MB, creatine kinase-myocardial band; CRP, C-reactive protein; DAG, diacylglycerol; DBIL, direct bilirubin; FIB, FFA, free fatty acid; fibrinogen; HCs, healthy controls; IBIL, indirect bilirubin; INR, international normalized ratio; LDH, lactate dehydrogenase; LPC, lysoPhosphatidylcholine; LPE, lysoPhosphatidylethanolamine; M, moderate; MCH, mean corpuscular hemoglobin; MCHC, mean corpuscular hemoglobin concentration; MCV, mean corpuscular volume; Mg, magnesium; P, phosphorus; PC, phosphatidylcholine; PCT, plateletocrit; PDW, platelet distribution width; PE, phosphatidylethanolamine; PT, prothrombin time; PI, phosphatidylinositol; RAs, recovered asymptomatic patients; RBC, red blood cell; RDW, red blood cell volume distribution width; RMs, recovered moderate patients; RSs, recovered critical and severe patients; S, critical and severe; SM, sphingomyelin; TAG, triacylglycerol; TBIL, total bilirubin; TT, thrombin time; UA, urine acid; WBC, white blood cell; α-HBDH, α-hydroxybutyrate dehydrogenase; γ-GT, γ-glutamyl transpeptidase; Statistical significance was set at 2-sided. *$p<0.05$, **$p<0.01$, ***$p<0.001$.

Table S8. Relationship between the differential lipids of RS vs. HC and clinical indices

Lipids	clinical indices	Correlation coefficient	p-value
DAG(14:0/16:0)	HGB	0.4017	0.0038**
DAG(14:0/16:0)	Mg	0.3902	0.0051**
DAG(14:0/16:0)	Creatinine	0.3745	0.0074**
DAG(14:0/16:0)	PDW	0.3262	0.0208*
DAG(14:0/16:0)	HCT	0.3247	0.0214*
DAG(14:0/16:0)	RBC	0.3158	0.0255*
DAG(14:0/16:0)	Ca	-0.2823	0.0470*
DAG(14:0/18:0)	PDW	0.4229	0.0022**
DAG(14:0/18:0)	MPV	0.3219	0.0226*
DAG(14:0/18:0)	Mg	0.3057	0.0308*
DAG(14:0/18:2)	MCHC	-0.3552	0.0114*
DAG(14:0/18:2)	HGB	-0.3393	0.0159*
DAG(14:0/18:2)	Ca	0.3056	0.0309*
DAG(14:0/18:2)	CO2	-0.3241	0.0319*
DAG(14:0/18:2)	TT	-0.3059	0.0489*
DAG(16:0/16:1)	PDW	0.5091	0.0002***
DAG(16:0/16:1)	Creatinine	0.3522	0.0121*
DAG(16:0/16:1)	MPV	0.3452	0.0141*
DAG(16:0/16:1)	HGB	0.3092	0.0289*
DAG(16:0/16:1)	PT	0.3265	0.0348*
DAG(16:0/16:1)	INR	0.3265	0.0348*
DAG(16:0/16:1)	Mg	0.2957	0.0371*
DAG(16:0/16:1)	Ca	-0.2881	0.0424*
DAG(16:0/16:1)	PDW	0.3731	0.0076**
DAG(16:0/16:1)	Mg	0.3586	0.0105*
DAG(16:0/16:1)	PT	0.3888	0.0109*
DAG(16:0/16:1)	INR	0.3888	0.0109*
FFA(14:0)	FIB	0.3939	0.0099**
FFA(14:0)	Mg	-0.3195	0.0237*
FFA(14:0)	ALP	-0.2996	0.0345*
FFA(16:2)	Mg	-0.4138	0.0028**
FFA(16:2)	APTT	-0.4156	0.0062**
FFA(20:3)	ALP	0.5220	0.0001***
FFA(20:3)	Mg	0.4069	0.0034**
FFA(20:3)	FIB	-0.3532	0.0218*
FFA(20:3)	Ca	-0.3146	0.0261*
FFA(20:3)	Creatintine	0.3137	0.0265*
FFA(20:3)	Glucose	-0.3012	0.0336*
FFA(20:3)	HCT	0.2966	0.0365*
FFA(20:3)	PT	0.3127	0.0438*
FFA(20:3)	INR	0.3127	0.0438*

FFA(20:4)	Mg	0.5258	0.0001***
FFA(20:4)	CO2	0.4441	0.0025**
FFA(20:4)	HGB	0.3636	0.0094**
FFA(20:4)	HCT	0.2929	0.0390*
FFA(20:4)	Ca	-0.2900	0.0411*
FFA(20:4)	ALP	0.2897	0.0413*
FFA(20:4)	RBC	0.2846	0.0452*
FFA(20:5)	Mg	0.4904	0.0003***
FFA(20:5)	CO2	0.4550	0.0019**
FFA(20:5)	HGB	0.3491	0.0130*
FFA(20:5)	Glucose	-0.2991	0.0349*
FFA(22:3)	Ca	-0.3145	0.0261*
FFA(22:3)	ALP	0.2969	0.0363*
FFA(22:3)	RDW	-0.2962	0.0367*
FFA(22:5)	HCT	-0.3730	0.0076**
FFA(22:5)	Glucose	0.3668	0.0088**
FFA(22:5)	Mg	-0.3564	0.0111*
FFA(22:5)	Ca	0.3555	0.0113*
FFA(22:5)	Creatinine	-0.3543	0.0116*
FFA(22:5)	HGB	-0.3390	0.0160*
FFA(22:5)	RBC	-0.3178	0.0245*
FFA(22:5)	ALP	-0.3037	0.0320*
LPC(15:0)	DBIL	-0.4795	0.0004***
LPC(15:0)	TBIL	-0.4443	0.0012**
LPC(15:0)	Creatinine	-0.4420	0.0013**
LPC(15:0)	HCT	-0.4089	0.0032**
LPC(15:0)	IBIL	-0.4063	0.0034**
LPC(15:0)	HGB	-0.3919	0.0049**
LPC(15:0)	WBC	-0.3682	0.0085**
LPC(15:0)	Neutrophils	-0.3637	0.0094**
LPC(15:0)	RBC	-0.3625	0.0097**
LPC(15:0)	r.GT	-0.3621	0.0098**
LPC(15:0)	Ca	0.3413	0.0153*
LPC(15:0)	CO2	-0.3578	0.0171*
LPC(15:0)	Albumin	-0.3318	0.0186*
LPC(15:0)	Monocytes	-0.3124	0.0272*
LPC(15:0)	AST	-0.3105	0.0282
LPC(16:1)	DBIL	-0.4404	0.0014**
LPC(16:1)	TBIL	-0.4323	0.0017**
LPC(16:1)	IBIL	-0.4125	0.0029**
LPC(16:1)	Eosinophils	-0.3494	0.0129*
LPC(16:1)	RDW	0.3280	0.0201*
LPC(16:1)	Basophils	-0.3199	0.0236*
LPC(16:1)	Eosinophils%	-0.3033	0.0323*

LPC(16:1)	Creatinine	-0.2974	0.0359*
LPC(16:1)	CO2	-0.3078	0.0421*
LPC(22:1)	WBC	-0.4565	0.0009***
LPC(22:1)	Neutrophils	-0.3802	0.0065**
LPC(22:1)	Albumin	-0.3639	0.0094**
LPC(22:1)	Monocytes	-0.2866	0.0436*
LPC(22:1)	Lymphocytes	-0.2814	0.0478*
LPC(O/16:0)	Creatinine	-0.4860	0.0003***
LPC(O/16:0)	DBIL	-0.4584	0.0008***
LPC(O/16:0)	HCT	-0.4380	0.0015**
LPC(O/16:0)	TBIL	-0.4303	0.0018**
LPC(O/16:0)	HGB	-0.4211	0.0023**
LPC(O/16:0)	Neutrophils	-0.3987	0.0041**
LPC(O/16:0)	WBC	-0.3974	0.0043**
LPC(O/16:0)	IBIL	-0.3924	0.0048**
LPC(O/16:0)	RBC	-0.3738	0.0075**
LPC(O/16:0)	Ca	0.3734	0.0076**
LPC(O/16:0)	r-GT	-0.3504	0.0126*
LPC(O/16:0)	Monocytes	-0.3410	0.0154*
LPC(O/16:0)	CO2	-0.3626	0.0156*
LPC(O/16:0)	AST	-0.3211	0.0230*
LPC(O/16:0)	Albumin	-0.2992	0.0348*
LPC(O/16:0)	LDH	-0.2936	0.0385*
LPC(O/18:0)	Creatinine	-0.4313	0.0018**
LPC(O/18:0)	r-GT	-0.4198	0.0024**
LPC(O/18:0)	AST	-0.4195	0.0024**
LPC(O/18:0)	DBIL	-0.4075	0.0033**
LPC(O/18:0)	Albumin	-0.3673	0.0087**
LPC(O/18:0)	TBIL	-0.3639	0.0094**
LPC(O/18:0)	ALT	-0.3316	0.0187*
LPC(O/18:0)	IBIL	-0.3305	0.0191*
LPC(O/18:0)	HCT	-0.3147	0.0260*
LPC(O/18:0)	RBC	-0.3145	0.0261*
LPC(O/18:0)	LDH	-0.3111	0.0279*
LPC(O/18:0)	HGB	-0.3097	0.0286*
LPC(O/18:0)	CO2	-0.3224	0.0328*
LPC(O/18:0)	WBC	-0.2960	0.0369*
LPC(O/18:0)	TP	-0.2869	0.0434*
LPC(O/18:0)	Monocytes	-0.2838	0.0458*
LPE(14:0)	IBIL	-0.4916	0.0003***
LPE(14:0)	TBIL	-0.4535	0.0009***
LPE(14:0)	DBIL	-0.3622	0.0097**
LPE(14:0)	Glucose	0.3445	0.0143*
LPE(14:0)	Eosinophils	-0.3220	0.0226*

LPE(14:0)	Basophils	-0.2828	0.0466^{*}
LPE(14:0)	CO2	-0.2994	0.0484^{*}
LPE(20:3)	DBIL	-0.5377	0.0001^{***}
LPE(20:3)	TBIL	-0.5208	0.0001^{***}
LPE(20:3)	IBIL	-0.4838	0.0004^{***}
LPE(20:3)	Eosinophils	-0.3750	0.0073^{**}
LPE(20:3)	Monocytes	-0.3363	0.0169^{*}
LPE(20:3)	Creatinine	-0.3340	0.0178^{*}
LPE(20:3)	Eosinophils%	-0.3053	0.0311^{*}
LPE(20:3)	CO2	-0.3212	0.0335^{*}
LPE(20:3)	α-HBDH	0.3262	0.0350^{*}
LPE(20:3)	LDH	-0.2818	0.0474^{*}
LPE(20:3)	Neutrophils	-0.2792	0.0496^{*}
LPE(20:4)	DBIL	-0.5054	0.0002^{***}
LPE(20:4)	TBIL	-0.4558	0.0009^{***}
LPE(20:4)	Creatinine	-0.4537	0.0009^{***}
LPE(20:4)	IBIL	-0.4044	0.0036^{**}
LPE(20:4)	HCT	-0.3807	0.0064^{**}
LPE(20:4)	CO2	-0.3821	0.0105^{*}
LPE(20:4)	HGB	-0.3475	0.0134^{*}
LPE(20:4)	PDW	-0.3377	0.0165^{*}
LPE(20:4)	Neutrophils	-0.3345	0.0176^{*}
LPE(20:4)	Monocytes	-0.2974	0.0359^{*}
LPE(20:4)	RBC	-0.2948	0.0377^{*}
LPE(20:4)	Eosinophils	-0.2939	0.0383^{*}
LPE(20:4)	WBC	-0.2864	0.0437^{*}
PC(14:0/16:0)	DBIL	-0.3430	0.0147^{*}
PC(14:0/16:0)	TBIL	-0.3345	0.0176^{*}
PC(14:0/16:0)	IBIL	-0.3179	0.0244^{*}
PC(14:0/16:0)	CO2	-0.3190	0.0348^{*}
PC(14:0/16:0)	HGB	-0.2983	0.0354^{*}
PC(14:0/16:0)	APTT	-0.3102	0.0456^{*}
PC(16:0/18:0)	Neutrophils	-0.4760	0.0005^{***}
PC(16:0/18:0)	WBC	-0.4116	0.0030^{**}
PC(16:0/18:0)	IBIL	-0.4092	0.0032^{**}
PC(16:0/18:0)	TBIL	-0.3942	0.0046^{**}
PC(16:0/18:0)	RDW	0.3758	0.0072^{**}
PC(16:0/18:0)	DBIL	-0.3541	0.0116^{*}
PC(16:0/18:0)	Creatinine	-0.3457	0.0139^{*}
PC(16:0/18:0)	Eosinophils	-0.3289	0.0197^{*}
PC(16:0/18:0)	α-HBDH	0.3468	0.0244^{*}
PC(16:0/18:0)	Monocytes	-0.2880	0.0425^{*}
PC(16:0/20:0)	PDW	-0.5390	0.0001^{***}
PC(16:0/20:0)	FIB	0.3792	0.0133^{*}

PC(16:0/20:0)	MPV	-0.3355	0.0172*
PC(16:0/20:0)	Ca	0.3243	0.0216*
PC(16:0/20:0)	RBC	-0.3023	0.0329*
PC(16:0/20:0)	ALP	-0.2952	0.0374*
PC(16:0/22:3)	CK-MB activity	0.3657	0.0172*
PC(16:0/22:3)	Eosinophils%	-0.3083	0.0294*
PC(16:0/22:3)	Eosinophils	-0.2966	0.0365*
PC(16:0e/16:0)	MPV	0.2979	0.0356*
PC(16:0e/16:0)	MCHC	-0.2972	0.0361*
PC(16:0e/16:0)	Monocytes%	0.2898	0.0412*
PC(16:0e/16:1)	Mg	-0.3121	0.0273*
PC(16:0e/16:1)	P	0.2809	0.0481*
PC(16:0e/18:0)	Glucose	0.3299	0.0193*
PC(16:0e/18:0)	Mg	-0.3212	0.0230*
PC(16:0e/18:0)	CO2	-0.3189	0.0349*
PC(16:0e/18:0)	IBIL	-0.2970	0.0362*
PC(16:0e/18:0)	Albumin	-0.2901	0.0410*
PC(16:0e/18:1)	Glucose	0.4196	0.0024**
PC(16:0e/18:1)	IBIL	-0.3868	0.0055**
PC(16:0e/18:1)	TBIL	-0.3698	0.0082**
PC(16:0e/18:1)	Eosinophils	-0.3393	0.0159*
PC(16:0e/18:1)	Mg	-0.3385	0.0162*
PC(16:0e/18:1)	DBIL	-0.3149	0.0259*
PC(16:0e/18:1)	CO2	-0.3341	0.0266*
PC(16:0e/18:1)	HCT	-0.2906	0.0407*
PC(16:0e/18:1)	HGB	-0.2803	0.0486*
PC(16:0e/20:2)	HCT	-0.4384	0.0015**
PC(16:0e/20:2)	HGB	-0.4361	0.0015**
PC(16:0e/20:2)	RBC	-0.4298	0.0018**
PC(16:0e/20:2)	Ca	0.4079	0.0033**
PC(16:0e/20:2)	Creatinine	-0.4062	0.0034**
PC(16:0e/20:2)	PDW	-0.3327	0.0182*
PC(16:0e/20:2)	Mg	-0.3181	0.0244*
PC(16:0e/20:2)	WBC	-0.3128	0.0270*
PC(16:0e/20:2)	Neutrophils	-0.3088	0.0291*
PC(16:0e/20:4)	P	0.3972	0.0043**
PC(16:0e/20:4)	Basophils	-0.3312	0.0188*
PC(16:0e/20:4)	ALT	0.3166	0.0251*
PC(16:0e/20:4)	Albumin	0.2892	0.0417*
PC(16:0p/16:0)	P	0.4172	0.0026**
PC(16:0p/16:0)	Mg	-0.2807	0.0483*
PC(16:0p/18:0)	HCT	-0.4439	0.0012**
PC(16:0p/18:0)	PDW	-0.4109	0.0030**
PC(16:0p/18:0)	HGB	-0.4040	0.0036**

PC(16:0p/18:0)	Glucose	0.3795	0.0066**
PC(16:0p/18:0)	RBC	-0.3667	0.0088**
PC(16:0p/18:0)	Mg	-0.3607	0.0101*
PC(16:0p/18:0)	Eosinophils	-0.3478	0.0133*
PC(16:0p/18:0)	TBIL	-0.3419	0.0151*
PC(16:0p/18:0)	DBIL	-0.3405	0.0155*
PC(16:0p/18:0)	IBIL	-0.3310	0.0189*
PC(16:0p/18:0)	Creatinine	-0.3262	0.0208*
PC(16:0p/18:0)	CO2	-0.3432	0.0226*
PC(16:0p/18:0)	Ca	0.2836	0.0459*
PC(16:0p/18:3)	Neutrophils	-0.3451	0.0141*
PC(16:0p/20:0)	HGB	0.2902	0.0409*
PC(16:0p/20:0)	PDW	0.2850	0.0449*
PC(16:0p/20:0)	Monocytes	0.2823	0.0470*
PC(16:0p/20:1)	Creatinine	-0.3783	0.0068**
PC(16:0p/20:1)	HGB	-0.3654	0.0091**
PC(16:0p/20:1)	RBC	-0.3626	0.0097**
PC(16:0p/20:1)	HCT	-0.3470	0.0136*
PC(16:0p/20:1)	Ca	0.3114	0.0277*
PC(16:0p/20:1)	Mg	-0.3072	0.0300*
PC(16:0p/20:1)	Neutrophils	-0.2900	0.0411*
PC(16:0p/20:1)	WBC	-0.2867	0.0435*
PC(18:0e/16:0)	Glucose	0.3492	0.0129*
PC(18:0e/16:0)	CO2	-0.3628	0.0155*
PC(18:0e/16:0)	Mg	-0.3191	0.0239*
PC(18:0e/16:0)	IBIL	-0.3158	0.0255*
PC(18:0e/16:0)	TBIL	-0.2792	0.0496*
PC(18:0e/18:0)	PDW	-0.4142	0.0028**
PC(18:0e/18:0)	HCT	-0.3669	0.0088**
PC(18:0e/18:0)	IBIL	-0.3612	0.0100*
PC(18:0e/18:0)	Glucose	0.3557	0.0112*
PC(18:0e/18:0)	Mg	-0.3533	0.0119*
PC(18:0e/18:0)	TBIL	-0.3490	0.0130*
PC(18:0e/18:0)	Creatinine	-0.3360	0.0171*
PC(18:0e/18:0)	DBIL	-0.3231	0.0221*
PC(18:0e/18:0)	HGB	-0.3022	0.0329*
PC(18:0e/18:0)	RBC	-0.2999	0.0343*
PC(18:0p/14:0)	Mg	-0.3351	0.0174*
PC(18:0p/14:0)	P	0.2971	0.0361*
PC(18:0p/16:0)	Glucose	0.3944	0.0046**
PC(18:0p/16:0)	CO2	-0.4007	0.0070**
PC(18:0p/16:0)	Eosinophils	-0.3739	0.0075**
PC(18:0p/16:0)	IBIL	-0.3688	0.0084**
PC(18:0p/16:0)	TBIL	-0.3634	0.0095**

PC(18:0p/16:0)	Mg	-0.3413	0.0153*
PC(18:0p/16:0)	DBIL	-0.3199	0.0235*
PC(18:0p/16:0)	HCT	-0.3096	0.0287*
PC(18:0p/16:0)	HGB	-0.3067	0.0303*
PC(18:0p/16:0)	Eosinophils%	-0.3000	0.0343*
PC(18:0p/18:0)	Monocytes	0.3441	0.0144*
PC(18:0p/18:0)	HGB	0.3226	0.0223*
PC(18:0p/18:1)	HCT	-0.5078	0.0002***
PC(18:0p/18:1)	Creatinine	-0.5063	0.0002***
PC(18:0p/18:1)	RBC	-0.5056	0.0002***
PC(18:0p/18:1)	HGB	-0.5029	0.0002***
PC(18:0p/18:1)	PDW	-0.4599	0.0008***
PC(18:0p/18:1)	DBIL	-0.3803	0.0064**
PC(18:0p/18:1)	Ca	0.3602	0.0102*
PC(18:0p/18:1)	MPV	-0.3314	0.0187*
PC(18:0p/18:1)	WBC	-0.3104	0.0282*
PC(18:0p/18:1)	Neutrophils	-0.3098	0.0286*
PC(18:0p/18:1)	TBIL	-0.3080	0.0295*
PC(18:0p/18:1)	Mg	-0.3041	0.0318*
PC(18:0p/18:1)	LDH	-0.2934	0.0386*
PC(18:0p/18:1)	Albumin	-0.2924	0.0394*
PC(18:0p/18:1)	UA	-0.2868	0.0434*
PC(18:0p/18:1)	CK-MB	-0.2854	0.0445*
PC(18:0p/18:1)	CO2	-0.2985	0.0491*
PC(18:1e/16:0)	Glucose	0.3983	0.0042**
PC(18:1e/16:0)	IBIL	-0.3832	0.0060**
PC(18:1e/16:0)	TBIL	-0.3751	0.0073**
PC(18:1e/16:0)	Eosinophils	-0.3695	0.0083**
PC(18:1e/16:0)	CO2	-0.3765	0.0118*
PC(18:1e/16:0)	Mg	-0.3443	0.0144*
PC(18:1e/16:0)	DBIL	-0.3322	0.0184*
PC(18:1e/16:0)	HCT	-0.3104	0.0282*
PC(18:1e/16:0)	HGB	-0.3086	0.0292*
PC(18:1e/16:0)	Eosinophils%	-0.2969	0.0363*
PC(18:1e/18:1)	PDW	-0.4851	0.0004***
PC(18:1e/18:1)	HCT	-0.4833	0.0004***
PC(18:1e/18:1)	RBC	-0.4328	0.0017**
PC(18:1e/18:1)	HGB	-0.4189	0.0025**
PC(18:1e/18:1)	Creatinine	-0.3947	0.0046**
PC(18:1e/18:1)	MPV	-0.3666	0.0088**
PC(18:1e/18:1)	Ca	0.3412	0.0153*
PC(18:1e/18:1)	ratio	-0.3199	0.0235*
PC(18:1e/18:1)	Neutrophils	-0.3188	0.0240*
PC(18:1e/18:1)	DBIL	-0.2957	0.0371*

PC(18:1e/18:1)	WBC	-0.2903	0.0408*
PC(18:1e/18:1)	LDH	-0.2896	0.0414*
PC(18:2e/12:0)	Eosinophils	-0.3468	0.0136*
PC(18:2e/12:0)	Eosinophils%	-0.3403	0.0156*
PE(16:0/18:0)	WBC	-0.3712	0.0080**
PE(16:0/18:0)	Neutrophils	-0.3451	0.0141*
PE(16:0/18:0)	Monocytes	-0.3128	0.0270*
PE(16:0/18:0)	ALP	-0.2876	0.0428**
PE(18:0/22:5)	IBIL	-0.4185	0.0025**
PE(18:0/22:5)	TBIL	-0.4084	0.0032**
PE(18:0/22:5)	Monocytes	-0.3812	0.0063**
PE(18:0/22:5)	Eosinophils	-0.3530	0.0119*
PE(18:0/22:5)	DBIL	-0.3514	0.0123*
PE(18:0/22:5)	CK.MB	-0.3267	0.0206*
PE(18:0/22:5)	WBC	-0.3248	0.0214*
PE(18:0/22:5)	Neutrophils	-0.3161	0.0253*
PE(18:0/22:5)	RDW	0.3142	0.0263*
PE(18:0/22:5)	RBC	-0.3037	0.0320*
PE(18:0/22:5)	Albumin	-0.2895	0.0414*
PE(18:0/22:5)	HGB	-0.2862	0.0439*
PE(18:0/22:5)	FIB	0.3111	0.0449*
PI(16:0/20:3)	RBC	-0.3926	0.0048**
PI(16:0/20:3)	HGB	-0.3561	0.0111*
PI(16:0/20:3)	HCT	-0.3082	0.0294*
PI(16:0/20:3)	Eosinophils	-0.2860	0.0441*
PI(16:0/20:3)	IBIL	-0.3401	0.0157*
PI(16:0/20:3)	TBIL	-0.3369	0.0167*
PI(16:0/20:3)	RDW	0.3065	0.0304*
PI(16:0/20:3)	Eosinophils	-0.2972	0.0361*
PI(16:0/20:3)	DBIL	-0.2967	0.0364*
SM(d18:1/18:2)	HCT	-0.5120	0.0001***
SM(d18:1/18:2)	RBC	-0.4855	0.0004***
SM(d18:1/18:2)	Creatinine	-0.4726	0.0005***
SM(d18:1/18:2)	HGB	-0.4537	0.0009***
SM(d18:1/18:2)	Ca	0.4357	0.0016**
SM(d18:1/18:2)	PDW	-0.4294	0.0019**
SM(d18:1/18:2)	Neutrophils	-0.3374	0.0166*
SM(d18:1/18:2)	WBC	-0.3099	0.0285*
SM(d18:1/18:3)	HCT	-0.3610	0.0100*
SM(d18:1/18:3)	Glucose	0.3527	0.0120*
SM(d18:1/18:3)	WBC	-0.2998	0.0344*
SM(d18:1/18:3)	TT	0.3206	0.0384*
SM(d18:1/18:3)	Mg	-0.2923	0.0394*
SM(d18:1/18:3)	Creatinine	-0.2825	0.0469*

SM(d18:1/18:3)	Lymphocytes	-0.2812	0.0479*
SM(d18:1/20:1)	MCHC	-0.3072	0.0300*
SM(d18:1/20:1)	Monocytes%	0.2818	0.0474*
SM(d18:1/20:4)	Neutrophils	-0.3604	0.0101*
SM(d18:1/20:5)	HCT	-0.3247	0.0214*
SM(d18:1/20:5)	WBC	-0.2886	0.0421*
SM(d18:1/20:5)	Neutrophils	-0.2843	0.0454*
SM(d18:1/22:0)	MCV	0.3142	0.0263*
SM(d18:1/22:0)	RBC	-0.3138	0.0265*
SM(d18:1/22:0)	MCHC	-0.2919	0.0397*
SM(d18:1/22:0)	r.GT	-0.2918	0.0398*
SM(d18:1/22:0)	HGB	-0.4025	0.0038**
SM(d18:1/22:0)	HCT	-0.3476	0.0134*
SM(d18:1/22:0)	RBC	-0.3399	0.0157*
SM(d18:1/22:0)	Eosinophils	-0.3342	0.0177*
SM(d18:1/22:0)	IBIL	-0.3315	0.0187*
SM(d18:1/22:0)	TBIL	-0.3280	0.0201*
SM(d18:1/22:0)	Monocytes	-0.3232	0.0221*
SM(d18:1/22:0)	DBIL	-0.3069	0.0302*
SM(d18:1/22:3)	TBIL	-0.4295	0.0019**
SM(d18:1/22:3)	IBIL	-0.4291	0.0019**
SM(d18:1/22:3)	DBIL	-0.3829	0.0061**
SM(d18:1/22:3)	HGB	-0.3635	0.0095**
SM(d18:1/22:3)	Eosinophils	-0.3294	0.0195*
SM(d18:1/22:3)	HCT	-0.3035	0.0321*
SM(d18:1/22:3)	RBC	-0.2879	0.0426*
SM(d18:1/22:4)	Neutrophils	-0.3343	0.0177*
SM(d18:1/22:4)	MCHC	-0.3096	0.0287*
TAG(48:1)	MPV	0.3640	0.0094**
TAG(48:1)	PDW	0.3291	0.0196*
TAG(48:1)	Creatinine	0.3084	0.0294*
TAG(50:2)	Eosinophils	0.2930	0.0389*

Abbreviation: ALP, alkaline phosphatase; ALT, alanine aminotransferase; APTT, activated partial thromboplastin time; AST, aspartate aminotransferase; BUN, blood urea nitrogen; Ca, calcium; CK-MB, creatine kinase-myocardial band; CRP, C-reactive protein; DAG, diacylglycerol; DBIL, direct bilirubin; FIB, FFA, free fatty acid; fibrinogen; HCs, healthy controls; IBIL, indirect bilirubin; INR, international normalized ratio; LDH, lactate dehydrogenase; LPC, lysoPhosphatidylcholine; LPE, lysoPhosphatidylethanolamine; M, moderate; MCH, mean corpuscular hemoglobin; MCHC, mean corpuscular hemoglobin concentration; MCV, mean corpuscular volume; Mg, magnesium; P, phosphorus; PC,

phosphatidylcholine; PCT, plateletocrit; PDW, platelet distribution width; PE, phosphatidylethanolamine; PT, prothrombin time; PI, phosphatidylinositol; RAs, recovered asymptomatic patients; RBC, red blood cell; RDW, red blood cell volume distribution width; RMs, recovered moderate patients; RSs, recovered critical and severe patients; S, critical and severe; SM, sphingomyelin; TAG, triacylglycerol; TBIL, total bilirubin; TT, thrombin time; UA, urine acid; WBC, white blood cell; α-HBDH, α-hydroxybutyrate dehydrogenase; γ-GT, γ-glutamyl transpeptidase; Statistical significance was set at 2-sided. *p<0.05, **p<0.01, ***p<0.001.

Table S9. Relationship between the differential lipids and clinical indices in RAs, RMs, RSs, and HCs.

Lipids	Clinical indices	HC		RA		RM		RS	
		Correlation coefficient	p-value	Correlation coefficient	p-value	Correlation coefficient	p-value	Correlation coefficient	p-value
DAG(14:0/16:0)	Neutrophils	-0.5101	0.0364*	0.1228	NS	0.1673	NS	0.3231	NS
DAG(14:0/18:0)	Neutrophils	-0.5506	0.0220*	-0.0402	NS	-0.0150	NS	0.1788	NS
DAG(14:0/18:2)	APTT	-0.7204	0.0011***	-0.4143	NS	-0.1769	NS	0.2441	NS
DAG(14:0/18:2)	P	-0.5849	0.0137*	-0.1299	NS	0.1842	NS	0.0215	NS
DAG(14:0/18:2)	CO2	-0.5110	0.0361*	0.0958	NS	-0.2848	NS	-0.0080	NS
DAG(16:0/16:0)	UA	0.6916	0.0021***	-0.2157	NS	-0.2555	NS	-0.1287	NS
DAG(16:0/16:1)	WBC	-0.5294	0.0289*	0.0836	NS	0.0277	NS	0.4161	0.0160*
DAG(16:0/16:1)	Neutrophils	-0.4868	0.0475*	0.0857	NS	0.0137	NS	0.3953	0.0228*
DAG(16:1/16:1)	BUN	-0.5064	0.0380*	0.0299	NS	-0.2663	NS	-0.0792	NS
DAG(16:1/18:0)	Basophils	0.5083	0.0372*	-0.1666	NS	-0.1060	NS	0.0340	NS
DAG(16:1/18:0)	Neutrophils	0.5171	0.0335*	-0.0200	NS	-0.1232	NS	0.1812	NS
DAG(16:1/18:0)	Monocytes	0.5477	0.0229*	-0.3617	NS	-0.1005	NS	0.2931	NS
DAG(16:1/18:0)	Creatinine	0.5511	0.0219*	-0.4099	NS	-0.0430	NS	-0.1406	NS
DAG(16:1/18:0)	CRP	0.5870	0.0132*	-0.2666	NS	-0.2638	NS	-0.3109	NS
DAG(16:1/18:0)	PLT	0.6349	0.0062***	-0.0426	NS	0.0580	NS	-0.2062	NS
DAG(16:1/18:0)	WBC	0.6527	0.0045***	-0.1503	NS	-0.1551	NS	0.1827	NS
DAG(18:1/20:0)	TT	-0.6416	0.0055***	-0.0889	NS	-0.2281	NS	0.0804	NS
DAG(18:1/20:0)	IBIL	0.5049	0.0387*	-0.3540	NS	0.0439	NS	0.0408	NS
DAG(18:1/20:0)	TBIL	0.5245	0.0307*	-0.3332	NS	0.0168	NS	-0.0125	NS
DAG(18:1/20:0)	DBIL	0.5521	0.0216*	-0.3223	NS	-0.0253	NS	-0.0700	NS
DAG(18:1/20:0)	r-GT	0.5584	0.0198*	-0.0775	NS	0.1083	NS	0.1297	NS

DAG(18:1/20:0)	AST	0.6252	0.0073***	-0.4311	NS	0.2582	NS	0.0188	NS
DAG(18:1/20:0)	ALT	0.6491	0.0048***	-0.3440	NS	0.1776	NS	-0.1382	NS
FFA(14:0)	FIB	0.5098	0.0366*	-0.1541	NS	-0.0881	NS	0.4204	0.0364*
FFA(16:2)	FIB	-0.5319	0.0280*	0.1417	NS	0.0138	NS	0.0355	NS
FFA(16:2)	Mg	-0.5296	0.0288*	-0.2749	NS	-0.2186	NS	-0.1315	NS
FFA(16:2)	HCT	0.5015	0.0403*	0.1538	NS	0.2662	NS	0.1299	NS
FFA(16:2)	BUN	0.5028	0.0397*	-0.2157	NS	-0.0466	NS	0.0724	NS
FFA(16:2)	HGB	0.5690	0.0171*	0.1664	NS	0.1792	NS	-0.0141	NS
FFA(16:2)	RBC	0.5849	0.0137*	0.0320	NS	0.2605	NS	0.0495	NS
FFA(20:3)	FIB	-0.5319	0.0280*	0.2347	NS	0.2181	NS	-0.3538	NS
FFA(22:3)	WBC	-0.5172	0.0335*	-0.1352	NS	-0.3158	NS	0.3546	0.0429*
FFA(22:3)	Mg	-0.5037	0.0393*	-0.0344	NS	-0.0949	NS	0.0970	NS
FFA(22:5)	Ca	0.5963	0.0115*	0.4225	NS	0.1634	NS	0.3085	NS
HexCer(d18:1/22:1)	IBIL	-0.5335	0.0274*	0.0991	NS	-0.2094	NS	-0.1287	NS
HexCer(d18:1/22:1)	TBIL	-0.5311	0.0283*	0.1491	NS	-0.2118	NS	-0.1573	NS
HexCer(d18:1/22:1)	AST	-0.4835	0.0493*	0.1042	NS	-0.1125	NS	-0.3248	NS
HexCer(d18:1/22:1)	PT	0.5493	0.0224*	0.2992	NS	0.0989	NS	0.3883	NS
HexCer(d18:1/22:1)	INR	0.5493	0.0224*	0.2992	NS	0.0989	NS	0.3883	NS
HexCer(d18:1/22:1)	Lymphocytes	0.5612	0.0191*	-0.0423	NS	0.3613	0.0422*	-0.1101	NS
HexCer(d18:1/22:1)	PLT	0.5906	0.0125*	-0.1539	NS	0.3033	NS	-0.1354	NS
LPC(15:0)	r-GT	-0.7085	0.0015***	0.0981	NS	-0.1995	NS	-0.2247	NS
LPC(15:0)	ALT	-0.7043	0.0016***	0.1281	NS	-0.2569	NS	-0.0947	NS
LPC(15:0)	Monocytes	-0.6552	0.0043***	-0.0176	NS	0.1702	NS	-0.2650	NS
LPC(15:0)	UA	-0.5028	0.0397*	0.1785	NS	-0.1287	NS	-0.1715	NS
LPC(15:0)	DBIL	-0.4969	0.0424*	-0.2924	NS	-0.2024	NS	-0.3958	0.0226*
LPC(15:0)	WBC	-0.4853	0.0483*	0.0588	NS	0.2244	NS	-0.2637	NS

LPC(16:0)	ALT	-0.7239	0.0010***	0.1839	NS	-0.1860	NS	0.1046	NS
LPC(16:0)	Monocytes	-0.6404	0.0056***	0.0643	NS	0.0178	NS	-0.1543	NS
LPC(16:0)	DBIL	-0.6160	0.0085***	-0.2696	NS	-0.1404	NS	-0.1880	NS
LPC(16:0)	Eosinophils	-0.6053	0.0100*	-0.0021	NS	0.2673	NS	-0.2182	NS
LPC(16:0)	r-GT	-0.6039	0.0102*	0.2552	NS	-0.0583	NS	0.1175	NS
LPC(16:0)	LDH	-0.5172	0.0335*	-0.4109	NS	-0.2228	NS	0.1281	NS
LPC(16:0)	AST	-0.5095	0.0367*	0.1130	NS	-0.2403	NS	0.0224	NS
LPC(16:0)	TBIL	-0.5074	0.0376*	-0.3197	NS	-0.0599	NS	-0.2182	NS
LPC(16:0)	CO2	-0.4880	0.0469*	-0.1932	NS	0.0541	NS	-0.0917	NS
LPC(16:1)	Monocytes	-0.6611	0.0039**	0.1172	NS	-0.0872	NS	-0.0397	NS
LPC(16:1)	ALT	-0.6464	0.0050**	0.4442	NS	-0.1058	NS	-0.0566	NS
LPC(16:1)	LDH	-0.5947	0.0118*	-0.3397	NS	-0.3218	NS	0.0383	NS
LPC(16:1)	Eosinophils	-0.5700	0.0169*	0.0901	NS	0.2575	NS	-0.2071	NS
LPC(16:1)	Neutrophils	-0.5190	0.0328*	0.2363	NS	-0.0224	NS	-0.0127	NS
LPC(16:1)	DBIL	-0.5107	0.0362*	-0.2469	NS	-0.1079	NS	-0.3697	0.0342*
LPC(16:1)	RDW	0.5531	0.0213*	0.0455	NS	-0.0619	NS	0.1916	NS
LPC(18:0)	Monocytes	-0.5765	0.0154*	0.0259	NS	0.0667	NS	-0.2464	NS
LPC(18:0)	DBIL	-0.5448	0.0237*	-0.3512	NS	-0.1164	NS	-0.2671	NS
LPC(18:0)	r-GT	-0.5154	0.0342*	0.1746	NS	-0.1571	NS	-0.0214	NS
LPC(18:0)	ALT	-0.5092	0.0368*	0.1663	NS	-0.1875	NS	0.0109	NS
LPC(18:0)	Neutrophils	-0.4917	0.0450*	0.2652	NS	0.0086	NS	-0.0079	NS
LPC(18:0)	α-HBDH	0.4862	0.0478*	0.0539	NS	-0.0675	NS	0.1602	NS
LPC(18:1)	Creatinine	-0.7060	0.0015**	-0.0787	NS	-0.2035	NS	-0.1609	NS
LPC(18:1)	ALT	-0.5696	0.0170*	0.1052	NS	-0.2643	NS	-0.0367	NS
LPC(18:1)	α-HBDH	0.5636	0.0185*	-0.2202	NS	-0.0703	NS	-0.0184	NS
LPC(20:0)	Neutrophils	-0.6033	0.0103*	-0.2074	NS	0.0713	NS	-0.1257	NS

LPC(20:0)	DBIL	-0.5706	0.0168*	-0.0579	NS	-0.2101	NS	-0.0826	NS
LPC(20:0)	r-GT	-0.5461	0.0233*	0.1612	NS	0.0046	NS	0.1800	NS
LPC(20:0)	Creatinine	-0.5074	0.0376*	0.0382	NS	0.0271	NS	-0.1487	NS
LPC(20:0)	TBIL	-0.4877	0.0470*	-0.1924	NS	-0.0222	NS	0.0762	NS
LPC(20:0)	α-HBDH	0.5451	0.0236*	-0.2040	NS	-0.0680	NS	-0.0189	NS
LPC(22:1)	Monocytes	-0.6491	0.0048**	-0.0933	NS	0.1687	NS	-0.2015	NS
LPC(22:1)	Neutrophils	-0.5874	0.0132*	0.0237	NS	-0.0328	NS	-0.1889	NS
LPC(22:1)	CRP	-0.5457	0.0235*	0.0424	NS	0.0068	NS	0.1784	NS
LPC(22:1)	WBC	-0.5074	0.0376*	-0.1352	NS	0.0711	NS	-0.3811	0.0286*
LPC(22:1)	HGB	-0.5003	0.0408*	0.0589	NS	-0.1463	NS	-0.1282	NS
LPC(22:1)	r-GT	-0.4932	0.0442*	0.1333	NS	-0.1789	NS	-0.1108	NS
LPC(22:1)	ALT	-0.4834	0.0493*	0.1467	NS	0.0250	NS	-0.0884	NS
LPC(22:1)	α-HBDH	0.5169	0.0336*	-0.0715	NS	-0.1135	NS	-0.0385	NS
LPC(O/16:0)	r-GT	-0.6568	0.0042**	0.1095	NS	-0.1440	NS	-0.1907	NS
LPC(O/16:0)	ALT	-0.6540	0.0044**	0.1529	NS	-0.2599	NS	-0.0668	NS
LPC(O/16:0)	Monocytes	-0.6331	0.0064**	0.0145	NS	0.1719	NS	-0.3236	NS
LPC(O/16:0)	Creatinine	-0.4975	0.0421*	0.2900	NS	-0.2068	NS	-0.3939	0.0233*
LPC(O/16:0)	UA	-0.4905	0.0456*	0.1434	NS	-0.1070	NS	-0.1875	NS
LPC(O/16:0)	CRP	-0.4893	0.0462*	0.2494	NS	0.1157	NS	0.0650	NS
LPC(O/18:0)	ALT	-0.7006	0.0017**	0.1271	NS	-0.2804	NS	-0.1911	NS
LPC(O/18:0)	DBIL	-0.6564	0.0042**	-0.2707	NS	-0.2723	NS	-0.2683	NS
LPC(O/18:0)	Monocytes	-0.5987	0.0111*	-0.0881	NS	0.0771	NS	-0.2135	NS
LPC(O/18:0)	AST	-0.5908	0.0125*	-0.0321	NS	-0.4095	0.0199*	-0.3852	0.0269*
LPC(O/18:0)	r-GT	-0.5658	0.0179*	0.0341	NS	-0.0594	NS	-0.3417	NS
LPC(O/18:0)	TBIL	-0.5319	0.0280 *	-0.4004	NS	-0.1126	NS	-0.2316	NS
LPC(O/18:0)	LDH	-0.4877	0.0470*	-0.7475	0.0004***	-0.3263	NS	-0.2124	NS

LPE(14:0)	ALT	-0.6074	0.0097**	0.5961	0.0090**	-0.1502	NS	0.2915	NS
LPE(14:0)	AST	-0.5255	0.0303*	0.3782	NS	-0.2213	NS	0.1893	NS
LPE(14:0)	Monocytes	-0.4954	0.0432*	0.4894	0.0393*	0.0059	NS	-0.0755	NS
LPE(20:3)	DBIL	-0.5914	0.0124*	-0.1756	NS	-0.2656	NS	-0.5148	0.0022**
LPE(20:3)	Eosinophils	-0.5770	0.0153*	-0.0186	NS	0.2869	NS	-0.2019	NS
LPE(20:3)	ALT	-0.5583	0.0199*	0.1798	NS	-0.2035	NS	0.0030	NS
LPE(20:3)	LDH	-0.5490	0.0225*	-0.3996	NS	-0.2529	NS	-0.1144	NS
LPE(20:3)	Monocytes	-0.5347	0.0270*	-0.0550	NS	-0.0369	NS	-0.2772	NS
LPE(20:3)	TBIL	-0.5147	0.0345*	-0.2587	NS	-0.1263	NS	-0.4713	0.0056**
LPE(20:4)	ALT	-0.7387	0.0007***	0.1663	NS	-0.1449	NS	0.0045	NS
LPE(20:4)	Monocytes	-0.6466	0.0050**	-0.1026	NS	0.0187	NS	-0.2123	NS
LPE(20:4)	LDH	-0.5686	0.0172*	-0.3975	NS	-0.2127	NS	-0.0314	NS
LPE(20:4)	DBIL	-0.5656	0.0180*	-0.1839	NS	-0.1206	NS	-0.5302	0.0015**
LPE(20:4)	Eosinophils	-0.5402	0.0252*	-0.1367	NS	0.2888	NS	-0.1152	NS
LPE(20:4)	r-GT	-0.5375	0.0261*	0.2252	NS	0.0015	NS	-0.0526	NS
LPE(20:4)	AST	-0.5059	0.0383*	0.1306	NS	-0.2387	NS	-0.1019	NS
LPE(20:4)	α-HBDH	0.5107	0.0362*	0.0352	NS	0.0193	NS	0.0990	NS
LPE(20:5)	DBIL	-0.6748	0.0030**	-0.2273	NS	-0.2011	NS	0.0475	NS
LPE(20:5)	ALT	-0.6331	0.0064**	0.2614	NS	-0.1146	NS	0.0301	NS
LPE(20:5)	Monocytes	-0.5814	0.0144*	-0.0301	NS	0.0147	NS	0.0596	NS
LPE(20:5)	AST	-0.5723	0.0164*	0.1959	NS	-0.2891	NS	0.0883	NS
LPE(20:5)	TBIL	-0.5515	0.0217*	-0.2711	NS	-0.0632	NS	-0.1063	NS
LPE(20:5)	r-GT	-0.5338	0.0273*	0.2851	NS	0.0270	NS	0.0687	NS
LPE(20:5)	LDH	-0.5294	0.0289*	-0.3882	NS	-0.3123	NS	0.2301	NS
LPE(20:5)	Eosinophils	-0.4923	0.0447*	-0.0684	NS	0.3024	NS	-0.0583	NS
LPE(20:5)	IBIL	-0.4902	0.0458	-0.2136	NS	-0.0125	NS	-0.1951	NS

LPE(22:6)	Monocytes	-0.5372	0.0262*	-0.0829	NS	0.0419	NS	-0.3104	NS
LPE(22:6)	r-GT	-0.4994	0.0413*	0.2789	NS	-0.1355	NS	-0.0137	NS
LPE(22:6)	α-HBDH	0.4886	0.0466*	0.0963	NS	0.0033	NS	0.2188	NS
MAG(22:5)	BUN	-0.5297	0.0287*	0.2343	NS	-0.0530	NS	0.1315	NS
MAG(22:5)	TT	-0.5296	0.0288*	-0.0558	NS	0.0669	NS	-0.1230	NS
MAG(22:5)	AST	0.5120	0.0356*	-0.1202	NS	0.0322	NS	-0.0633	NS
MAG(22:5)	FIB	0.5882	0.0130*	0.2823	NS	0.0299	NS	0.2355	NS
PA(16:0/18:0)	HCT	-0.4896	0.0461*	0.2695	NS	0.1331	NS	-0.1247	NS
PC(14:0/16:0)	Monocytes	-0.5950	0.0118*	0.2644	NS	0.1388	NS	-0.0701	NS
PC(14:0/16:0)	CRP	-0.5469	0.0231*	0.3187	NS	0.2505	NS	-0.0354	NS
PC(14:0/16:0)	Eosinophils	-0.5095	0.0367*	-0.0031	NS	0.4621	0.0077**	-0.0282	NS
PC(16:0/18:0)	Monocytes	-0.7314	0.0008***	-0.3961	NS	0.0130	NS	-0.1778	NS
PC(16:0/18:0)	DBIL	-0.6196	0.0080**	-0.4514	NS	-0.0527	NS	-0.1765	NS
PC(16:0/18:0)	AST	-0.6055	0.0100*	-0.4415	NS	0.2762	NS	-0.1298	NS
PC(16:0/18:0)	Neutrophils	-0.5776	0.0152*	-0.1992	NS	0.0092	NS	-0.3583	0.0406*
PC(16:0/18:0)	ALT	-0.5546	0.0209*	-0.5124	0.0297*	0.0064	NS	-0.0766	NS
PC(16:0/18:0)	TBIL	-0.5343	0.0271*	-0.5618	0.0152*	-0.0218	NS	-0.2055	NS
PC(16:0/18:0)	r-GT	-0.5277	0.0295*	-0.1839	NS	0.0561	NS	-0.1178	NS
PC(16:0/18:0)	WBC	-0.4926	0.0445*	-0.3251	NS	0.0288	NS	-0.3105	NS
PC(16:0/18:0)	CO2	-0.4856	0.0482*	-0.0031	NS	-0.1155	NS	0.3850	0.0473*
PC(16:0/18:0)	IBIL	-0.4853	0.0483*	-0.6244	0.0056**	0.0303	NS	-0.2611	NS
PC(16:0/18:0)	α-HBDH	0.5255	0.0303*	-0.1657	NS	0.1123	NS	0.2107	NS
PC(16:0/18:0)	RDW	0.6317	0.0065**	-0.0528	NS	-0.0605	NS	0.1897	NS
PC(16:0/20:0)	WBC	0.4853	0.0483*	0.1765	NS	0.1716	NS	-0.1126	NS
PC(16:0/20:0)	Mg	0.5111	0.0360*	0.0062	NS	-0.1229	NS	-0.0748	NS
PC(16:0/20:3)	Albumin	0.6021	0.0105*	0.4058	NS	-0.0640	NS	0.0855	NS

PC(16:0/22:3)	RBC	-0.6658	0.0035**	-0.2219	NS	0.2541	NS	0.2584	NS
PC(16:0/22:3)	ALT	-0.5374	0.0261*	-0.3481	NS	0.0376	NS	0.0780	NS
PC(16:0/22:3)	Glucose	-0.5089	0.0370*	-0.1662	NS	0.0824	NS	0.3497	0.0461*
PC(16:0/22:3)	HGB	-0.5089	0.0370*	-0.3276	NS	0.1511	NS	0.2196	NS
PC(16:0/22:3)	HCT	-0.4868	0.0475*	-0.3148	NS	0.2456	NS	0.1683	NS
PC(16:0/22:3)	Mg	0.6798	0.0027**	0.2978	NS	0.0102	NS	-0.0708	NS
PC(16:0e/16:0)	Neutrophils	-0.5739	0.0160*	0.0918	NS	0.1751	NS	-0.0045	NS
PC(16:0e/18:0)	DBIL	-0.6344	0.0062**	-0.3378	NS	-0.2745	NS	-0.0035	NS
PC(16:0e/18:0)	ALT	-0.6123	0.0090**	0.0599	NS	-0.1289	NS	-0.0174	NS
PC(16:0e/18:0)	AST	-0.5662	0.0178*	-0.1699	NS	-0.3272	NS	-0.0236	NS
PC(16:0e/18:0)	TBIL	-0.5368	0.0263*	-0.4904	0.0388*	-0.1861	NS	-0.0953	NS
PC(16:0e/18:0)	r-GT	-0.4957	0.0430*	-0.0775	NS	0.0370	NS	-0.0962	NS
PC(16:0e/18:0)	IBIL	-0.4926	0.0445*	-0.4964	0.0361*	-0.1538	NS	-0.1362	NS
PC(16:0e/18:0)	PT	0.6183	0.0082**	-0.1018	NS	0.1875	NS	0.1874	NS
PC(16:0e/18:0)	INR	0.6183	0.0082**	-0.1018	NS	0.1875	NS	0.1874	NS
PC(16:0e/20:2)	Ca	0.5656	0.0180*	-0.2448	NS	0.2572	NS	0.3188	NS
PC(16:0e/20:4)	PT	0.6009	0.0107*	-0.0457	NS	0.0517	NS	-0.0004	NS
PC(16:0e/20:4)	INR	0.6009	0.0107*	-0.0457	NS	0.0517	NS	-0.0004	NS
PC(16:0p/16:0)	APTT	0.5052	0.0386*	-0.0196	NS	-0.1128	NS	-0.1559	NS
PC(16:0p/16:0)	PT	0.5090	0.0369*	-0.1817	NS	-0.1361	NS	0.2380	NS
PC(16:0p/16:0)	INR	0.5090	0.0369*	-0.1817	NS	-0.1361	NS	0.2380	NS
PC(16:0p/18:0)	Eosinophils	-0.5807	0.0145*	0.0818	NS	0.2919	NS	-0.0915	NS
PC(16:0p/18:0)	ALT	-0.5558	0.0205*	0.4339	NS	-0.1230	NS	0.1461	NS
PC(16:0p/18:1)	WBC	-0.7598	0.0004***	0.0010	NS	-0.0328	NS	0.3072	NS
PC(16:0p/18:1)	Monocytes	-0.6183	0.0082**	0.1649	NS	0.1105	NS	0.1661	NS
PC(16:0p/18:1)	Lymphocytes	-0.6021	0.0105*	0.0464	NS	-0.1441	NS	0.0211	NS

PC(16:0p/18:1)	TT	0.5874	0.0132*	0.2253	NS	-0.1233	NS	0.0541	NS
PC(16:0p/22:6)	DBIL	-0.7239	0.0010**	0.0207	NS	-0.1019	NS	-0.0381	NS
PC(16:0p/22:6)	TBIL	-0.6985	0.0018**	-0.0911	NS	-0.1749	NS	-0.2005	NS
PC(16:0p/22:6)	ALT	-0.6883	0.0023**	0.0640	NS	-0.0850	NS	0.1250	NS
PC(16:0p/22:6)	IBIL	-0.6544	0.0044**	-0.0774	NS	-0.1843	NS	-0.2828	NS
PC(16:0p/22:6)	AST	-0.6031	0.0104*	-0.0238	NS	-0.2079	NS	0.0382	NS
PC(16:0p/22:6)	r-GT	-0.5732	0.0162*	0.0031	NS	0.0714	NS	0.0476	NS
PC(16:0p/22:6)	PT	0.5550	0.0208*	0.0498	NS	0.3202	NS	0.1407	NS
PC(16:0p/22:6)	INR	0.5550	0.0208*	0.0498	NS	0.3202	NS	0.1407	NS
PC(18:0e/16:0)	ALT	-0.6687	0.0033**	0.0517	NS	-0.0784	NS	-0.0167	NS
PC(18:0e/16:0)	AST	-0.6080	0.0096**	-0.1337	NS	-0.2957	NS	-0.0310	NS
PC(18:0e/16:0)	DBIL	-0.5914	0.0124*	-0.2872	NS	-0.3169	NS	-0.0070	NS
PC(18:0e/16:0)	r-GT	-0.5141	0.0347*	-0.0103	NS	0.0526	NS	-0.1404	NS
PC(18:0e/16:0)	TBIL	-0.5098	0.0366*	-0.4294	NS	-0.1952	NS	-0.1066	NS
PC(18:0e/16:0)	PT	0.6059	0.0099**	-0.1267	NS	0.2442	NS	0.1959	NS
PC(18:0e/16:0)	INR	0.6059	0.0099**	-0.1267	NS	0.2442	NS	0.1959	NS
PC(18:0e/18:0)	ALT	-0.5546	0.0209*	0.2273	NS	0.0825	NS	0.4383	0.0107*
PC(18:0e/18:0)	RBC	-0.4856	0.0482*	0.1538	NS	0.2264	NS	-0.2396	NS
PC(18:0p/16:0)	Eosinophils	-0.6568	0.0042**	-0.1523	NS	0.3292	NS	-0.1282	NS
PC(18:0p/16:0)	ALT	-0.5914	0.0124*	0.2293	NS	-0.1221	NS	0.2231	NS
PC(18:0p/18:0)	TBIL	-0.4902	0.0458*	-0.0217	NS	-0.4407	0.0116*	-0.0234	NS
PC(18:0p/18:0)	IBIL	-0.4853	0.0483*	-0.0877	NS	-0.3933	0.0260*	-0.0510	NS
PC(18:0p/18:1)	ALT	-0.7106	0.0014**	-0.0769	NS	-0.2217	NS	-0.0768	NS
PC(18:0p/18:1)	AST	-0.6144	0.0087**	-0.0401	NS	-0.3119	NS	-0.1059	NS
PC(18:0p/18:1)	LDH	-0.5435	0.0241*	-0.0885	NS	-0.2511	NS	-0.2448	NS
PC(18:0p/18:1)	Eosinophils	-0.5022	0.0399*	0.0432	NS	0.1836	NS	-0.1028	NS

PC(18:1e/16:0)	Eosinophils	-0.5120	0.0356*	-0.0880	NS	0.3022	NS	-0.1628	NS
PC(18:1e/18:1)	LDH	-0.4847	0.0486*	-0.0129	NS	0.0765	NS	-0.2620	NS
PC(18:1e/18:1)	globulin	0.5237	0.0310*	-0.1116	NS	-0.0620	NS	0.1190	NS
PC(18:2/20:3)	α-HBDH	-0.5071	0.0378*	-0.1937	NS	-0.1467	NS	0.0293	NS
PC(18:2/20:3)	CK-MB	0.4853	0.0483*	0.1723	NS	0.0006	NS	-0.3034	NS
PC(18:2/20:3)	ALT	0.5117	0.0358*	-0.4793	0.0441*	0.1763	NS	-0.1689	NS
PC(18:2/20:3)	Creatinine	0.5368	0.0263*	0.1063	NS	-0.3765	0.0337*	-0.2856	NS
PC(18:2e/12:0)	HCT	-0.6278	0.0070**	-0.2405	NS	-0.1005	NS	0.1464	NS
PC(18:2e/12:0)	HGB	-0.6094	0.0094**	-0.2398	NS	-0.3816	0.0311*	0.0373	NS
PC(18:2e/12:0)	RBC	-0.6082	0.0096**	0.0464	NS	-0.2814	NS	-0.0217	NS
PC(18:2e/12:0)	Eosinophils	-0.4997	0.0411*	-0.5945	0.0093**	-0.0035	NS	-0.2919	NS
PC(18:2e/12:0)	TT	0.4889	0.0464*	0.0021	NS	0.0777	NS	-0.2274	NS
PC(18:2e/12:0)	Ca	0.6061	0.0099**	0.1457	NS	0.0850	NS	-0.3110	NS
PC(18:2e/16:0)	Lymphocytes	-0.6291	0.0068**	0.0052	NS	-0.1615	NS	-0.0064	NS
PC(18:2e/16:0)	Mg	-0.6047	0.0101*	0.1281	NS	-0.1702	NS	0.0453	NS
PC(18:2e/16:0)	WBC	-0.5735	0.0161*	-0.0464	NS	-0.0623	NS	0.3212	NS
PC(18:2e/16:0)	FIB	-0.4902	0.0458*	-0.3237	NS	0.0750	NS	-0.1175	NS
PC(18:2p/20:4)	DBIL	-0.7350	0.0008***	0.0568	NS	-0.1198	NS	-0.0418	NS
PC(18:2p/20:4)	TBIL	-0.7132	0.0013**	-0.0445	NS	-0.1652	NS	-0.2052	NS
PC(18:2p/20:4)	ALT	-0.7092	0.0014**	0.0733	NS	-0.0957	NS	0.1171	NS
PC(18:2p/20:4)	IBIL	-0.6765	0.0029**	-0.0279	NS	-0.1667	NS	-0.2925	NS
PC(18:2p/20:4)	AST	-0.6486	0.0049**	-0.0218	NS	-0.2167	NS	0.0263	NS
PC(18:2p/20:4)	r-GT	-0.5732	0.0162*	0.0155	NS	0.0471	NS	0.0627	NS
PC(18:2p/20:4)	PT	0.5612	0.0191*	0.0426	NS	0.3319	NS	0.0962	NS
PC(18:2p/20:4)	INR	0.5612	0.0191*	0.0426	NS	0.3319	NS	0.0962	NS
PE(16:0/18:0)	Mg	0.5283	0.0292*	0.2416	NS	-0.1347	NS	-0.1008	NS

PE(16:0/22:4)	CK-MB	-0.7457	0.0006***	-0.1998	NS	-0.0440	NS	-0.1237	NS
PE(16:0/22:4)	HGB	-0.5351	0.0269*	-0.3758	NS	-0.1406	NS	-0.2025	NS
PE(16:0/22:4)	HCT	-0.5245	0.0307*	-0.3434	NS	0.0486	NS	-0.0852	NS
PE(18:0/22:5)	Eosinophils	-0.6683	0.0034**	-0.0581	NS	-0.0423	NS	-0.2225	NS
PE(18:0/22:5)	LDH	-0.5043	0.0390*	-0.3207	NS	-0.0241	NS	-0.0207	NS
PE(18:0/22:5)	RDW	0.7678	0.0003***	-0.0467	NS	0.0035	NS	0.1010	NS
PI(16:0/20:3)	RBC	-0.5663	0.0178*	0.2745	NS	0.0579	NS	-0.2900	NS
PI(16:0/20:3)	HCT	-0.5491	0.0224*	0.0298	NS	-0.0461	NS	-0.2261	NS
PI(18:0/20:3)	LDH	-0.5807	0.0145*	0.0073	NS	-0.0389	NS	0.2569	NS
PI(18:0/20:3)	BUN	-0.4929	0.0444*	-0.1304	NS	0.2690	NS	0.1530	NS
PI(18:0/20:3)	PT	0.4965	0.0426*	0.1605	NS	-0.2559	NS	-0.3229	NS
PI(18:0/20:3)	INR	0.4965	0.0426*	0.1605	NS	-0.2559	NS	-0.3229	NS
PI(18:0/20:3)	ALP	0.4994	0.0412*	-0.1878	NS	0.1169	NS	0.0309	NS
PI(18:0/20:3)	Mg	0.5112	0.0360*	0.1662	NS	0.1471	NS	0.0486	NS
PI(18:0/20:3)	RDW	0.5184	0.0330*	-0.2929	NS	-0.2970	NS	0.1548	NS
SM(d18:1/16:0)	TBIL	-0.5288	0.0291*	-0.0494	NS	-0.1840	NS	0.0938	NS
SM(d18:1/16:0)	Glucose	-0.5208	0.0321*	-0.3225	NS	0.1476	NS	-0.1209	NS
SM(d18:1/16:0)	IBIL	-0.4975	0.0422*	-0.1477	NS	-0.1986	NS	0.0471	NS
SM(d18:1/16:0)	ALT	-0.4953	0.0432*	-0.0779	NS	-0.1627	NS	0.0576	NS
SM(d18:1/18:2)	AST	0.5143	0.0347*	0.0228	NS	0.0370	NS	-0.2300	NS
SM(d18:1/18:2)	Ca	0.5506	0.0220*	-0.3532	NS	-0.1678	NS	0.3516	0.0448*
SM(d18:1/18:3)	Lymphocytes	-0.6387	0.0058**	-0.1992	NS	-0.0480	NS	-0.0316	NS
SM(d18:1/18:3)	HCT	-0.6270	0.0071**	0.1269	NS	-0.2071	NS	-0.2098	NS
SM(d18:1/18:3)	RBC	-0.6196	0.0080**	0.1393	NS	-0.4239	0.0156*	-0.0237	NS
SM(d18:1/18:3)	HGB	-0.5632	0.0186*	0.1116	NS	-0.4175	0.0174*	-0.0820	NS
SM(d18:1/18:3)	Monocytes	-0.5283	0.0293*	-0.0632	NS	0.2497	NS	-0.0274	NS

SM(d18:1/18:3)	r-GT	-0.5102	0.0364*	0.1643	NS	-0.3820	0.0310	-0.0664	NS
SM(d18:1/18:3)	WBC	-0.4966	0.0426*	-0.2487	NS	0.0862	NS	-0.0943	NS
SM(d18:1/18:3)	TT	0.5213	0.0319*	0.1302	NS	-0.1016	NS	0.1609	NS
SM(d18:1/18:3)	ALP	0.5519	0.0216*	-0.0010	NS	-0.0029	NS	-0.0987	NS
SM(d18:1/18:4)	DBIL	-0.7097	0.0014**	-0.1957	NS	-0.2915	NS	0.0631	NS
SM(d18:1/18:4)	TBIL	-0.6192	0.0080**	-0.3318	NS	-0.3213	NS	-0.1244	NS
SM(d18:1/18:4)	r-GT	-0.6079	0.0096**	0.0435	NS	-0.3712	0.0365*	0.1235	NS
SM(d18:1/18:4)	IBIL	-0.5602	0.0193*	-0.3237	NS	-0.3313	NS	-0.2234	NS
SM(d18:1/18:4)	Monocytes	-0.5052	0.0386*	-0.1247	NS	0.0458	NS	0.0067	NS
SM(d18:1/18:4)	Neutrophils	-0.4991	0.0414*	0.1458	NS	-0.0372	NS	0.0767	NS
SM(d18:1/20:1)	Ca	0.5853	0.0136*	0.0341	NS	-0.0732	NS	-0.0694	NS
SM(d18:1/20:2)	Ca	0.7153	0.0012**	0.0124	NS	0.0653	NS	-0.0085	NS
SM(d18:1/20:4)	r-GT	-0.5892	0.0128*	-0.1756	NS	-0.0337	NS	-0.1123	NS
SM(d18:1/20:4)	ALT	-0.5215	0.0318*	-0.2231	NS	-0.1398	NS	-0.1056	NS
SM(d18:1/20:4)	Mg	0.5185	0.0330*	0.2967	NS	0.0745	NS	0.0849	NS
SM(d18:1/20:5)	CRP	-0.6094	0.0094**	0.4689	0.0496*	-0.2619	NS	0.0931	NS
SM(d18:1/22:0)	Monocytes	-0.6921	0.0021**	0.6159	0.0065**	0.0753	NS	-0.2187	NS
SM(d18:1/22:0)	Albumin	-0.6193	0.0080**	-0.3098	NS	-0.2211	NS	0.1057	NS
SM(d18:1/22:0)	HGB	-0.6143	0.0087**	0.3008	NS	-0.2077	NS	-0.2340	NS
SM(d18:1/22:0)	Eosinophils	-0.5795	0.0148*	0.5251	0.0252*	0.4042	0.0218*	-0.1518	NS
SM(d18:1/22:0)	HCT	-0.5653	0.0180*	0.3292	NS	-0.0374	NS	-0.2075	NS
SM(d18:1/22:0)	RDW	0.5170	0.0336*	-0.1574	NS	0.0820	NS	-0.0971	NS
SM(d18:1/22:3)	Monocytes	-0.6564	0.0042**	0.3339	NS	0.0360	NS	-0.1633	NS
SM(d18:1/22:3)	Eosinophils	-0.6126	0.0089**	0.4516	NS	0.3101	NS	-0.1883	NS
SM(d18:1/22:3)	HGB	-0.5690	0.0171*	0.2036	NS	-0.1079	NS	-0.2027	NS
SM(d18:1/22:3)	ALT	-0.5485	0.0226*	0.5207	0.0267*	-0.0516	NS	0.1948	NS

SM(d18:1/22:3)	HCT	-0.5322	0.0279*	0.1909	NS	0.0519	NS	-0.1635	NS
SM(d18:1/22:3)	Albumin	-0.5003	0.0408*	-0.3882	NS	-0.1581	NS	-0.0345	NS
SM(d18:1/22:3)	RBC	-0.4844	0.0488*	0.2116	NS	-0.0174	NS	-0.1444	NS
SM(d18:1/22:3)	ALP	0.4920	0.0448*	0.2602	NS	0.2857	NS	-0.0460	NS
SM(d18:1/22:3)	RDW	0.6219	0.0077**	-0.2754	NS	0.0820	NS	-0.0895	NS
SM(d18:1/22:4)	Mg	0.5495	0.0223*	0.3203	NS	0.1032	NS	0.0995	NS
TAG(50:0)	APTT	-0.5289	0.0290*	-0.2235	NS	0.2111	NS	0.1576	NS
TAG(50:0)	r-GT	0.5019	0.0401*	-0.3230	NS	0.3059	NS	-0.1860	NS
TAG(50:0)	UA	0.5376	0.0260*	-0.2092	NS	-0.0477	NS	-0.2390	NS
TAG(50:0)	ALT	0.5702	0.0168*	-0.0552	NS	0.0262	NS	-0.2210	NS
TAG(50:2)	CK-MB	-0.5150	0.0344*	-0.2701	NS	-0.0999	NS	-0.0074	NS
TAG(52:5)	LDH	-0.4876	0.0471*	-0.1644	NS	0.0950	NS	-0.0861	NS
DAG(14:0/16:0)	Mg	-0.1810	NS	0.6008	0.0084**	-0.1116	NS	0.4230	0.0142*
DAG(14:0/16:0)	Glucose	-0.1545	NS	0.4923	0.0380*	-0.1796	NS	-0.0364	NS
DAG(14:0/18:0)	Glucose	-0.1803	NS	0.6574	0.0030**	-0.0923	NS	-0.0219	NS
DAG(14:0/18:0)	RDW	0.0333	NS	0.4886	0.0396*	-0.0555	NS	0.3081	NS
DAG(14:0/18:0)	CK-MB	0.2745	NS	-0.6326	0.0048**	0.0180	NS	-0.0739	NS
DAG(14:0/18:2)	Basophils	-0.2983	NS	0.4783	0.0447*	0.1510	NS	0.1483	NS
DAG(14:0/18:2)	ALT	-0.0974	NS	0.7423	0.0004***	-0.0838	NS	0.0418	NS
DAG(14:0/18:2)	AST	-0.0275	NS	0.6769	0.0020**	-0.1884	NS	0.0729	NS
DAG(14:0/18:2)	RBC	0.2259	NS	0.5200	0.0270*	-0.0149	NS	-0.3379	NS
DAG(16:0/16:0)	ALT	0.1926	NS	-0.5083	0.0313*	0.0108	NS	-0.2227	NS
DAG(16:0/16:0)	PT	0.3389	NS	-0.4704	0.0488*	0.1029	NS	-0.1399	NS
DAG(16:0/16:0)	INR	0.3389	NS	-0.4704	0.0488*	0.1029	NS	-0.1399	NS
DAG(16:0/16:1)	Mg	-0.2993	NS	0.5018	0.0338*	-0.1463	NS	0.2396	NS
DAG(16:0/16:1)	Glucose	-0.2109	NS	0.6305	0.0050**	-0.0008	NS	-0.0162	NS

DAG(16:0/16:1)	RDW	0.1024	NS	0.6035	0.0080**	-0.0587	NS	0.2718	NS
DAG(16:0/16:1)	CK-MB	0.1838	NS	-0.5893	0.0101*	0.0056	NS	0.0192	NS
DAG(16:1/16:1)	Glucose	-0.1496	NS	0.5934	0.0094**	0.1366	NS	-0.1270	NS
DAG(16:1/16:1)	PT	0.1316	NS	-0.5016	0.0339*	0.3994	0.0235*	0.1584	NS
DAG(16:1/16:1)	INR	0.1316	NS	-0.5016	0.0339*	0.3994	0.0235*	0.1584	NS
DAG(16:1/16:1)	CK-MB	0.1642	NS	-0.5831	0.0111*	-0.1499	NS	-0.2262	NS
DAG(16:1/18:0)	UA	0.2211	NS	-0.5676	0.0140*	0.1733	NS	-0.1175	NS
DAG(18:1/20:0)	Glucose	-0.0527	NS	0.5893	0.0101*	-0.1222	NS	0.0433	NS
DAG(18:1/20:0)	CK-MB	0.2647	NS	-0.5562	0.0165*	0.2449	NS	0.1551	NS
FFA(14:0)	ALP	-0.3350	NS	0.4863	0.0407*	-0.1511	NS	-0.2216	NS
FFA(20:3)	CK-MB	-0.2868	NS	0.5480	0.0186*	-0.2045	NS	0.1555	NS
FFA(20:4)	RDW	-0.0802	NS	0.4979	0.0355*	0.1043	NS	0.2307	NS
FFA(20:4)	Glucose	-0.0723	NS	0.4819	0.0428*	0.0148	NS	-0.2937	NS
FFA(20:5)	RDW	-0.0728	NS	0.4803	0.0436*	0.0861	NS	0.2444	NS
FFA(20:5)	Glucose	-0.0503	NS	0.4861	0.0408*	-0.0244	NS	-0.3176	NS
FFA(22:5)	CO2	0.1340	NS	0.6136	0.0068**	0.0403	NS	-0.0883	NS
LPC(15:0)	LDH	-0.3456	NS	-0.6815	0.0018**	-0.3572	NS	-0.1789	NS
LPC(18:0)	LDH	-0.3873	NS	-0.5142	0.0290*	-0.3258	NS	0.0064	NS
LPC(18:1)	LDH	-0.0178	NS	-0.5368	0.0216*	-0.4149	0.0281*	0.0158	NS
LPC(20:0)	P	0.2880	NS	-0.5372	0.0215*	0.2454	NS	-0.1109	NS
LPC(O/16:0)	LDH	-0.3725	NS	-0.6071	0.0075**	-0.3534	NS	-0.2522	NS
LPE(14:0)	WBC	-0.3235	NS	0.4985	0.0353*	0.2980	NS	0.0023	NS
LPE(14:0)	CRP	-0.2759	NS	0.5370	0.0216*	0.4826	0.0051**	0.3723	0.0329*
LPE(14:0)	r-GT	-0.2239	NS	0.4773	0.0452*	-0.0298	NS	0.0620	NS
LPE(14:0)	TT	0.1441	NS	0.5189	0.0274*	0.2815	NS	-0.2808	NS
MAG(22:5)	Glucose	-0.3102	NS	0.6471	0.0037**	-0.4065	0.0318*	0.1931	NS

PA(16:0/18:0)	globulin	-0.0221	NS	0.5085	0.0312*	-0.0518	NS	-0.0570	NS
PA(16:0/18:0)	PT	0.3926	NS	-0.5465	0.0189*	0.2344	NS	-0.1777	NS
PA(16:0/18:0)	INR	0.3926	NS	-0.5465	0.0189*	0.2344	NS	-0.1777	NS
PC(14:0/16:0)	Neutrophils	-0.1741	NS	0.6326	0.0048**	0.2143	NS	-0.0659	NS
PC(16:0/18:0)	Creatinine	-0.4363	NS	-0.4943	0.0370*	0.2055	NS	-0.2860	NS
PC(16:0/18:0)	CK-MB	-0.1471	NS	-0.4902	0.0389*	0.3344	NS	-0.1590	NS
PC(16:0/18:0)	UA	-0.1447	NS	-0.4923	0.0380*	0.1763	NS	-0.4398	0.0104*
PC(16:0/18:0)	Basophils	-0.1129	NS	-0.7508	0.0003***	0.2315	NS	-0.0108	NS
PC(16:0/20:3)	Mg	-0.0998	NS	0.5581	0.0161*	-0.0390	NS	-0.1119	NS
PC(16:0/20:3)	TP	0.1815	NS	0.4744	0.0467*	-0.0900	NS	-0.1017	NS
PC(16:0/22:3)	AST	-0.1932	NS	-0.4746	0.0466*	0.0978	NS	0.0685	NS
PC(16:0/22:3)	Ca	0.3816	NS	0.5186	0.0275*	-0.1810	NS	-0.1782	NS
PC(16:0e/16:0)	TBIL	-0.4167	NS	-0.5049	0.0326*	-0.2042	NS	0.1574	NS
PC(16:0e/16:0)	IBIL	-0.3873	NS	-0.5129	0.0295*	-0.1586	NS	0.0702	NS
PC(16:0e/16:0)	LDH	0.0025	NS	-0.6773	0.0020**	-0.0208	NS	0.1664	NS
PC(16:0e/16:1)	Neutrophils	-0.0196	NS	0.5562	0.0165*	-0.3710	0.0366*	-0.1008	NS
PC(16:0e/16:1)	CRP	0.0135	NS	0.5256	0.0251*	-0.3385	NS	0.0923	NS
PC(16:0e/16:1)	WBC	0.1422	NS	0.6264	0.0054**	-0.3448	NS	-0.0993	NS
PC(16:0e/18:0)	LDH	-0.2157	NS	-0.6608	0.0028**	-0.2956	NS	0.0416	NS
PC(16:0e/18:0)	PLT	0.0907	NS	0.5803	0.0116*	0.2992	NS	-0.0939	NS
PC(16:0e/18:1)	LDH	0.0588	NS	-0.5421	0.0201*	-0.2039	NS	0.0050	NS
PC(16:0e/18:1)	APTT	0.2269	NS	-0.4760	0.0459*	0.0066	NS	-0.2232	NS
PC(16:0e/20:2)	globulin	-0.1496	NS	0.6839	0.0017**	-0.0492	NS	0.1543	NS
PC(16:0p/16:0)	AST	-0.0529	NS	0.4777	0.0450*	-0.2834	NS	0.1139	NS
PC(16:0p/16:0)	Eosinophils	0.1866	NS	0.5479	0.0186*	-0.1035	NS	-0.0321	NS
PC(16:0p/16:0)	r-GT	0.1980	NS	0.6012	0.0083**	-0.2361	NS	0.1270	NS

PC(16:0p/16:0)	CRP	0.2219	NS	0.4853	0.0412*	-0.4304	0.0139*	0.2930	NS
PC(16:0p/16:0)	ALT	0.3411	NS	0.4804	0.0436*	-0.1447	NS	0.0601	NS
PC(16:0p/18:1)	r-GT	-0.3456	NS	0.4845	0.0416*	-0.2996	NS	-0.0548	NS
PC(16:0p/18:1)	ALT	-0.2834	NS	0.5981	0.0087**	-0.1144	NS	0.0949	NS
PC(16:0p/18:1)	AST	-0.0443	NS	0.5554	0.0167*	-0.3022	NS	0.0561	NS
PC(16:0p/18:3)	Ca	0.1411	NS	0.5010	0.0342*	0.1223	NS	0.0037	NS
PC(16:0p/20:0)	TT	0.2291	NS	-0.4920	0.0381*	0.0579	NS	-0.0456	NS
PC(16:0p/20:1)	globulin	-0.2502	NS	0.5031	0.0333*	0.1840	NS	-0.0043	NS
PC(16:0p/20:1)	r-GT	-0.0775	NS	-0.5640	0.0148*	-0.1777	NS	-0.1238	NS
PC(16:0p/20:1)	ALT	0.3166	NS	-0.5083	0.0313*	-0.1051	NS	-0.0115	NS
PC(18:0e/16:0)	LDH	-0.3186	NS	-0.6959	0.0013**	-0.2453	NS	0.0311	NS
PC(18:0e/18:0)	Neutrophils	-0.2428	NS	0.4902	0.0389*	0.0511	NS	-0.1566	NS
PC(18:0e/18:0)	TP	-0.2354	NS	-0.4837	0.0420*	0.0513	NS	0.1604	NS
PC(18:0e/18:0)	WBC	-0.2157	NS	0.6326	0.0048**	0.1102	NS	-0.0488	NS
PC(18:0p/14:0)	RDW	-0.2061	NS	-0.4689	0.0496*	0.0785	NS	-0.2460	NS
PC(18:0p/16:0)	LDH	-0.1961	NS	-0.5245	0.0254*	-0.2915	NS	-0.0040	NS
PC(18:0p/18:0)	TP	0.2624	NS	0.4910	0.0386*	-0.1604	NS	0.0062	NS
PC(18:0p/18:1)	Creatinine	-0.1687	NS	-0.4929	0.0377*	0.0545	NS	-0.5814	0.0004***
PC(18:0p/18:1)	RDW	0.4272	NS	-0.4803	0.0437*	0.0676	NS	-0.0433	NS
PC(18:1e/16:0)	LDH	0.0294	NS	-0.4739	0.0469*	-0.2138	NS	-0.0403	NS
PC(18:1e/16:0)	APTT	0.1974	NS	-0.4770	0.0453*	0.0216	NS	-0.2047	NS
PC(18:1e/18:1)	RBC	-0.0703	NS	-0.5552	0.0168*	-0.0289	NS	-0.5429	0.0011**
PC(18:1e/18:1)	Neutrophils	0.1951	NS	-0.5388	0.0211*	0.0101	NS	-0.4044	0.0196*
PC(18:1e/18:1)	Creatinine	0.2151	NS	-0.4801	0.0438*	-0.1423	NS	-0.5159	0.0021**
PC(18:2/20:3)	FIB	0.1127	NS	0.5926	0.0096**	-0.0927	NS	0.2424	NS
PC(18:2/20:3)	AST	0.2375	NS	-0.5161	0.0284*	0.1219	NS	-0.2974	NS

PC(18:2e/12:0)	Monocytes	-0.0824	NS	-0.6107	0.0071**	0.0913	NS	0.0714	NS
PC(18:2e/12:0)	TP	0.1619	NS	0.5003	0.0345*	-0.2279	NS	-0.0189	NS
PC(18:2e/16:0)	ALT	-0.1301	NS	0.5682	0.0139*	-0.0957	NS	0.2077	NS
PC(18:2e/16:0)	AST	-0.0185	NS	0.5254	0.0251*	-0.1771	NS	0.0916	NS
PC(18:2e/18:0)	globulin	-0.4194	NS	0.5661	0.0143*	-0.0541	NS	0.2113	NS
PE(16:0/18:0)	UA	-0.1815	NS	-0.5315	0.0232*	0.1224	NS	-0.2263	NS
PE(16:0/18:0)	Neutrophils	-0.1619	NS	-0.5666	0.0142*	-0.3217	NS	-0.3555	0.0423*
PE(16:0/18:0)	CRP	-0.1177	NS	-0.6001	0.0085**	0.1454	NS	-0.2606	NS
PE(16:0/18:0)	Monocytes	-0.0381	NS	-0.6957	0.0013**	-0.1074	NS	-0.4387	0.0107*
PE(16:0/18:0)	ALT	0.0626	NS	-0.6343	0.0047**	0.0731	NS	-0.1262	NS
PE(16:0/18:0)	CK-MB	0.0711	NS	-0.5583	0.0160*	0.0827	NS	0.0104	NS
PE(16:0/18:0)	WBC	0.1275	NS	-0.7379	0.0005***	-0.2982	NS	-0.4164	0.0159*
PE(16:0/18:0)	Creatinine	0.2990	NS	-0.5088	0.0311*	0.0548	NS	-0.2216	NS
PE(16:0/22:4)	APTT	0.0797	NS	0.4981	0.0354*	0.2494	NS	-0.1156	NS
PE(18:0/22:5)	PT	0.2748	NS	-0.5109	0.0302*	-0.2331	NS	-0.1365	NS
PE(18:0/22:5)	INR	0.2748	NS	-0.5109	0.0302*	-0.2331	NS	-0.1365	NS
PI(16:0/20:3)	WBC	-0.0110	NS	0.5457	0.0191*	0.0997	NS	0.0644	NS
PI(16:0/20:3)	Neutrophils	0.2636	NS	0.4950	0.0367*	0.1539	NS	0.0264	NS
SM(d18:1/18:4)	LDH	-0.2236	NS	-0.5484	0.0185*	-0.0476	NS	0.1226	NS
SM(d18:1/20:1)	globulin	-0.1189	NS	0.7231	0.0007***	0.1192	NS	-0.0358	NS
SM(d18:1/20:1)	PLT	0.0294	NS	0.5183	0.0275*	0.1832	NS	-0.2094	NS
SM(d18:1/20:2)	r-GT	-0.4219	NS	-0.6064	0.0076**	-0.0723	NS	-0.2346	NS
SM(d18:1/20:2)	ALT	-0.0589	NS	-0.6271	0.0053**	-0.0718	NS	-0.1041	NS
SM(d18:1/20:2)	globulin	-0.0221	NS	0.6033	0.0080**	0.1203	NS	-0.0269	NS
SM(d18:1/20:2)	UA	0.0037	NS	-0.4696	0.0493*	-0.1891	NS	-0.0204	NS
SM(d18:1/20:2)	AST	0.2831	NS	-0.5679	0.0140*	-0.3116	NS	-0.0613	NS

SM(d18:1/20:4)	Monocytes	-0.3085	NS	-0.4759	0.0459*	0.0004	NS	-0.1886	NS
SM(d18:1/20:4)	Ca	0.3988	NS	0.5795	0.0117*	0.0762	NS	0.1690	NS
SM(d18:1/20:5)	r-GT	-0.2706	NS	0.4873	0.0402*	-0.2856	NS	-0.0325	NS
SM(d18:1/20:5)	WBC	-0.0931	NS	0.4987	0.0351*	-0.1976	NS	-0.2731	NS
SM(d18:1/22:0)	ALT	-0.4209	NS	0.7283	0.0006***	0.0931	NS	0.1986	NS
SM(d18:1/22:0)	WBC	-0.4167	NS	0.4861	0.0408*	0.3248	NS	-0.0702	NS
SM(d18:1/22:0)	CRP	-0.3948	NS	0.5949	0.0092**	0.3048	NS	0.2893	NS
SM(d18:1/22:0)	TP	-0.3679	NS	-0.6543	0.0032**	-0.0847	NS	0.0487	NS
SM(d18:1/22:0)	r-GT	-0.2804	NS	0.6023	0.0082**	-0.0520	NS	0.1178	NS
SM(d18:1/22:0)	Neutrophils	-0.2685	NS	0.4737	0.0471*	0.3115	NS	-0.0657	NS
SM(d18:1/22:0)	globulin	-0.2146	NS	-0.5806	0.0115*	0.1506	NS	-0.0582	NS
SM(d18:1/22:0)	AST	-0.1575	NS	0.5969	0.0089**	0.1440	NS	0.2265	NS
SM(d18:1/22:3)	WBC	-0.4681	NS	0.5872	0.0104*	0.2772	NS	-0.0438	NS
SM(d18:1/22:3)	r-GT	-0.4539	NS	0.4959	0.0364*	-0.0053	NS	0.1342	NS
SM(d18:1/22:3)	CRP	-0.3127	NS	0.5670	0.0141*	0.2364	NS	0.3000	NS
SM(d18:1/22:3)	TP	-0.2403	NS	-0.6222	0.0058**	-0.0987	NS	-0.0283	NS
SM(d18:1/22:3)	globulin	-0.1202	NS	-0.5362	0.0218*	0.0327	NS	-0.0513	NS
SM(d18:1/22:4)	LDH	-0.0933	NS	-0.4765	0.0456*	0.1985	NS	0.0197	NS
SM(d18:1/22:4)	Ca	0.4815	NS	0.4746	0.0466*	0.0374	NS	0.1392	NS
TAG(48:1)	IBIL	-0.0567	NS	0.5016	0.0339*	-0.1747	NS	0.0483	NS
TAG(50:2)	P	-0.1490	NS	0.5772	0.0121*	-0.0993	NS	0.1929	NS
TAG(50:2)	Mg	-0.1423	NS	0.5339	0.0225*	0.2113	NS	-0.1912	NS
TAG(50:2)	Albumin	0.3509	NS	0.5758	0.0124*	0.2089	NS	0.2015	NS
TAG(52:5)	P	-0.0548	NS	-0.4899	0.0390*	0.1552	NS	-0.0659	NS
DAG(14:0/16:0)	AST	0.0406	NS	-0.3316	NS	-0.3829	0.0305*	-0.1183	NS
DAG(14:0/16:0)	HCT	0.0576	NS	0.1125	NS	-0.3821	0.0309*	0.4241	0.0139*

DAG(14:0/16:0)	UA	0.1140	NS	-0.3622	NS	-0.3683	0.0381*	0.1557	NS
DAG(14:0/16:0)	ALT	0.1521	NS	-0.2448	NS	-0.3857	0.0292*	-0.0974	NS
DAG(14:0/18:0)	DBIL	-0.2160	NS	-0.2614	NS	-0.3564	0.0453*	0.2061	NS
DAG(14:0/18:0)	HGB	-0.0319	NS	0.1643	NS	-0.4732	0.0062**	0.2118	NS
DAG(14:0/18:0)	RBC	-0.0098	NS	0.2260	NS	-0.4783	0.0056**	0.1400	NS
DAG(14:0/18:0)	HCT	0.0846	NS	0.1992	NS	-0.4975	0.0038**	0.1219	NS
DAG(14:0/18:0)	UA	0.1239	NS	-0.0196	NS	-0.3981	0.0240*	0.1414	NS
DAG(16:0/16:0)	BUN	-0.1582	NS	-0.0857	NS	-0.4467	0.0104*	0.0012	NS
DAG(16:0/16:0)	HCT	0.0846	NS	0.0010	NS	-0.3657	0.0395*	-0.4173	0.0157*
DAG(16:0/16:1)	HGB	-0.1263	NS	0.2718	NS	-0.4535	0.0091**	0.3769	0.0306*
DAG(16:0/16:1)	RBC	-0.0061	NS	0.4056	NS	-0.4541	0.0090**	0.3242	NS
DAG(16:0/16:1)	HCT	0.0012	NS	0.3271	NS	-0.4682	0.0069**	0.3607	0.0392*
DAG(16:1/16:1)	Monocytes	-0.2077	NS	-0.3847	NS	-0.3735	0.0352*	-0.0914	NS
DAG(16:1/16:1)	DBIL	-0.0687	NS	-0.2913	NS	-0.3933	0.0260*	0.3658	0.0363*
DAG(16:1/16:1)	HGB	-0.0380	NS	0.2501	NS	-0.4792	0.0055**	-0.0276	NS
DAG(16:1/16:1)	RBC	-0.0270	NS	0.1889	NS	-0.4087	0.0202*	-0.0648	NS
DAG(16:1/16:1)	AST	0.0418	NS	-0.1855	NS	-0.4607	0.0080**	-0.1705	NS
DAG(16:1/16:1)	HCT	0.0650	NS	0.2508	NS	-0.5204	0.0023**	-0.1737	NS
DAG(16:1/16:1)	UA	0.3225	NS	-0.0052	NS	-0.4073	0.0207*	-0.1464	NS
DAG(16:1/16:1)	Creatinine	0.3897	NS	0.1476	NS	-0.4841	0.0050**	-0.0184	NS
DAG(16:1/18:0)	FIB	-0.1143	NS	0.2286	NS	-0.3560	0.0455*	0.2860	NS
DAG(18:0/18:0)	PLT	-0.2130	NS	0.0953	NS	-0.3531	0.0474*	-0.0944	NS
DAG(18:0/18:2)	BUN	-0.3703	NS	-0.3395	NS	-0.3523	0.0480*	-0.1907	NS
DAG(18:0/18:2)	HCT	0.1790	NS	0.1909	NS	-0.3622	0.0416*	-0.2882	NS
DAG(18:1/20:0)	PLT	-0.1838	NS	-0.0196	NS	-0.4355	0.0127*	0.0358	NS
DAG(18:1/20:0)	α-HBDH	-0.0994	NS	-0.2672	NS	-0.3743	0.0348*	0.1976	NS

FFA(16:2)	PT	-0.1316	NS	-0.4091	NS	-0.3961	0.0248*	0.1267	NS
FFA(16:2)	INR	-0.1316	NS	-0.4091	NS	-0.3961	0.0248*	0.1267	NS
FFA(16:2)	APTT	0.0736	NS	-0.2282	NS	-0.4137	0.0186*	-0.3710	NS
FFA(20:3)	CO2	-0.2471	NS	0.2562	NS	-0.3745	0.0347*	-0.0174	NS
FFA(20:3)	APTT	0.4329	NS	-0.3397	NS	-0.3634	0.0409*	-0.0639	NS
FFA(20:4)	Mg	0.0468	NS	0.4508	NS	-0.3905	0.0399*	0.5426	0.0011**
FFA(20:4)	HCT	0.1030	NS	0.1393	NS	-0.4205	0.0165*	0.3950	0.0229*
FFA(20:4)	UA	0.3765	NS	-0.2301	NS	-0.4036	0.0220*	0.0622	NS
FFA(20:5)	HCT	0.1018	NS	0.1414	NS	-0.4297	0.0141*	0.3367	NS
FFA(20:5)	UA	0.3274	NS	-0.1723	NS	-0.4348	0.0129*	-0.0431	NS
FFA(22:3)	Neutrophils	-0.2894	NS	-0.2384	NS	-0.3769	0.0335*	0.4135	0.0168*
LPC(15:0)	AST	-0.4394	NS	-0.0187	NS	-0.3788	0.0325*	-0.2389	NS
LPC(18:1)	AST	-0.4170	NS	-0.0938	NS	-0.3691	0.0376*	-0.1249	NS
LPC(18:1)	HGB	-0.2122	NS	-0.4414	NS	-0.3664	0.0392*	-0.0386	NS
LPC(O/16:0)	AST	-0.4529	NS	0.0062	NS	-0.4055	0.0213*	-0.2732	NS
LPC(O/16:0)	HGB	-0.3752	NS	-0.1251	NS	-0.3528	0.0477*	-0.4541	0.0079**
LPE(14:0)	DBIL	-0.3816	NS	-0.3812	NS	-0.3700	0.0371*	-0.3534	0.0436*
LPE(14:0)	HGB	-0.3335	NS	0.1561	NS	-0.4250	0.0153*	-0.1577	NS
LPE(14:0)	TBIL	-0.2819	NS	-0.3032	NS	-0.3644	0.0403*	-0.5010	0.0030**
LPE(20:0)	Monocytes	0.2139	NS	-0.2976	NS	-0.4440	0.0109*	0.0632	NS
PC(14:0/16:0)	LDH	-0.3382	NS	-0.0888	NS	-0.4369	0.0201*	0.0482	NS
PC(16:0/20:3)	Creatinine	-0.1789	NS	0.2054	NS	-0.3771	0.0334*	0.1202	NS
PC(16:0/20:3)	AST	-0.0480	NS	-0.1658	NS	-0.3841	0.0300*	-0.2369	NS
PC(16:0e/16:1)	BUN	0.1312	NS	0.0320	NS	-0.4423	0.0113*	0.3846	0.0271*
PC(16:0e/18:1)	HGB	-0.3188	NS	-0.0868	NS	-0.4206	0.0165*	-0.1937	NS
PC(16:0e/18:1)	DBIL	-0.1939	NS	-0.1157	NS	-0.4030	0.0222*	-0.4250	0.0137*

PC(16:0e/18:1)	TBIL	-0.0760	NS	-0.1304	NS	-0.5516	0.0011**	-0.5431	0.0011**
PC(16:0e/18:1)	IBIL	-0.0637	NS	-0.1414	NS	-0.5347	0.0016**	-0.5336	0.0014**
PC(16:0e/20:2)	r-GT	-0.0627	NS	-0.3048	NS	-0.3928	0.0261*	-0.2314	NS
PC(16:0p/16:0)	BUN	-0.0147	NS	0.0526	NS	-0.3939	0.0257*	0.2335	NS
PC(16:0p/16:0)	Neutrophils	0.0723	NS	0.2900	NS	-0.3679	0.0383*	-0.0399	NS
PC(16:0p/16:0)	WBC	0.0882	NS	0.4035	NS	-0.3721	0.0360*	-0.0013	NS
PC(16:0p/16:0)	ALP	0.1104	NS	0.1910	NS	-0.3521	0.0481*	0.3185	NS
PC(16:0p/16:0)	UA	0.3213	NS	0.0815	NS	-0.3658	0.0395*	0.2824	NS
PC(16:0p/18:0)	HGB	-0.3372	NS	0.0145	NS	-0.4189	0.0170*	-0.3815	0.0285*
PC(16:0p/18:0)	DBIL	-0.2479	NS	-0.2810	NS	-0.4241	0.0156*	-0.4862	0.0041**
PC(16:0p/18:0)	IBIL	-0.1642	NS	-0.1785	NS	-0.4708	0.0065**	-0.3937	0.0234*
PC(16:0p/18:0)	TBIL	-0.1593	NS	-0.2225	NS	-0.5052	0.0032**	-0.4729	0.0054**
PC(16:0p/18:1)	Creatinine	-0.4191	NS	0.2487	NS	-0.3608	0.0425*	0.1825	NS
PC(16:0p/18:1)	HGB	-0.3384	NS	0.2605	NS	-0.4371	0.0124*	0.1683	NS
PC(16:0p/18:1)	HCT	-0.3274	NS	0.2652	NS	-0.3611	0.0423*	0.1687	NS
PC(16:0p/18:1)	RBC	-0.1962	NS	0.3209	NS	-0.5038	0.0033**	0.1878	NS
PC(16:0p/18:1)	UA	-0.1447	NS	0.3168	NS	-0.3985	0.0239*	0.1721	NS
PC(16:0p/18:3)	Creatinine	0.0392	NS	-0.1042	NS	-0.3830	0.0305*	-0.0560	NS
PC(16:0p/20:0)	TBIL	-0.4240	NS	-0.0704	NS	-0.4352	0.0128*	-0.0201	NS
PC(16:0p/20:0)	IBIL	-0.4167	NS	-0.1723	NS	-0.3808	0.0315*	-0.0445	NS
PC(16:0p/20:0)	DBIL	-0.3080	NS	0.1519	NS	-0.4344	0.0130*	0.1152	NS
PC(16:0p/20:0)	Creatinine	-0.2966	NS	0.1517	NS	-0.3663	0.0392*	0.0961	NS
PC(18:0e/18:0)	LDH	-0.2696	NS	0.1384	NS	-0.4492	0.0165*	-0.0206	NS
PC(18:0e/18:0)	FIB	0.1765	NS	0.1789	NS	-0.5377	0.0015**	-0.0181	NS
PC(18:0p/14:0)	BUN	-0.0417	NS	-0.0072	NS	-0.4740	0.0061**	0.1200	NS
PC(18:0p/16:0)	HGB	-0.4267	NS	-0.0992	NS	-0.4096	0.0199*	-0.2049	NS

PC(18:0p/16:0)	DBIL	-0.3055	NS	-0.1002	NS	-0.4254	0.0152*	-0.4331	0.0118*
PC(18:0p/16:0)	TBIL	-0.1961	NS	-0.1097	NS	-0.5430	0.0013*	-0.5284	0.0016**
PC(18:0p/16:0)	IBIL	-0.1716	NS	-0.1249	NS	-0.5198	0.0023*	-0.4998	0.0031**
PC(18:0p/18:0)	DBIL	-0.3644	NS	0.1756	NS	-0.4513	0.0095**	0.0763	NS
PC(18:0p/18:0)	AST	-0.2917	NS	0.0052	NS	-0.3692	0.0376*	-0.1308	NS
PC(18:0p/18:0)	Creatinine	-0.2206	NS	0.3375	NS	-0.3701	0.0371*	-0.0441	NS
PC(18:1e/16:0)	HGB	-0.3752	NS	-0.0031	NS	-0.3807	0.0316*	-0.2405	NS
PC(18:1e/16:0)	DBIL	-0.2135	NS	-0.1467	NS	-0.4120	0.0191*	-0.4568	0.0075**
PC(18:1e/16:0)	TBIL	-0.0858	NS	-0.1438	NS	-0.5182	0.0024**	-0.5582	0.0007***
PC(18:1e/16:0)	IBIL	-0.0686	NS	-0.1455	NS	-0.4961	0.0039**	-0.5314	0.0015**
PC(18:2/20:3)	ALP	-0.1166	NS	-0.2612	NS	-0.3804	0.0317*	-0.0507	NS
PC(18:2/20:3)	Mg	0.1108	NS	0.0385	NS	-0.3861	0.0424*	-0.1091	NS
PC(18:2/20:3)	DBIL	0.1693	NS	0.1054	NS	-0.3834	0.0303*	-0.2079	NS
PC(18:2/20:3)	Monocytes	0.4020	NS	0.1286	NS	-0.3586	0.0439*	-0.0381	NS
PC(18:2e/12:0)	DBIL	-0.4736	NS	-0.0124	NS	-0.4489	0.0100*	-0.0259	NS
PC(18:2e/12:0)	TBIL	-0.4118	NS	-0.1180	NS	-0.4587	0.0083**	0.0020	NS
PC(18:2e/12:0)	IBIL	-0.3725	NS	-0.1558	NS	-0.4226	0.0160*	0.0204	NS
PC(18:2e/12:0)	Creatinine	-0.2892	NS	-0.2260	NS	-0.3800	0.0319*	0.0814	NS
PC(18:2e/16:0)	Albumin	-0.2109	NS	0.1187	NS	-0.3501	0.0495*	0.2074	NS
PC(18:2e/16:0)	HGB	-0.1288	NS	0.2212	NS	-0.4866	0.0047**	0.2840	NS
PC(18:2e/16:0)	HCT	-0.1239	NS	0.2116	NS	-0.3648	0.0401*	0.3358	NS
PC(18:2e/16:0)	RBC	0.0086	NS	0.3209	NS	-0.4994	0.0036**	0.3419	NS
PC(18:2e/18:0)	HGB	-0.3679	NS	-0.0176	NS	-0.3511	0.0488*	-0.0940	NS
PC(18:2e/18:0)	Creatinine	-0.2794	NS	0.3953	NS	-0.3866	0.0288*	-0.0998	NS
PC(18:2e/18:0)	RBC	-0.2489	NS	0.2198	NS	-0.4527	0.0093**	-0.1604	NS
PC(18:2e/18:0)	r-GT	-0.1538	NS	-0.2521	NS	-0.3995	0.0235*	-0.2291	NS

PC(18:2e/18:0)	UA	0.0454	NS	0.0712	NS	-0.4652	0.0073**	-0.1324	NS
PC(18:2p/18:2)	AST	-0.2363	NS	-0.0021	NS	-0.3812	0.0314*	0.0655	NS
PE(18:0/22:5)	HGB	-0.1030	NS	0.2610	NS	-0.4809	0.0053**	-0.2451	NS
PE(18:0/22:5)	RBC	-0.0765	NS	0.2058	NS	-0.4306	0.0139*	-0.3551	0.0426*
PI(16:0/20:3)	Monocytes	-0.2168	NS	0.2891	NS	-0.4667	0.0071**	-0.0275	NS
SM(d18:1/18:4)	Creatinine	-0.4693	NS	0.2161	NS	-0.3764	0.0337*	0.0473	NS
SM(d18:1/18:4)	AST	-0.4146	NS	-0.0187	NS	-0.3553	0.0460*	0.0873	NS
SM(d18:1/18:4)	HGB	-0.3270	NS	-0.1243	NS	-0.3772	0.0333*	0.1240	NS
TAG(48:1)	Creatinine	0.1554	NS	0.4411	NS	-0.4349	0.0129*	0.3277	NS
TAG(50:2)	UA	-0.0074	NS	-0.0320	NS	-0.4632	0.0076**	-0.1354	NS
TAG(50:2)	Creatinine	0.1030	NS	0.1114	NS	-0.3524	0.0479*	-0.1247	NS
TAG(52:5)	Monocytes	-0.0688	NS	-0.1616	NS	-0.3920	0.0265*	0.0287	NS
DAG(14:0/16:0)	WBC	-0.3848	NS	-0.0155	NS	0.0451	NS	0.4709	0.0057**
DAG(14:0/16:0)	HGB	-0.0540	NS	0.0548	NS	-0.1944	NS	0.5035	0.0028**
DAG(14:0/16:0)	RBC	-0.0258	NS	0.2714	NS	-0.2317	NS	0.4024	0.0203*
DAG(14:0/16:0)	Creatinine	0.0564	NS	-0.0712	NS	-0.2637	NS	0.4008	0.0208*
DAG(14:0/18:0)	LDH	-0.2304	NS	-0.1094	NS	-0.1215	NS	0.3764	0.0308*
DAG(14:0/18:2)	HGB	0.0015	NS	0.2945	NS	-0.0658	NS	-0.4310	0.0123*
DAG(14:0/18:2)	HCT	0.0898	NS	0.3150	NS	-0.1030	NS	-0.3582	0.0407*
DAG(16:0/16:0)	RBC	0.0760	NS	-0.0980	NS	-0.1949	NS	-0.3493	0.0463*
DAG(16:0/16:1)	LDH	-0.2475	NS	-0.1466	NS	-0.0958	NS	0.3555	0.0423*
DAG(16:0/16:1)	Creatinine	-0.0858	NS	0.3602	NS	-0.2821	NS	0.4212	0.0147*
DAG(16:1/18:0)	PT	0.3358	NS	-0.1438	NS	-0.1328	NS	-0.5462	0.0047**
DAG(16:1/18:0)	INR	0.3358	NS	-0.1438	NS	-0.1328	NS	-0.5462	0.0047**
DAG(18:0/18:0)	α-HBDH	0.1738	NS	-0.0173	NS	-0.2114	NS	-0.4870	0.0136*
DAG(18:0/18:0)	LDH	0.1904	NS	-0.2638	NS	-0.1337	NS	0.3853	0.0268*

DAG(18:0/18:0)	Basophils	0.2415	NS	0.1456	NS	0.2854	NS	-0.4271	0.0132*
DAG(18:1/20:0)	PT	-0.2284	NS	-0.1807	NS	-0.1774	NS	-0.5792	0.0024**
DAG(18:1/20:0)	INR	-0.2284	NS	-0.1807	NS	-0.1774	NS	-0.5792	0.0024**
FFA(14:0)	α-HBDH	-0.1878	NS	0.1346	NS	-0.0215	NS	0.5180	0.0080**
FFA(14:0)	Basophils	-0.1463	NS	-0.0620	NS	0.2115	NS	0.4309	0.0123*
FFA(14:0)	P	-0.0775	NS	0.1467	NS	0.1194	NS	-0.4006	0.0209*
FFA(14:0)	ALT	0.0613	NS	-0.2211	NS	0.2522	NS	-0.3759	0.0311*
FFA(14:0)	TP	0.2134	NS	0.0393	NS	-0.1150	NS	-0.3629	0.0379*
FFA(14:0)	UA	0.2281	NS	-0.1971	NS	0.1998	NS	-0.4322	0.0120*
FFA(14:0)	RBC	0.2747	NS	-0.3478	NS	0.0757	NS	-0.4034	0.0199*
FFA(14:0)	Albumin	0.2943	NS	-0.0640	NS	0.0647	NS	-0.4006	0.0209*
FFA(14:0)	r-GT	0.3838	NS	0.1426	NS	0.2130	NS	-0.4253	0.0136*
FFA(14:0)	HGB	0.4647	NS	0.0393	NS	0.0730	NS	-0.3464	0.0483*
FFA(20:3)	Creatinine	-0.0956	NS	0.2632	NS	-0.1687	NS	0.3463	0.0484*
FFA(20:3)	HCT	-0.0773	NS	0.0052	NS	0.0154	NS	0.3862	0.0264*
FFA(20:3)	Mg	-0.0062	NS	-0.0156	NS	-0.1614	NS	0.3460	0.0486*
FFA(20:3)	UA	0.0196	NS	0.1084	NS	0.0462	NS	0.4445	0.0095**
FFA(20:3)	ALP	0.3387	NS	-0.3314	NS	-0.1495	NS	0.5159	0.0021**
FFA(20:4)	WBC	-0.1667	NS	-0.0485	NS	-0.0629	NS	0.3900	0.0248*
FFA(20:4)	CO2	-0.0184	NS	-0.1829	NS	0.0644	NS	0.4003	0.0385*
FFA(20:4)	RBC	0.0074	NS	0.2260	NS	-0.2605	NS	0.3777	0.0302*
FFA(20:4)	HGB	0.0466	NS	0.0972	NS	-0.2844	NS	0.4862	0.0041**
FFA(20:4)	PT	0.1155	NS	-0.4091	NS	0.0253	NS	-0.4606	0.0205*
FFA(20:4)	INR	0.1155	NS	-0.4091	NS	0.0253	NS	-0.4606	0.0205*
FFA(20:5)	CO2	-0.0492	NS	-0.2366	NS	0.0954	NS	0.4290	0.0255*
FFA(20:5)	Mg	0.0296	NS	0.3811	NS	-0.3575	NS	0.4723	0.0055**

FFA(20:5)	HGB	0.0319	NS	0.1003	NS	-0.3421	NS	0.4673	0.0061**
FFA(20:5)	PT	0.1217	NS	-0.4351	NS	0.0670	NS	-0.4633	0.0197*
FFA(20:5)	INR	0.1217	NS	-0.4351	NS	0.0670	NS	-0.4633	0.0197*
FFA(22:3)	UA	-0.1214	NS	0.3044	NS	0.0883	NS	0.3633	0.0377*
FFA(22:5)	HCT	-0.3323	NS	-0.2528	NS	0.1483	NS	-0.4228	0.0142*
FFA(22:5)	FIB	-0.1054	NS	0.1107	NS	-0.1526	NS	0.4039	0.0453*
FFA(22:5)	Glucose	-0.0785	NS	-0.0568	NS	0.1098	NS	0.3614	0.0388*
HexCer(d18:1/22:1)	LDH	-0.3921	NS	0.0253	NS	0.0124	NS	-0.3961	0.0225*
HexCer(d18:1/22:1)	ALP	-0.3292	NS	0.1963	NS	0.2593	NS	-0.3484	0.0469*
HexCer(d18:1/22:1)	TT	-0.2032	NS	0.2942	NS	0.2557	NS	-0.4880	0.0133*
HexCer(d18:1/22:1)	TP	-0.0906	NS	0.1308	NS	-0.0474	NS	-0.3568	0.0415*
HexCer(d18:1/22:1)	Albumin	0.1117	NS	-0.0501	NS	-0.1156	NS	-0.3844	0.0272*
HexCer(d18:1/22:1)	FIB	0.2705	NS	-0.4118	NS	-0.1781	NS	0.5575	0.0038**
LPC(15:0)	HGB	-0.4255	NS	-0.0806	NS	-0.3452	NS	-0.4222	0.0144*
LPC(15:0)	TBIL	-0.3799	NS	-0.4025	NS	-0.1610	NS	-0.3590	0.0402*
LPC(15:0)	HCT	-0.3433	NS	-0.0753	NS	-0.0878	NS	-0.5314	0.0015**
LPC(15:0)	RBC	-0.2722	NS	0.2384	NS	-0.1590	NS	-0.4469	0.0091**
LPC(15:0)	Albumin	-0.2269	NS	-0.0124	NS	-0.1779	NS	-0.4217	0.0145*
LPC(16:0)	CRP	-0.3568	NS	0.2721	NS	0.1408	NS	0.4353	0.0114*
LPC(16:1)	CRP	-0.2748	NS	0.3735	NS	0.1885	NS	0.3584	0.0406*
LPC(16:1)	Basophils	-0.1729	NS	0.1894	NS	0.2976	NS	-0.4320	0.0120*
LPC(18:0)	Mg	0.0468	NS	0.3571	NS	-0.1246	NS	0.3965	0.0224*
LPC(18:0)	Basophils	0.0892	NS	-0.0895	NS	0.2872	NS	-0.3525	0.0442*
LPC(18:1)	APTT	-0.1461	NS	-0.0560	NS	0.3356	NS	0.4235	0.0349*
LPC(18:1)	TT	0.3152	NS	0.1490	NS	0.0907	NS	-0.4229	0.0352*
LPC(18:3)	α-HBDH	0.3499	NS	-0.1150	NS	0.1150	NS	0.4699	0.0178*

LPC(22:1)	Albumin	-0.3703	NS	-0.0712	NS	-0.0992	NS	-0.4119	0.0172*
LPC(22:1)	PT	-0.1552	NS	-0.1392	NS	0.2152	NS	0.5746	0.0027**
LPC(22:1)	INR	-0.1552	NS	-0.1392	NS	0.2152	NS	0.5746	0.0027**
LPC(22:1)	Lymphocytes	-0.0858	NS	-0.1352	NS	0.0664	NS	-0.3707	0.0337*
LPC(O/16:0)	DBIL	-0.4761	NS	-0.3337	NS	-0.2228	NS	-0.3685	0.0349*
LPC(O/16:0)	HCT	-0.2796	NS	-0.1084	NS	-0.1386	NS	-0.5695	0.0005***
LPC(O/16:0)	Albumin	-0.1888	NS	-0.0847	NS	-0.1781	NS	-0.3809	0.0288*
LPC(O/16:0)	RBC	-0.1643	NS	0.2178	NS	-0.1764	NS	-0.4779	0.0049**
LPC(O/18:0)	Albumin	-0.2906	NS	0.0650	NS	-0.0691	NS	-0.4101	0.0178*
LPC(O/18:0)	RBC	-0.2244	NS	0.2570	NS	-0.2327	NS	-0.3733	0.0324*
LPC(O/18:0)	ALP	0.1840	NS	-0.1817	NS	0.1295	NS	-0.3463	0.0484*
LPE(14:0)	IBIL	-0.2892	NS	-0.1806	NS	-0.3350	NS	-0.5642	0.0006***
LPE(14:0)	Glucose	-0.0037	NS	-0.0733	NS	0.0936	NS	0.4261	0.0134*
LPE(20:0)	Basophils	0.1059	NS	0.3823	NS	-0.0339	NS	0.3441	0.0499*
LPE(20:3)	IBIL	-0.4583	NS	-0.2178	NS	-0.0644	NS	-0.4218	0.0145*
LPE(20:4)	Creatinine	-0.4436	NS	0.2301	NS	-0.1003	NS	-0.4065	0.0189*
LPE(20:4)	TBIL	-0.4436	NS	-0.2359	NS	0.0075	NS	-0.4445	0.0095**
LPE(20:4)	IBIL	-0.3922	NS	-0.1827	NS	0.0556	NS	-0.3524	0.0443*
LPE(20:4)	HCT	-0.3421	NS	0.0712	NS	-0.1241	NS	-0.4298	0.0126*
LPE(20:5)	CRP	-0.3225	NS	0.2442	NS	0.1779	NS	0.3724	0.0328*
LPE(20:5)	HCT	-0.2342	NS	0.0877	NS	-0.1787	NS	0.3480	0.0472*
LPE(20:5)	PT	0.1589	NS	0.1298	NS	0.2959	NS	0.4428	0.0266*
LPE(20:5)	INR	0.1589	NS	0.1298	NS	0.2959	NS	0.4428	0.0266*
LPE(22:6)	DBIL	-0.4356	NS	-0.2045	NS	-0.2015	NS	-0.4309	0.0123*
MAG(22:5)	ALP	0.2626	NS	0.1311	NS	-0.0007	NS	-0.3536	0.0435*
MAG(22:5)	CO2	0.3897	NS	-0.3471	NS	0.2551	NS	0.4299	0.0252*

PA(16:0/18:0)	BUN	-0.1755	NS	0.2953	NS	0.0400	NS	0.3495	0.0462*
PC(14:0/16:0)	DBIL	-0.0368	NS	-0.2676	NS	-0.3300	NS	-0.5629	0.0006***
PC(14:0/16:0)	TBIL	0.1078	NS	-0.2162	NS	-0.2546	NS	-0.5434	0.0011**
PC(14:0/16:0)	IBIL	0.1152	NS	-0.1889	NS	-0.2010	NS	-0.5222	0.0018**
PC(16:0/20:0)	TBIL	0.0417	NS	0.1148	NS	0.1490	NS	-0.3676	0.0353*
PC(16:0/20:0)	HCT	0.0478	NS	-0.2363	NS	-0.0627	NS	-0.3557	0.0422*
PC(16:0/20:0)	DBIL	0.1681	NS	0.1612	NS	-0.0150	NS	-0.4782	0.0049**
PC(16:0/20:0)	FIB	0.3088	NS	0.3071	NS	-0.2765	NS	0.4763	0.0161*
PC(16:0/20:3)	CO2	0.0910	NS	0.2531	NS	0.0229	NS	-0.4052	0.0360*
PC(16:0e/16:0)	PLT	0.0515	NS	0.4326	NS	0.3221	NS	-0.3799	0.0292*
PC(16:0e/18:0)	Albumin	-0.0822	NS	-0.0785	NS	-0.1586	NS	-0.3705	0.0338*
PC(16:0e/18:1)	Glucose	-0.0552	NS	-0.0361	NS	0.0063	NS	0.4291	0.0127*
PC(16:0e/20:2)	HCT	-0.3299	NS	-0.1723	NS	-0.1413	NS	-0.4970	0.0033**
PC(16:0e/20:2)	HGB	-0.2857	NS	-0.1592	NS	-0.2338	NS	-0.4304	0.0124*
PC(16:0e/20:2)	RBC	-0.2085	NS	-0.0526	NS	-0.3137	NS	-0.4686	0.0060**
PC(16:0e/20:2)	Creatinine	-0.1029	NS	0.0836	NS	-0.3118	NS	-0.4070	0.0188*
PC(16:0e/20:2)	WBC	0.2010	NS	0.0382	NS	0.0891	NS	-0.3640	0.0373*
PC(16:0e/20:4)	Albumin	-0.1275	NS	0.0382	NS	0.0194	NS	0.4121	0.0172*
PC(16:0e/20:4)	Basophils	-0.0544	NS	0.0723	NS	0.2276	NS	-0.4412	0.0102*
PC(16:0e/20:4)	TP	-0.0441	NS	-0.0879	NS	-0.2243	NS	0.3614	0.0388*
PC(16:0e/20:4)	ALP	0.1399	NS	-0.0114	NS	0.0616	NS	0.4287	0.0128*
PC(16:0e/20:4)	P	0.2129	NS	-0.1539	NS	-0.1602	NS	0.3773	0.0304*
PC(16:0p/16:0)	Basophils	0.1644	NS	0.2101	NS	-0.1512	NS	-0.3797	0.0293*
PC(16:0p/16:0)	P	0.2905	NS	0.0227	NS	-0.2531	NS	0.4432	0.0098**
PC(16:0p/18:0)	HCT	-0.3519	NS	0.0237	NS	-0.1516	NS	-0.5067	0.0026**
PC(16:0p/18:0)	RBC	-0.3311	NS	0.1331	NS	-0.2105	NS	-0.3727	0.0327*

PC(16:0p/18:0)	FIB	-0.0319	NS	-0.0941	NS	-0.1413	NS	0.4154	0.0389*
PC(16:0p/18:0)	Ca	0.1092	NS	-0.0217	NS	0.0935	NS	0.3615	0.0387*
PC(16:0p/18:1)	Neutrophils	-0.3985	NS	0.0506	NS	-0.0374	NS	0.3500	0.0459*
PC(16:0p/20:0)	RDW	-0.1296	NS	0.0455	NS	0.0658	NS	0.3769	0.0306*
PC(16:0p/20:1)	HCT	-0.2710	NS	-0.3808	NS	-0.1025	NS	-0.3825	0.0280*
PC(16:0p/20:1)	Basophils	-0.2662	NS	-0.2583	NS	0.1638	NS	0.3591	0.0402*
PC(16:0p/20:1)	HGB	-0.2587	NS	-0.3907	NS	-0.1164	NS	-0.3536	0.0435*
PC(16:0p/20:1)	RBC	-0.1704	NS	-0.2178	NS	-0.2471	NS	-0.3971	0.0221*
PC(16:0p/20:1)	Creatinine	-0.0882	NS	-0.1847	NS	-0.1474	NS	-0.4049	0.0194*
PC(16:0p/20:1)	UA	0.1006	NS	-0.4097	NS	-0.1642	NS	-0.3539	0.0433*
PC(16:0p/22:6)	Basophils	-0.1129	NS	0.0138	NS	0.2210	NS	-0.3951	0.0229*
PC(18:0e/16:0)	Albumin	-0.0159	NS	-0.0145	NS	-0.1450	NS	-0.3474	0.0476*
PC(18:0e/18:0)	HCT	-0.4758	NS	0.1827	NS	0.0033	NS	-0.3758	0.0311*
PC(18:0e/18:0)	TBIL	-0.3211	NS	0.1076	NS	0.0280	NS	-0.3513	0.0450*
PC(18:0e/18:0)	IBIL	-0.2868	NS	0.2632	NS	0.0524	NS	-0.3602	0.0395*
PC(18:0e/18:0)	Glucose	-0.0576	NS	0.0382	NS	0.0706	NS	0.3923	0.0239*
PC(18:0e/18:0)	BUN	-0.0540	NS	0.2446	NS	-0.1248	NS	0.5106	0.0024**
PC(18:0p/16:0)	Glucose	-0.1496	NS	0.0237	NS	0.0706	NS	0.3875	0.0259*
PC(18:0p/18:1)	DBIL	-0.3541	NS	-0.2744	NS	0.1099	NS	-0.4263	0.0134*
PC(18:0p/18:1)	Basophils	-0.2667	NS	-0.0328	NS	0.1211	NS	0.3618	0.0386*
PC(18:0p/18:1)	UA	-0.2545	NS	-0.2176	NS	0.1411	NS	-0.4038	0.0198*
PC(18:0p/18:1)	Ca	-0.0174	NS	0.2455	NS	0.2930	NS	0.4704	0.0057**
PC(18:0p/18:1)	Neutrophils	-0.0149	NS	0.0516	NS	-0.0489	NS	-0.3829	0.0278*
PC(18:0p/18:1)	HGB	-0.0074	NS	-0.3016	NS	-0.0151	NS	-0.5883	0.0003***
PC(18:0p/18:1)	HCT	0.0459	NS	-0.3380	NS	0.1513	NS	-0.6550	0.0000***
PC(18:0p/18:1)	RBC	0.0670	NS	-0.3638	NS	0.1366	NS	-0.6344	0.0001 ***

PC(18:1e/16:0)	Glucose	-0.0601	NS	0.0093	NS	0.0378	NS	0.4027	0.0202*
PC(18:1e/18:1)	DBIL	-0.2010	NS	-0.2338	NS	0.0132	NS	-0.3878	0.0258*
PC(18:1e/18:1)	Ca	-0.0057	NS	0.4077	NS	0.2563	NS	0.4429	0.0099**
PC(18:1e/18:1)	HCT	0.0832	NS	-0.3580	NS	-0.0082	NS	-0.6572	0.0000***
PC(18:1e/18:1)	HGB	0.0918	NS	-0.3468	NS	-0.0479	NS	-0.5223	0.0018**
PC(18:1e/18:1)	WBC	0.2294	NS	-0.3979	NS	0.0523	NS	-0.3825	0.0280*
PC(18:2e/12:0)	Glucose	-0.1496	NS	0.1228	NS	0.2743	NS	0.3577	0.0410*
PC(18:2e/16:0)	Neutrophils	-0.0993	NS	0.0361	NS	-0.0651	NS	0.4274	0.0131*
PC(18:2e/16:0)	Basophils	-0.0892	NS	-0.0723	NS	0.1372	NS	-0.3993	0.0213*
PC(18:2p/18:2)	RBC	-0.0773	NS	0.3189	NS	-0.2030	NS	0.3449	0.0493*
PC(18:2p/20:4)	Basophils	-0.1129	NS	0.0620	NS	0.2451	NS	-0.3914	0.0243*
PE(16:0/18:0)	Lymphocytes	0.0748	NS	-0.4427	NS	-0.1298	NS	-0.3514	0.0449*
PE(16:0/22:4)	UA	-0.3479	NS	-0.3056	NS	-0.0825	NS	-0.4091	0.0181*
PE(16:0/22:4)	DBIL	-0.1129	NS	-0.2335	NS	-0.0906	NS	-0.3860	0.0265*
PE(16:0/22:4)	Basophils	-0.0905	NS	-0.3964	NS	0.1561	NS	0.3592	0.0401*
PE(18:0/22:5)	Monocytes	-0.4348	NS	-0.1330	NS	-0.1521	NS	-0.4192	0.0152*
PE(18:0/22:5)	TBIL	-0.3798	NS	-0.2115	NS	-0.2896	NS	-0.3661	0.0361*
PE(18:0/22:5)	DBIL	-0.3704	NS	-0.2909	NS	-0.2434	NS	-0.3440	0.0490*
PE(18:0/22:5)	IBIL	-0.3440	NS	-0.1044	NS	-0.2775	NS	-0.3836	0.0275*
PE(18:0/22:5)	TT	-0.1004	NS	0.0497	NS	-0.1276	NS	-0.4223	0.0355*
PE(18:0/22:5)	CK-MB	-0.0382	NS	-0.1706	NS	-0.1392	NS	-0.4460	0.0093**
PE(18:0/22:5)	FIB	0.2972	NS	0.2290	NS	0.1384	NS	0.4408	0.0274*
PI(16:0/20:3)	CRP	-0.2663	NS	0.3648	NS	0.0428	NS	0.3977	0.0219*
PI(18:0/20:3)	CO2	-0.0697	NS	0.2777	NS	-0.1449	NS	0.4601	0.0157*
SM(d18:1/16:0)	P	0.3553	NS	-0.0557	NS	0.2155	NS	-0.4400	0.0104*
SM(d18:1/18:2)	RBC	-0.3691	NS	-0.0808	NS	0.0507	NS	-0.4922	0.0036**

SM(d18:1/18:2)	HCT	-0.2999	NS	0.2040	NS	0.0413	NS	-0.5589	0.0007***
SM(d18:1/18:2)	HGB	-0.2010	NS	0.2036	NS	0.0525	NS	-0.4615	0.0069**
SM(d18:1/18:2)	Creatinine	0.0560	NS	-0.1162	NS	-0.0265	NS	-0.5530	0.0008***
SM(d18:1/18:2)	Neutrophils	0.2735	NS	-0.1389	NS	-0.1346	NS	-0.4594	0.0072**
SM(d18:1/18:2)	WBC	0.3326	NS	-0.0737	NS	-0.1202	NS	-0.3984	0.0216*
SM(d18:1/18:2)	DBIL	0.4286	NS	-0.1135	NS	-0.1314	NS	-0.3521	0.0445*
SM(d18:1/18:3)	Glucose	-0.0810	NS	0.2797	NS	-0.1213	NS	0.3590	0.0402*
SM(d18:1/18:4)	Basophils	0.0810	NS	-0.0104	NS	0.1610	NS	-0.3612	0.0389*
SM(d18:1/22:0)	DBIL	-0.1006	NS	-0.2107	NS	-0.1009	NS	-0.4540	0.0080**
SM(d18:1/22:0)	TBIL	0.0466	NS	-0.0217	NS	-0.1129	NS	-0.4769	0.0050**
SM(d18:1/22:0)	IBIL	0.0588	NS	0.1434	NS	-0.1117	NS	-0.5067	0.0026**
SM(d18:1/22:0)	TT	0.1921	NS	0.3649	NS	0.0336	NS	-0.4475	0.0249*
SM(d18:1/22:3)	DBIL	-0.3485	NS	-0.2025	NS	-0.0976	NS	-0.4428	0.0099**
SM(d18:1/22:3)	TBIL	-0.2426	NS	-0.0135	NS	-0.1223	NS	-0.4840	0.0043**
SM(d18:1/22:3)	IBIL	-0.2181	NS	0.1765	NS	-0.1225	NS	-0.5147	0.0022**
SM(d18:1/22:3)	TT	0.2069	NS	0.4103	NS	0.2210	NS	-0.4607	0.0205*
TAG(50:0)	RBC	-0.0137	NS	-0.1734	NS	-0.0374	NS	-0.5091	0.0025**
TAG(50:0)	Ca	0.0298	NS	-0.3004	NS	-0.1599	NS	0.4054	0.0193*
TAG(50:0)	HGB	0.0447	NS	-0.0621	NS	0.1275	NS	-0.5317	0.0014**
TAG(50:0)	HCT	0.0497	NS	-0.0851	NS	-0.0898	NS	-0.5867	0.0003***
TAG(50:0)	Neutrophils	0.1167	NS	-0.0399	NS	0.2424	NS	-0.4302	0.0125*
TAG(50:0)	WBC	0.3499	NS	-0.1461	NS	0.1692	NS	-0.3488	0.0467*
TAG(50:2)	Eosinophils	-0.0399	NS	-0.4548	NS	0.0865	NS	0.3988	0.0215*

Abbreviation: **Abbreviation**: ALP, alkaline phosphatase; ALT, alanine aminotransferase; APTT, activated partial thromboplastin time; AST, aspartate aminotransferase; BUN, blood urea nitrogen; Ca, calcium; CK-MB, creatine kinase-myocardial band; CRP, C-reactive protein; DAG, diacylglycerol; DBIL,

direct bilirubin; FIB, FFA, free fatty acid; fibrinogen; HCs, healthy controls; IBIL, indirect bilirubin; INR, international normalized ratio; LDH, lactate dehydrogenase; LPC, lysoPhosphatidylcholine; LPE, lysoPhosphatidylethanolamine; M, moderate; MCH, mean corpuscular hemoglobin; MCHC, mean corpuscular hemoglobin concentration; MCV, mean corpuscular volume; Mg, magnesium; P, phosphorus; PC, phosphatidylcholine; PCT, plateletocrit; PDW, platelet distribution width; PE, phosphatidylethanolamine; PT, prothrombin time; PI, phosphatidylinositol; RAs, recovered asymptomatic patients; RBC, red blood cell; RDW, red blood cell volume distribution width; RMs, recovered moderate patients; RSs, recovered critical and severe patients; S, critical and severe; SM, sphingomyelin; TAG, triacylglycerol; TBIL, total bilirubin; TT, thrombin time; UA, urine acid; WBC, white blood cell; α-HBDH, α-hydroxybutyrate dehydrogenase; γ-GT, γ-glutamyl transpeptidase; Statistical significance was set at 2-sided. *$p<0.05$, **$p<0.01$, ***$p<0.001$.
